# Impact of cilia length and variable fluid properties on electroosmotic nanofluid flow in an inclined converging microchannel

**DOI:** 10.1038/s41598-025-30669-z

**Published:** 2025-12-13

**Authors:** Pooja Sadalagi, Rajashekhar Choudhari, Hanumesh Vaidya, Manjunatha Gudekote, Kerehalli Vinayak Prasad

**Affiliations:** 1 Department of Mathematics, Manipal Institute of Technology Bengaluru, Manipal Academy of Higher Education, Manipal, Karnataka India; 2 Department of Mathematics, Vijayanagara Sri Krishnadevaraya University, Ballari, 583105 Karnataka India; 3 Department of Mathematics, Manipal Institute of Technology, Manipal Academy of Higher Education, Manipal, India

**Keywords:** Inclined converging microchannel, Buongiorno nanofluid, Cilia length, Metachronal wave, Variable fluid properties, Good health and well-being, Engineering, Mathematics and computing, Nanoscience and technology, Physics

## Abstract

This study presents a mathematical model for the transport of non-Newtonian nanofluids in an inclined ciliated converging microchannel. The analysis focuses on the combined effects of cilia length variation, electroosmotic effect, and temperature-dependent viscosity and thermal conductivity. The governing equations were derived using the Debye–Hückel approximation along with lubrication theory. These equations were then solved semi-analytically using the Homotopy Perturbation Method (HPM) in MATHEMATICA. The resulting solutions were visualized by plotting graphs in MATLAB. The results indicate that increased cilia length leads to a reduction in axial velocity but lowers the external pressure required to maintain flow, allowing for precise adjustments to transport dynamics. Variable viscosity and thermal conductivity improve flow and heat transfer under mild obstruction. The applied electric field accelerates the fluid by offsetting the drag caused by cilia, thereby enhancing overall transport efficiency. These findings illustrate the capability of cilia to serve as moderators of flow and transport in applications like targeted drug delivery, lab-on-a-chip diagnostics, microscale heat exchangers, and bio-inspired pumping systems.

**Supplementary Information:**

The online version contains supplementary material available at 10.1038/s41598-025-30669-z.

## Introduction

Efficient fluidic transport in confinement geometry is a cornerstone of many biological processes, as well as in microscale engineering devices. The processes that include clearance of the mucus in the respiratory tract, nutrient movement in the gastrointestinal system, and circulation in embryonic tissues depend on efficient control of internal flow. Similarly, microfluidic systems, lab-on-chip devices, and biomedical implants require the accurate control of fluids at very small scales. But at low Reynolds number, where viscous forces prevail and inertial forces are secondary, the conventional ways of pumping are inefficient, as the Stokes flow is reversible. To counter this, nature has non-reciprocal methods, the most notable being peristalsis, which is the deformation of the walls^1,2^ and non-reciprocal ciliary beating, providing unidirectional movement in highly viscous environments. Such bio-inspired approaches have inspired the development of artificial microscale pumping systems that can recapitulate such efficient transport systems.

Cilia-based propulsion is a more sophisticated method of propulsion, where microscopic hair-like extensions (cilia) generate synchronized movements that effectively allow the movement of fluids and suspended particles. These systems play a crucial role in physiological processes, including the removal of mucus from respiratory passages and the transfer of ova in fallopian tubes, which has inspired the development of artificial microfluidic transport and selective drug delivery systems^3^. The ciliary beat consists of two distinct stages: the effective stroke and the recovery stroke. During the effective stroke, the cilium is stretched fully, and a large amount of fluid is pushed forward. When the cilium begins to move forward, the groove lip in which it is attached temporarily causes restraints to the movement of the cilium^4^. Conversely, during the recovery stroke, the cilium contracts by bending towards itself and the nearest boundary, thereby dragging the fluid back in the opposite direction^5^. Effective stroke, especially in most cases, is significantly shorter than the recovery stroke^4^. The beating of the cilia is synchronized to form an outward appearance that appears to move and twist in a wave-like pattern, known as metachronal waves^6^. Such waves are due to a phase lag of the nearest cilia^7^. Another reason that contributes to metachronism is hydrodynamic coupling, which enables bacteria such as Paramecium and Volvox to navigate dense environments^8^. Low Reynolds number regimes have been extensively studied to determine transport velocities and shear distributions in cilia-driven flows. The envelope model, introduced by Blake^9^, enables the estimation of net pumping rates in viscous fluids analytically by allowing for the calculation of cilia layers. This was further developed by Fulford and Blake^10^, who evaluated the influence of the cilia separation, beat frequency, and tilt on the flow properties near the cilia. These studies formed the basis of hydrodynamic principles, which were later applied in the study of microfluidic and biological transportation. The dynamics of cilia, particularly changes in cilia length and the balanced stimulation and synchronous movement of cilia, have been extensively studied in biological systems. However, in the majority of theoretical and experimental methods, cilia are assumed to be uniform in length, which does not describe the spatial changes observed in real systems. Cilia lengths vary in biological structures, such as the respiratory system and efferent ducts, which affect local shear and flow patterns. Despite the importance of this factor, the implications of variability in cilia length have not been adequately considered by current models. This omission impairs our ability to fully learn and recapitulate natural transport in biomimetic microfluidic use.

In addition to the ciliary propulsion, another important mode of transporting fluid in micro/nano devices is the electroosmotic flow (EOF). A channel containing electrolyte and charged walls generates the movement of mobile ions by applying an electric field across the channel, and causes the movement of bulk fluids through viscous coupling. Biomicrofluidic devices have also been operated using electroosmotic pumping, such as silicon-based micropumps and systems that are actuated by electrostatic or thermopneumatic forces to control the flow^11^. It has been proven by numerous investigators^12,13^ that the impact of changing the electric field and geometry of channels has a significant influence on the behavior of EOF. Electroosmotic flow (EOF), in addition to ciliary propulsion, is a potent mechanism for liquid transportation in micro/nano devices, especially at low Reynolds numbers. However, in cases where cilia are either long or closely packed, they can create significant resistance, thereby reducing flow efficiency. Under such circumstances, EOF can be regarded as a countermeasure to the drag induced by the extended cilia. A more effective and controlled movement of a fluid in a constricted microchannel can be achieved by combining electroosmosis with variable cilia length. A combination of these developments, along with the growth of nanofluids, has enabled the use of nanofluids to enhance heat and mass transfer in biomedical and engineering applications. The high thermal conductivity and energy transfer are greatly enhanced by the presence of nanoparticles in a base fluid. The theory of Buongiorno^14^ is one of the most physically realistic theories of nanofluids that includes Brownian motion and thermophoresis as the primary physical forces driving the diffusion of nanoparticles. The model has been particularly applied in cases with physiological flow where the temperature variation and trapping of particles are of interest^15,16^.

The other property of realism in microscale flow modeling on the micro level is the capability of considering the temperature-dependent fluid properties, such as the viscosity^17,18^ and thermal conductivity^19^. Non-uniform rheological behavior is observed in biological fluids such as blood and mucus; it is affected by the local temperature and concentration variations. These factors should not be ignored as they can lead to wrong predictions especially in the case of cilia induced or electroosmotic flow. Earlier research by Elogail and Elshekipy^20^ and, similarly, Bhatti et al.^21^ extends on the importance of considering the thermal differences to obtain the precision of the modeling outcome. Moreover, most studies on ciliary flow have been conducted in straight and uniform channels, whereas, in reality, most conduits are either convergent or divergent. The convergent-inclined channel geometry is a crucial component of physical significance, as it combines the properties of tapering and inclination, which in turn influence the pressure, velocity, and shear distributions in the flow. The inclination introduces an element of body force that alters the direction and magnitude of fluid movement, while convergence enhances acceleration and mixing. These factors collectively affect the hydrodynamic status of the cilia and their transport efficiency. It offers more control over the flow behavior and a more realistic representation of natural biological conduits, and it finds application in microfluidic systems, such as drug delivery, micropumping, and lab-on-chip systems^22^.

To overcome these unresolved problems, the present study specifically examines the impacts of different cilia lengths along the channel wall, providing a theoretical basis for the flow transduced by cilia in a convergent inclined microchannel. Also, the model employs temperature-dependent viscosity and thermal conductivity. The rationale of this solution is the necessity to mimic the physiological reality of the effect of this variation on local shear and pumping efficiency, which subsequently influences important processes in microfluidic and biomedical systems. In addition, the impact of electro-osmotic flow is incorporated, facilitating the examination of the interaction between externally applied electric fields and ciliary motion to improve transport in low Reynolds number conditions. To better understand the behaviors of heat and mass transfer at the nanoscale, we utilize the Buongiorno nanofluid model. This study fills a critical gap in existing literature, where most models assume uniform cilia lengths, constant fluid properties, and neglect electrokinetics or nanoscale effects in complex geometries (Table 1).

In order to overcome the above-mentioned limitations in the current models, the following research questions will be answered in the present study:

- What is the effect of spatial change in the length of cilia on the local shear stress, flow rate, and the performance of the pump in a convergent inclined microchannel?
- How do the electroosmotic forces, in conjunction with the movement of the cilia, affect the fluid transportation efficiency in the low Reynolds number regime?
- What is the influence of temperature-dependent viscosity and thermal conductivity on velocity, temperature, and nanoparticle concentration distributions in cilia-driven electroosmotic flow?
- How do the mechanisms of Brownian motion, thermophoresis, though modeled by the Buongiorno nanofluid, change the nature of heat and mass transfer in microscale confined geometries?

### Novelty and distinction from previous studies

- This model uses spatially varying cilia length in contrast to the previous models, which assumed equal cilia length^14,23^ and showed their effects on the local shear and pumping efficiency.
- Previous analysis was done using straight channels^24^ and the current analysis assumes a convergent-inclined geometry to introduce realistic effects of acceleration and confinement.
- The majority of the available models use constant viscosity and thermal conductivity^25^, whereas in the present study, these properties are treated as temperature-dependent to capture biological and microscale flow conditions.
- Previous studies had discussed either electroosmosis or cilia motions individually^26,27^ but this study unites the two processes to further develop fluid control at low Reynolds numbers.

**Table 1:** Comparative analysis of modelling characteristics that emphasises the new insights this study offers over earlier research.

Effects	Munawar et al.^28^	Mishra et al.^25^	Rafiq et al.^29^	Allehiany et al.^16^	Akbar et al.^30^	Present work
Inclined channel geometry	$\:\times\:$	$\:\times\:$	**✓**	**✓**	$\:\times\:$	**✓**
Converging wall configuration	$\:\times\:$	$\:\times\:$	$\:\times\:$	$\:\times\:$	$\:\times\:$	**✓**
Electro-osmosis effect	**✓**	**✓**	$\:\times\:$	**✓**	$\:\times\:$	**✓**
Variable thermal conductivity with respect to temperature	$\:\times\:$	$\:\times\:$	$\:\times\:$	$\:\times\:$	$\:\times\:$	**✓**
Variable viscosity with respect to temperature	$\:\times\:$	$\:\times\:$	$\:\times\:$	$\:\times\:$	**✓**	**✓**
Cilia-actuated flow	**✓**	**✓**	**✓**	**✓**	$\:\times\:$	**✓**
Parametric study on cilia length	$\:\times\:$	$\:\times\:$	$\:\times\:$	$\:\times\:$	$\:\times\:$	**✓**
Buongiorno nanofluid effects	$\:\times\:$	**✓**	**✓**	$\:\times\:$	**✓**	**✓**
Enhanced thermal energy transport model	**✓**	**✓**	**✓**	**✓**	**✓**	**✓**

## Mathematical model

### Geometry of the problem

**Fig. 1:**
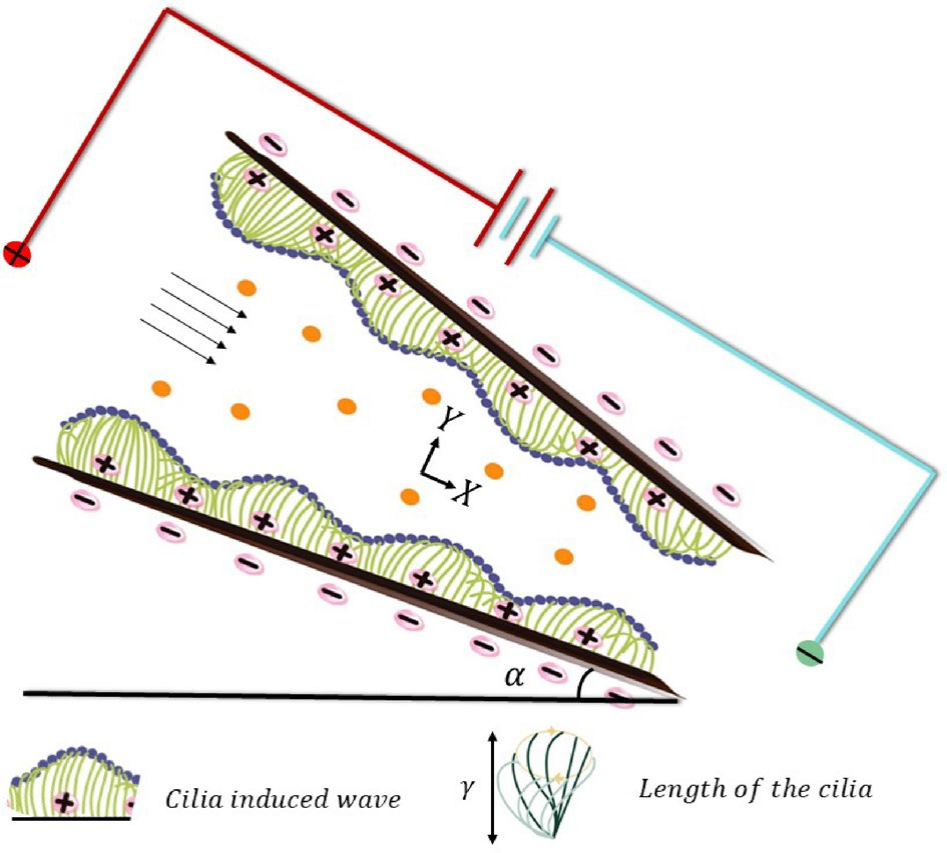
Geometric configuration of an inclined ciliated microchannel under electroosmotic effect.

We examine a symmetric, two-dimensional microchannel with a gradually changing cross-section, which converges in the axial direction and is inclined at an angle $\:\alpha\:$^31^ with respect to the horizontal. A metachronal wave that travels in a streamwise ($\:x)$ direction and propels fluid motion is produced by the coordinated beating of dense arrays of motile cilia that line the channel walls (Fig. 1).

The cilia-tip trajectory is parametrically described in the laboratory frame ($\:{x}^{{\prime\:}}$, $\:{y}^{{\prime\:}}$) in order to mathematically characterize the dynamic deformation caused by ciliary motion. In accordance with the model suggested by Ali et al.^32^, the vertical location of the cilia-tip envelope is depicted as a traveling wave moving in the streamwise direction. Concurrently, the associated horizontal displacement introduces a sinusoidal component that is phase-shifted, leading to an elliptical motion of the cilia tips. More precisely, the position of a cilium tip at any specific point in time is expressed as:

1 $$\:{y}^{{\prime\:}}={H}^{{\prime\:}}={F}^{{\prime\:}}\left({x}^{{\prime\:}},\:{t}^{{\prime\:}}\right)={a}_{1}+{\beta\:}_{0}\left({x}^{{\prime\:}}-c{t}^{{\prime\:}}\right)+\gamma\:{a}^{*}\text{cos}\left(\frac{2\pi\:}{\lambda\:}\left({x}^{{\prime\:}}-c{t}^{{\prime\:}}\right)\right)$$

2 $$\:{x}^{{\prime\:}}={G}^{{\prime\:}}\left({x}^{{\prime\:}},\:{{x}_{0}}^{{\prime\:}}{,\:t}^{{\prime\:}}\right)={{x}_{0}}^{{\prime\:}}+{\beta\:}_{0}\left({x}^{{\prime\:}}-c{t}^{{\prime\:}}\right)+\gamma\:{a}^{*}{\alpha\:}_{0}\text{sin}\left(\frac{2\pi\:}{\lambda\:}\left({x}^{{\prime\:}}-c{t}^{{\prime\:}}\right)\right)$$

Where $\:{a}_{1}$ is the average ciliated tube radius, $\:{\beta\:}_{0}$ is the non-uniformity factor, $\:c$ is the wave speed, $\:\gamma\:$ is the cilia length, $\:{a}^{*}$ is the wave amplitude, $\:{\alpha\:}_{0}$ is the eccentricity of the elliptical orbit, $\:{{x}_{0}}^{{\prime\:}}$ is the position of the reference particle, and $\:\lambda\:$ is the wavelength.

To measure fluid motion caused by beating cilia, the instantaneous cilia tip velocity should be measured. These tip velocities establish the boundary conditions at the ciliated wall and impart momentum to the surrounding fluid. The parametric expressions of the vertical and horizontal components of the cilia-tip envelope, that is, $\:{F}^{{\prime\:}}\left({x}^{{\prime\:}},\:{t}^{{\prime\:}}\right)$ and $\:{G}^{{\prime\:}}\left({x}^{{\prime\:}},\:{{x}_{0}}^{{\prime\:}}{,\:t}^{{\prime\:}}\right)$, are used to define cilia-tip motion in the laboratory frame. Differentiating these functions with respect to time and using the chain rule to account for the coupled dependence between $\:{x}^{{\prime\:}}$ and $\:{t}^{{\prime\:}}$, leads to corresponding velocity components $\:\left({W}^{{\prime\:}},\:{V}^{{\prime\:}}\right)$:

3 $$\:{W}^{{\prime\:}}={\left(\frac{\partial\:{x}^{{\prime\:}}}{\partial\:{t}^{{\prime\:}}}\right)}_{{{x}_{0}}^{{\prime\:}}}=\frac{\partial\:{G}^{{\prime\:}}}{\partial\:{t}^{{\prime\:}}}+\frac{\partial\:{G}^{{\prime\:}}}{\partial\:{x}^{{\prime\:}}}\frac{\partial\:{x}^{{\prime\:}}}{\partial\:{t}^{{\prime\:}}}=\frac{\partial\:{G}^{{\prime\:}}}{\partial\:{t}^{{\prime\:}}}+\frac{\partial\:{G}^{{\prime\:}}}{\partial\:{x}^{{\prime\:}}}{W}^{{\prime\:}}$$

4 $$\:{V}^{{\prime\:}}={\left(\frac{\partial\:{H}^{{\prime\:}}}{\partial\:{t}^{{\prime\:}}}\right)}_{{{x}_{0}}^{{\prime\:}}}=\frac{\partial\:{F}^{{\prime\:}}}{\partial\:{t}^{{\prime\:}}}+\frac{\partial\:{F}^{{\prime\:}}}{\partial\:{x}^{{\prime\:}}}\frac{\partial\:{x}^{{\prime\:}}}{\partial\:{t}^{{\prime\:}}}=\frac{\partial\:{F}^{{\prime\:}}}{\partial\:{t}^{{\prime\:}}}+\frac{\partial\:{F}^{{\prime\:}}}{\partial\:{x}^{{\prime\:}}}{W}^{{\prime\:}}$$

Considering Eqs. (1, 2), Eqs. (3, 4) can be transformed as:

5 $$\:{W}^{{\prime\:}}=\frac{\frac{\partial\:{G}^{{\prime\:}}}{\partial\:{t}^{{\prime\:}}}}{\left(1-\frac{\partial\:{G}^{{\prime\:}}}{\partial\:{x}^{{\prime\:}}}\right)}$$

6 $$\:{V}^{{\prime\:}}=\frac{\frac{\partial\:{F}^{{\prime\:}}}{\partial\:{t}^{{\prime\:}}}\:-\:\left(\frac{\partial\:{F}^{{\prime\:}}}{\partial\:{t}^{{\prime\:}}}\frac{\partial\:{G}^{{\prime\:}}}{\partial\:{x}^{{\prime\:}}}\right)\:+\left(\:\frac{\partial\:{F}^{{\prime\:}}}{\partial\:{x}^{{\prime\:}}}\frac{\partial\:{G}^{{\prime\:}}}{\partial\:{t}^{{\prime\:}}}\right)}{\left(1-\frac{\partial\:{G}^{{\prime\:}}}{\partial\:{x}^{{\prime\:}}}\right)}$$

By substituting the specific forms of the cilia-tip envelope functions $\:{F}^{{\prime\:}}\left({x}^{{\prime\:}},\:{t}^{{\prime\:}}\right)$ and $\:{G}^{{\prime\:}}\left({x}^{{\prime\:}},\:{{x}_{0}}^{{\prime\:}}{,\:t}^{{\prime\:}}\right)$ defined in Eqs. (1), (2), the corresponding partial derivatives $\:\frac{\partial\:{G}^{{\prime\:}}}{\partial\:{t}^{{\prime\:}}}$, $\:\frac{\partial\:{G}^{{\prime\:}}}{\partial\:{x}^{{\prime\:}}}$, $\:\frac{\partial\:{F}^{{\prime\:}}}{\partial\:{t}^{{\prime\:}}}$, and $\:\frac{\partial\:{F}^{{\prime\:}}}{\partial\:{x}^{{\prime\:}}}$ can be obtained. Incorporating these derivatives into Eqs. (5) and (6) yield the explicit expressions for the instantaneous horizontal and vertical tip velocities as given in Eqs. (7) and (8).

7 $$\:{W}^{{\prime\:}}=-\frac{{\beta\:}_{0}c+\gamma\:{a}^{*}{\alpha\:}_{0}c\frac{2\pi\:}{\lambda\:}\text{cos}\left(\frac{2\pi\:}{\lambda\:}\left({x}^{{\prime\:}}-c{t}^{{\prime\:}}\right)\right)}{{1-\beta\:}_{0}-\gamma\:{a}^{*}{\alpha\:}_{0}\frac{2\pi\:}{\lambda\:}\text{cos}\left(\frac{2\pi\:}{\lambda\:}\left({x}^{{\prime\:}}-c{t}^{{\prime\:}}\right)\right)}$$

8 $$\:{V}^{{\prime\:}}=-\frac{{\beta\:}_{0}c-\gamma\:{a}^{*}c\frac{2\pi\:}{\lambda\:}\text{sin}\left(\frac{2\pi\:}{\lambda\:}\left({x}^{{\prime\:}}-c{t}^{{\prime\:}}\right)\right)}{{1-\beta\:}_{0}-\gamma\:{a}^{*}{\alpha\:}_{0}\frac{2\pi\:}{\lambda\:}\text{cos}\left(\frac{2\pi\:}{\lambda\:}\left({x}^{{\prime\:}}-c{t}^{{\prime\:}}\right)\right)}$$

### Mathematical formulation

The Casson model is adopted as it effectively represents biological fluids such as blood and mucus, which exhibit yield stress and shear-thinning characteristics. It captures the transition from solid-like to fluid-like behavior beyond a critical shear rate, ensuring a more realistic description of non-Newtonian microscale flow in the present study. The rheology of an isotropic and incompressible flow of a Casson Fluid^33^ is

9 $$\:{\stackrel{-}{\tau\:}}_{ij}=\left\{\begin{array}{c}2\left(\mu\:\left({T}^{{\prime\:}}\right)+\frac{{\stackrel{-}{P}}_{y}}{\sqrt{2\stackrel{-}{\pi\:}}}\right){\stackrel{-}{e}}_{ij}\:,\:\:\:\:\stackrel{-}{\pi\:}>{\stackrel{-}{\pi\:}}_{c}\\\:2\left(\mu\:\left({T}^{{\prime\:}}\right)+\frac{{\stackrel{-}{P}}_{y}}{\sqrt{2{\stackrel{-}{\pi\:}}_{c}}}\right){\stackrel{-}{e}}_{ij}\:,\:\:\:\stackrel{-}{\pi\:}<{\stackrel{-}{\pi\:}}_{c}\end{array}\right.$$

where, $\:\mu\:\left({T}^{{\prime\:}}\right)$ is the plastic dynamic viscosity varying with respect to temperature, $\:{\stackrel{-}{e}}_{ij}$ indicates the rate of strain component, $\:\:\stackrel{-}{\pi\:}$ is the deformation rate tensor multiplied by itself, i.e., $\:\:\stackrel{-}{\pi\:}={\stackrel{-}{e}}_{ij}{\stackrel{-}{e}}_{ij}$, $\:{\stackrel{-}{\pi\:}}_{c}$ reflects the non-Newtonian model’s critical value for $\:\stackrel{-}{\pi\:}$, $\:{\stackrel{-}{P}}_{y}$ represents the Casson yield stress and is given by $\:{\stackrel{-}{P}}_{y}=\frac{\mu\:\left({T}^{{\prime\:}}\right)\sqrt{2{\stackrel{-}{\pi\:}}_{c}}}{\beta\:}\:$, β is known as the Casson fluid parameter.

The apparent viscosity of the Casson fluid is expressed as

$$\:\mu\:\left({T}^{{\prime\:}}\right)+\frac{{\stackrel{-}{P}}_{y}}{\sqrt{2{\stackrel{-}{\pi\:}}_{c}}}=\mu\:\left({T}^{{\prime\:}}\right)\left(1+\frac{1}{\beta\:}\right),\:\text{w}\text{h}\text{e}\text{n}\:\stackrel{-}{\pi\:}<{\stackrel{-}{\pi\:}}_{c}.$$

Then, the constitutive equation of Casson fluid is expressed as

$$\:\stackrel{-}{\tau\:}=2\mu\:\left({T}^{{\prime\:}}\right)\left(1+\frac{1}{\beta\:}\right){\stackrel{-}{e}}_{ij}\:,\:\text{w}\text{h}\text{e}\text{r}\text{e},\:{\stackrel{-}{e}}_{ij}=\frac{1}{2}\left(\frac{\partial\:{{u}^{{\prime\:}}}_{i}}{\partial\:{{x}^{{\prime\:}}}_{j}}+\frac{\partial\:{{u}^{{\prime\:}}}_{j}}{\partial\:{{x}^{{\prime\:}}}_{i}}\right).$$

As the Casson parameter $\:\beta\:$ approaches infinity, the yield stress contribution diminishes, and the constitutive relationship converges to that of a Newtonian fluid with viscosity $\:\mu\:\left({T}^{{\prime\:}}\right)$.

The governing equations for the conservation of mass^34^, momentum^35^, energy^36^, and solute concentration^37^ are presented below.

10 $$\:\frac{\partial\:{w}^{{\prime\:}}}{\partial\:{x}^{{\prime\:}}}+\frac{\partial\:{v}^{{\prime\:}}}{\partial\:{y}^{{\prime\:}}}=0$$

11 $$\begin{aligned} \rho \left( {\frac{{\partial w^{\prime}}}{{\partial t^{\prime}}} + w^{\prime}\frac{{\partial w^{\prime}}}{{\partial x^{\prime}}} + v^{\prime}\frac{{\partial w^{\prime}}}{{\partial y^{\prime}}}} \right) & = - \frac{{\partial p^{\prime}}}{{\partial x^{\prime}}} + \frac{{\partial \tau ^{\prime}_{{x^{\prime}x^{\prime}}} }}{{\partial x^{\prime}}} + \frac{{\partial \tau ^{\prime}_{{x^{\prime}y^{\prime}}} }}{{\partial y^{\prime}}} + \left( {1 - C_{0} } \right)\rho _{f} g\beta _{T} \left( {T^{\prime} - T_{0} } \right) \\ & + \left( {\rho _{c} - \rho _{f} } \right)g\beta _{C} \left( {C^{\prime} - C_{0} } \right) + \rho g\sin \alpha + \rho _{e} E_{x} \\ \end{aligned}$$

12 $$\:\rho\:\left(\frac{\partial\:{v}^{{\prime\:}}}{\partial\:{t}^{{\prime\:}}}+{w}^{{\prime\:}}\frac{\partial\:{v}^{{\prime\:}}}{\partial\:{x}^{{\prime\:}}}+{v}^{{\prime\:}}\frac{\partial\:{v}^{{\prime\:}}}{\partial\:{y}^{{\prime\:}}}\right)=-\frac{\partial\:{p}^{{\prime\:}}}{\partial\:{y}^{{\prime\:}}}+\frac{\partial\:{{\tau\:}^{{\prime\:}}}_{{x}^{{\prime\:}}{y}^{{\prime\:}}}}{\partial\:{x}^{{\prime\:}}}+\frac{\partial\:{{\tau\:}^{{\prime\:}}}_{{y}^{{\prime\:}}{y}^{{\prime\:}}}}{\partial\:{y}^{{\prime\:}}}-\rho\:g\text{cos}\alpha\:+{\rho\:}_{e}{E}_{y}$$

13 $$\begin{aligned} \rho c_{p} \left( {\frac{{\partial T^{\prime}}}{{\partial t^{\prime}}} + w^{\prime}\frac{{\partial T^{\prime}}}{{\partial x^{\prime}}} + v^{\prime}\frac{{\partial T^{\prime}}}{{\partial y^{\prime}}}} \right) & = \frac{\partial }{{\partial x^{\prime}}}\left( {\bar{K}\left( {T^{\prime}} \right)\frac{{\partial T^{\prime}}}{{\partial x^{\prime}}}} \right) + \frac{\partial }{{\partial y^{\prime}}}\left( {\bar{K}\left( {T^{\prime}} \right)\frac{{\partial T^{\prime}}}{{\partial y^{\prime}}}} \right) \\ & + \tau ^{\prime}_{{x^{\prime}x^{\prime}}} \frac{{\partial w^{\prime}}}{{\partial x^{\prime}}} + \tau ^{\prime}_{{x^{\prime}y^{\prime}}} \left( {\frac{{\partial v^{\prime}}}{{\partial x^{\prime}}} + \frac{{\partial w^{\prime}}}{{\partial y^{\prime}}}} \right) + \tau ^{\prime}_{{y^{\prime}y^{\prime}}} \frac{{\partial v^{\prime}}}{{\partial y^{\prime}}} \\ & + \rho _{f} c_{f} \left( \begin{gathered} D_{B} \left( {\frac{{\partial C^{\prime}}}{{\partial x^{\prime}}}\frac{{\partial T^{\prime}}}{{\partial x^{\prime}}} + \frac{{\partial C^{\prime}}}{{\partial y^{\prime}}}\frac{{\partial T^{\prime}}}{{\partial y^{\prime}}}} \right) \hfill \\ + \frac{{D_{T} }}{{T_{b} }}\left( {\frac{{\partial T^{\prime}}}{{\partial x^{\prime}}} + \frac{{\partial T^{\prime}}}{{\partial y^{\prime}}}} \right)^{2} \hfill \\ \end{gathered} \right). \\ \end{aligned}$$

14 $$\:\frac{\partial\:{C}^{{\prime\:}}}{\partial\:{t}^{{\prime\:}}}+{w}^{{\prime\:}}\frac{\partial\:{C}^{{\prime\:}}}{\partial\:{x}^{{\prime\:}}}+{v}^{{\prime\:}}\frac{\partial\:{C}^{{\prime\:}}}{\partial\:{y}^{{\prime\:}}}={D}_{B}\left(\frac{{\partial\:}^{2}{C}^{{\prime\:}}}{\partial\:{{x}^{{\prime\:}}}^{2}}+\frac{{\partial\:}^{2}{C}^{{\prime\:}}}{\partial\:{{y}^{{\prime\:}}}^{2}}\right)+\frac{{D}_{T}}{{T}_{b}}\left(\frac{{\partial\:}^{2}{T}^{{\prime\:}}}{\partial\:{{x}^{{\prime\:}}}^{2}}+\frac{{\partial\:}^{2}{T}^{{\prime\:}}}{\partial\:{{y}^{{\prime\:}}}^{2}}\right)$$

The extra stress tensor components are:

15 $$\:{{\tau\:}^{{\prime\:}}}_{{x}^{{\prime\:}}{x}^{{\prime\:}}}=2\stackrel{-}{\mu\:}\left({T}^{{\prime\:}}\right)\left(1+\frac{1}{\beta\:}\right)\frac{\partial\:{w}^{{\prime\:}}}{\partial\:{x}^{{\prime\:}}}$$

16 $$\:{{\tau\:}^{{\prime\:}}}_{{y}^{{\prime\:}}{y}^{{\prime\:}}}=2\stackrel{-}{\mu\:}\left({T}^{{\prime\:}}\right)\left(1+\frac{1}{\beta\:}\right)\frac{\partial\:{v}^{{\prime\:}}}{\partial\:{y}^{{\prime\:}}}$$

17 $$\:{{\tau\:}^{{\prime\:}}}_{{x}^{{\prime\:}}{y}^{{\prime\:}}}=\stackrel{-}{\mu\:}\left({T}^{{\prime\:}}\right)\left(1+\frac{1}{\beta\:}\right)\left(\frac{\partial\:{w}^{{\prime\:}}}{\partial\:{y}^{{\prime\:}}}+\frac{\partial\:{v}^{{\prime\:}}}{\partial\:{x}^{{\prime\:}}}\right).$$

The Poisson-Boltzmann equation describes the electrical potential distribution in an electrolytic solution by considering direct electrostatic interactions. According to this theory, the electrical potential $\:\stackrel{-}{\phi\:}$ within the electrical double layers is given as:

18 $$\:{\nabla\:}^{2}\stackrel{-}{\phi\:}=-\frac{{\rho\:}_{e}}{{\epsilon\:}_{0}}$$

where $\:{\epsilon\:}_{0}$​ represents the dielectric permittivity of the medium, and $\:{\rho\:}_{e}$ denotes the total charge density per unit volume. The charge density is expressed as:

19 $$\:{\rho\:}_{e}=e\stackrel{-}{z}({n}_{+}-{n}_{-})$$

where $\:e\left(1.6*{10}^{-19}C\right)$ is the elementary charge, $\:\stackrel{-}{z}$ represents the valency of both positive and negative ions, and $\:{n}_{+}\:and\:{n}_{-}$​ indicate the number densities of cations and anions.

Applying the Boltzmann distribution, the number densities of positive and negative ions can be given by:

20 $$\:{n}_{\pm\:}={n}_{0}{e}^{\mp\:\frac{e\stackrel{-}{z}\stackrel{-}{\phi\:}}{{K}_{B}{T}_{a}}}$$

where $\:{n}_{0}$​ is the reference ion concentration, $\:{K}_{B}$​ is the Boltzmann constant, and $\:{T}_{a}$​ represents the average temperature of the electrolyte solution.

The charge density per unit volume is given by:

21 $$\:{\rho\:}_{e}=-2{n}_{0}e\stackrel{-}{z}\text{sinh}\frac{e\stackrel{-}{z}\stackrel{-}{\phi\:}}{{K}_{B}{T}_{a}}$$

Substituting this result into Eq. (18), the Poisson-Boltzmann equation can be rewritten as:

22 $$\:{\nabla\:}^{2}\stackrel{-}{\phi\:}=\frac{2{n}_{0}e\stackrel{-}{z}}{{\epsilon\:}_{0}}\text{sinh}\frac{e\stackrel{-}{z}\stackrel{-}{\phi\:}}{{K}_{B}{T}_{a}}$$

Here, we assume that the electrical potential energy is smaller than the thermal energy, i.e., $\:\left|e\stackrel{-}{z}\stackrel{-}{\phi\:}\right|\le\:\left|{K}_{B}{T}_{a}\right|$ then the Debye–Hückel approximation $\:\left(\text{sinh}\frac{e\stackrel{-}{z}\stackrel{-}{\phi\:}}{{K}_{B}{T}_{a}}\approx\:\frac{e\stackrel{-}{z}\stackrel{-}{\phi\:}}{{K}_{B}{T}_{a}}\right)$ can be imposed to linearize the Poisson–Boltzmann equation^38^. This approximation is physically valid when the zeta potential is relatively small, as is typically the case in electroosmotic microflows. Under such low-potential conditions, the potential distribution across the electric double layer remains nearly linear, allowing accurate analytical treatment without significant deviation from the complete nonlinear solution.

23 $$\:{\nabla\:}^{2}\stackrel{-}{\phi\:}=\frac{2{n}_{0}{e}^{2}{\stackrel{-}{z}}^{2}\stackrel{-}{\phi\:}}{{\epsilon\:}_{0}{K}_{B}{T}_{a}}$$

The no-slip of the fluid-cilia interface is the traditional boundary condition used in this model of a microchannel with wall-anchored cilia. This means that the fluid velocity would be equal to the velocity on the surface of the cilia, creating no relative movement between the fluid and the cilia surface. The continuum hypothesis supports it and is often applied to microfluidic models that include ciliated geometries^39,40^. The corresponding boundary conditions in the Cartesian reference frame (fixed frame) are

24 $$\:\begin{array}{c}\frac{\partial\:{w}^{{\prime\:}}}{\partial\:{y}^{{\prime\:}}}=0\:,\:\:\frac{\partial\:{T}^{{\prime\:}}}{\partial\:{y}^{{\prime\:}}}=0\:,\:\:\frac{\partial\:{C}^{{\prime\:}}}{\partial\:{y}^{{\prime\:}}}=0\:\:at\:{y}^{{\prime\:}}=0,\:\:\:\:\:\:\:\:\:\:\:\:\:\:\:\:\:\:\:\:\:\:\:\:\:\:\:\:\:\:\:\:\:\:\:\:\:\:\:\:\:\:\:\:\:\:\:\:\:\:\:\:\\\:{w}^{{\prime\:}}=-\frac{{\beta\:}_{0}c+\gamma\:{a}^{*}{\alpha\:}_{0}c\frac{2\pi\:}{\lambda\:}\text{cos}\left(\frac{2\pi\:}{\lambda\:}\left({x}^{{\prime\:}}-c{t}^{{\prime\:}}\right)\right)}{{1-\beta\:}_{0}-\gamma\:{a}^{*}{\alpha\:}_{0}\frac{2\pi\:}{\lambda\:}\text{cos}\left(\frac{2\pi\:}{\lambda\:}\left({x}^{{\prime\:}}-c{t}^{{\prime\:}}\right)\right)}\:\:,\:\:\:{T}^{{\prime\:}}=\:{T}_{0},\:{C}^{{\prime\:}}=\:{C}_{0}\:\:at\:{y}^{{\prime\:}}={H}^{{\prime\:}}.\end{array}$$

The dynamic viscosity and thermal conductivity of the Casson nanofluid are presumed to change with temperature, adhering to conventional exponential laws^41^. In particular, the viscosity diminishes while the thermal conductivity enhances as temperature rises, and they are expressed as follows:

25 $$\:\stackrel{-}{\mu\:}\left({T}^{{\prime\:}}\right)={\mu\:}_{0}{e}^{-{\varphi\:}_{1}\left({T}^{{\prime\:}}-{T}_{0}\right)}$$

26 $$\:\stackrel{-}{K}\left({T}^{{\prime\:}}\right)={K}_{0}{e}^{{\varphi\:}_{2}\left({T}^{{\prime\:}}-{T}_{0}\right)}$$

where $\:{\mu\:}_{0}$ and $\:{\varphi\:}_{1}$ are respectively the viscosity of fluid at constant temperature and the dimensional viscosity parameter. $\:{\varphi\:}_{1}$=0 is the case for constant viscosity, $\:{K}_{0}$ is the thermal conductivity, $\:{\varphi\:}_{2}$ is the temperature coefficient of thermal conductivity. These constants regulate how sensitive the fluid properties are to temperature and usually align with the condition 0 < $\:{\varphi\:}_{1}$, $\:{\varphi\:}_{2}$ ≪ 1 for liquids. In the case of gases, negative values for $\:{\varphi\:}_{1}$, and $\:{\varphi\:}_{2}$ have been observed, suggesting an inverse behavior^41^.

For small values $\:{\varphi\:}_{1}\left({T}^{{\prime\:}}-{T}_{0}\right)$ and $\:{\varphi\:}_{2}\left({T}^{{\prime\:}}-{T}_{0}\right)$, the exponential functions for viscosity and thermal conductivity can be simplified using their first-order Taylor series expansions.

27 $$\:\stackrel{-}{\mu\:}\left({T}^{{\prime\:}}\right)={\mu\:}_{0}(1-{\varphi\:}_{1}\left({T}^{{\prime\:}}-{T}_{0}\right)$$

28 $$\:\stackrel{-}{K}\left({T}^{{\prime\:}}\right)={K}_{0}(1+{\varphi\:}_{2}\left({T}^{{\prime\:}}-{T}_{0}\right)$$

The governing equations are initially unsteady within the laboratory (fixed) frame of reference. To streamline the analysis, a coordinate transformation is employed to shift from the lab frame to a wave frame that travels with the wave velocity $\:c$. This transformation renders the governing equations steady in the moving frame, thereby facilitating analytical or numerical treatment. The transformation is generally defined as:

29 $$\:\stackrel{-}{x}={x}^{{\prime\:}}-c{t}^{{\prime\:}},\:\:\stackrel{-}{y}={y}^{{\prime\:}},\:\:\stackrel{-}{w}={w}^{{\prime\:}}-c,\:\:\stackrel{-}{v}={v}^{{\prime\:}},\:\:\stackrel{-}{p}\left(\stackrel{-}{x}\right)={p}^{{\prime\:}}\left({x}^{{\prime\:}},\:{t}^{{\prime\:}}\right),\:\:\stackrel{-}{h}\left(\stackrel{-}{x},\:\stackrel{-}{t}\right)=\:{H}^{{\prime\:}}\left({x}^{{\prime\:}},\:{t}^{{\prime\:}}\right).$$

Now, introducing the following non-dimensional variables:

30 $$\begin{gathered} x = \frac{{\bar{x}}}{\lambda }~,~y = \frac{{\bar{y}}}{{a_{1} }}~,~w = \frac{{\bar{w}}}{c}~,~v = \frac{{\bar{v}}}{{c\delta }}~,~t = \frac{{ct^{\prime}}}{\lambda }~,~ \hfill \\ h = \frac{{\bar{h}}}{{a_{1} }}~,~\tau _{{ij}} = \frac{{a_{1} \tau ^{\prime}_{{ij}} }}{{\mu _{0} c}}~,~\delta = \frac{{a_{1} }}{\lambda }~,~\text{Re} = \frac{{\rho ca_{1} }}{{\mu _{0} }}~, \hfill \\ ~F = \frac{{c\mu _{0} }}{{\rho ga_{1} ^{2} }}~,~U_{{hs}} = \frac{{ - \varepsilon _{0} \phi _{0} E_{x} }}{{c\mu _{0} }}~,~\phi = \frac{{\bar{\phi }}}{{\phi _{0} }}~,~ \hfill \\ \phi _{0} = \frac{{K_{B} T_{a} }}{{e\tilde{z}}}~,~F = \frac{{Fr}}{{\text{Re} }}~,~Fr = \frac{{c^{2} }}{{ga_{1} }}~,~Br = \frac{{\mu _{0} c^{2} }}{{T_{0} K_{0} }}~,~ \hfill \\ K\left( \theta \right) = \frac{{\bar{K}\left( {T^{\prime}} \right)}}{{K_{0} }}~,~\mu \left( \theta \right) = \frac{{\bar{\mu }\left( {T^{\prime}} \right)}}{{\mu _{0} }}~,~\theta = \frac{{T^{\prime} - T_{0} }}{{T_{1} - T_{0} }}~,~ \hfill \\ P = \frac{{a_{1} ^{2} p^{\prime}}}{{c\lambda \mu _{0} }}~,~\kappa = a_{1} e\bar{z}\sqrt {\frac{{2n_{0} }}{{\varepsilon _{0} K_{B} T_{a} }}} = \frac{{a_{1} }}{{\lambda _{D} }}, \hfill \\ b_{1} = \frac{{a^{*} }}{{a_{1} }}~,~\,Nb~ = \frac{{\left( {\rho c} \right)_{f} D_{B} (C_{1} - C_{0} )}}{{K_{0} }}~,~ \hfill \\ K_{{nu}} = \frac{{\lambda \beta _{0} }}{{a_{1} }}~,~\xi = \frac{{C^{\prime} - C_{0} }}{{C_{1} - C_{0} }},\,Nt = \frac{{\left( {\rho c} \right)_{f} D_{T} \left( {T_{1} - T_{0} } \right)}}{{K_{0} T_{b} }}~, \hfill \\ Gr = \frac{{\left( {1 - C_{0} } \right)\rho _{f} g\beta _{t} \left( {T_{1} - T_{0} } \right)a_{1} ^{2} }}{{c\mu _{0} }}, \hfill \\ Gm = \frac{{\left( {\rho _{c} - \rho _{f} } \right)~g\beta _{c} \left( {C_{1} - C_{0} } \right)a_{1} ^{2} }}{{c\mu _{0} }}, \hfill \\ \phi _{1} = \varphi _{1} \left( {T^{\prime} - T_{0} } \right),\,\phi _{2} = \varphi _{2} \left( {T^{\prime} - T_{0} } \right) \hfill \\ \end{gathered}$$

The lubrication theory is used to simplify the governing equations. This is a physically justified approximation to cilia-driven flows, where the wavelength of the wall deformation is large compared to the channel height and the Reynolds number is small, allowing viscous forces to dominate over inertial forces. This is where the leading-order terms can be included, and the higher-order terms are rejected in $\:\delta\:$. With this assumption, along with the coordinate transformation given in Eq. (29) and the use of dimensionless variables as expressed in Eq. (30), the governing Eqs. (10–14) are significantly simplified.

31 $$\:\frac{\partial\:w}{\partial\:x}+\frac{\partial\:v}{\partial\:y}=0$$

32 $$\:\frac{\partial\:p}{\partial\:x}=\left(1+\frac{1}{\beta\:}\right)\frac{\partial\:}{\partial\:y}\left(\mu\:\left(\theta\:\right)\frac{\partial\:w}{\partial\:y}\right)+Gr\theta\:+Gm\xi\:+\frac{{sin}\alpha\:}{F}+{\kappa\:}^{2}\phi\:{U}_{hs}$$

33 $$\:\frac{\partial\:p}{\partial\:y}=0$$

34 $$\:\frac{\partial\:}{\partial\:y}\left(K\left(\theta\:\right)\frac{\partial\:\theta\:}{\partial\:y}\right)+Br\:\mu\:\left(\theta\:\right)\left(1+\frac{1}{\beta\:}\right){\left(\frac{\partial\:w}{\partial\:y}\right)}^{2}+Nb\frac{\partial\:\xi\:}{\partial\:y}\frac{\partial\:\theta\:}{\partial\:y}+Nt{\left(\frac{\partial\:\theta\:}{\partial\:y}\right)}^{2}=0$$

35 $$\:\frac{{\partial\:}^{2}\xi\:}{\partial\:{y}^{2}}+\:\frac{Nt}{Nb}\:\frac{{\partial\:}^{2}\theta\:}{\partial\:{y}^{2}}=0.$$

The associated non-dimensional boundary conditions are as follows:

36 $$\begin{gathered} \frac{{\partial w}}{{\partial y}} = 0~,~\frac{{\partial \theta }}{{\partial y}} = 0~,~\frac{{\partial \xi }}{{\partial y}} = 0~,~at~y = 0, \hfill \\ ~w = - \frac{1}{{1 - K_{{nu}} \delta - \delta \gamma b_{1} \alpha _{0} 2\pi \cos (2\pi x)}}~~,~\theta = 1,\xi = 1~,~ \hfill \\ at~y = h\left( x \right) = 1 + K_{{nu}} x + \gamma b_{1} \cos (2\pi x). \hfill \\ \end{gathered}$$

The Eqs. (27, 28) are transformed into:

37 $$\:\:\mu\:\left(\theta\:\right)=\left(1-{\phi\:}_{1}\theta\:\right)$$

38 $$\:K\left(\theta\:\right)=\left(1+{\phi\:}_{2}\theta\:\right)$$

Furthermore, imposing the boundary conditions $\:{\left.\frac{\partial\:\phi\:}{\partial\:y}\right|}_{y=0}=0\:and\:{\left.\phi\:\right|}_{y=h}=1,\:$ the potential function is obtained as:

39 $$\:\phi\:=\frac{\text{cosh}\left(\kappa\:y\right)}{\text{cosh}\left(\kappa\:h\right)}$$

## Solution of the problem using the homotopy perturbation method (HPM)

**Fig. 2:**
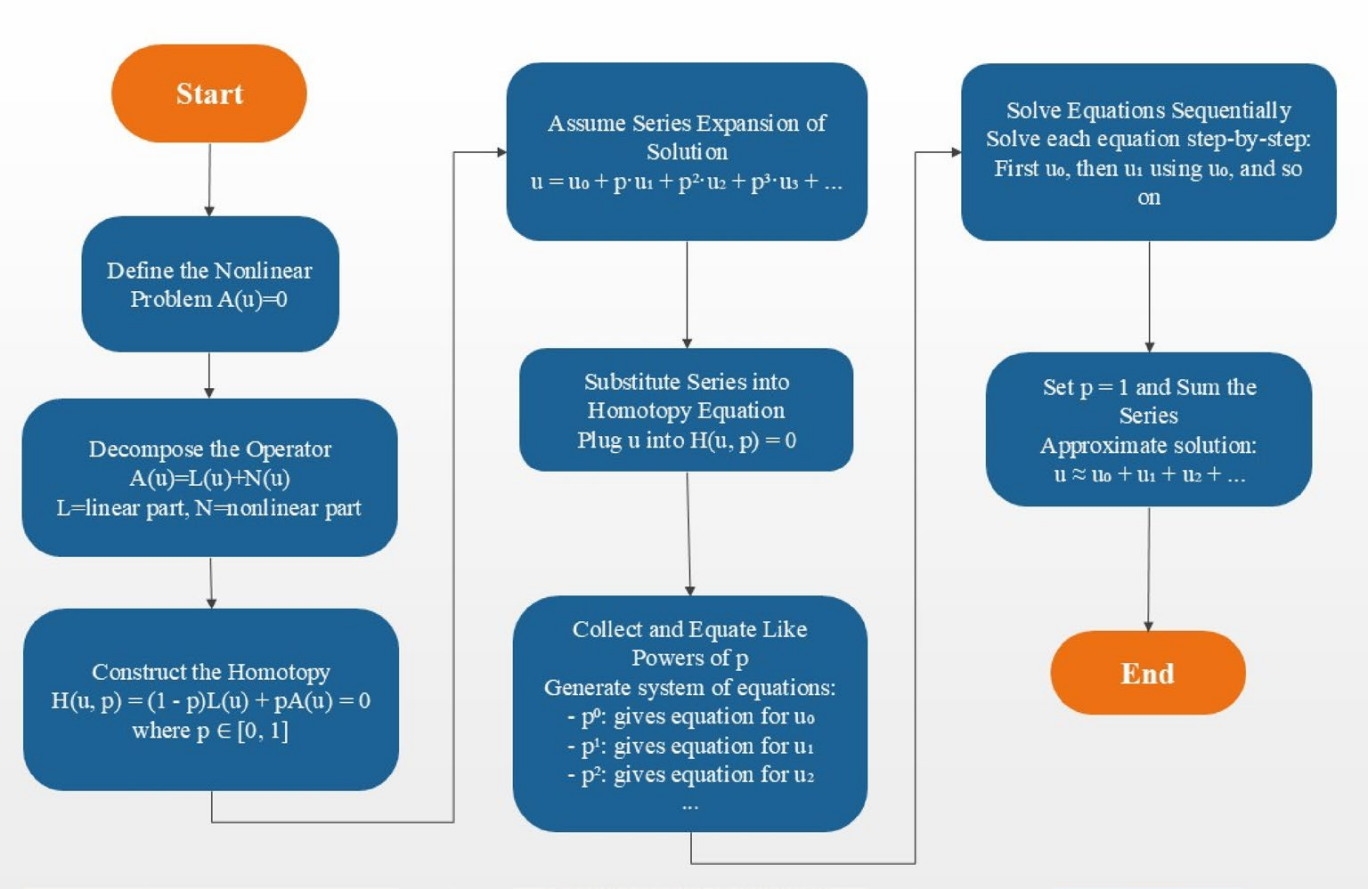
Schematic representation of the Homotopy Perturbation Method (HPM) steps used to solve the nonlinear coupled equations.

This section will focus on evaluating the solutions of the non-dimensional Eqs. (32–35) and remain restricted to the boundary conditions of the Eq. (36). These non-linear equations, which are interconnected, cannot be solved using analytical methods. Thus, the homotopy perturbation method is employed to obtain convergent series solutions of these equations. The HPM is a reliable method for analyzing both linear and nonlinear differential equations across various fields. It is based on topological and classical perturbation theories. Figure 2 illustrates the overall implementation procedures of HPM that were used in this research.

The convergence behavior of the HPM solution was examined by evaluating the magnitude of successive correction terms, which showed a rapid reduction, confirming stable convergence. The series was truncated after the second-order term since higher-order contributions were found to have an insignificant influence on the flow, temperature, and concentration fields. Thus, the second-order approximation provides sufficient accuracy while maintaining analytical simplicity. The primary non-dimensional Eqs. (32–35) are reformulated below, following the substitution of Eqs. (37–39)

40 $$\:\frac{\partial\:p}{\partial\:x}=\left(1+\frac{1}{\beta\:}\right)\left(\frac{\partial\:}{\partial\:y}\left(1-{\phi\:}_{1}\theta\:\right)\frac{\partial\:w}{\partial\:y}\right)+Gr\theta\:+Gm\xi\:+\frac{{sin}\alpha\:}{F}+{\kappa\:}^{2}\frac{\text{cosh}\left(\kappa\:y\right)}{\text{cosh}\left(\kappa\:h\right)}{U}_{hs}=0$$

41 $$\:\left(\frac{\partial\:}{\partial\:y}\left(1+{\phi\:}_{2}\theta\:\right)\frac{\partial\:\theta\:}{\partial\:y}\right)+Br\left(1-{\phi\:}_{1}\theta\:\right)\left(1+\frac{1}{\beta\:}\right){\left(\frac{\partial\:w}{\partial\:y}\right)}^{2}+{N}_{b}\frac{\partial\:\xi\:}{\partial\:y}\frac{\partial\:\theta\:}{\partial\:y}+{N}_{t}{\left(\frac{\partial\:\theta\:}{\partial\:y}\right)}^{2}=0$$

42 $$\:\frac{{\partial\:}^{2}\xi\:}{\partial\:{y}^{2}}+\:\frac{{N}_{t}}{{N}_{b}}\:\frac{{\partial\:}^{2}\theta\:}{\partial\:{y}^{2}}=0\:.$$

By simplifying the above equations, we get

43 $$\:\frac{{\partial\:}^{2}w}{\partial\:{y}^{2}}-{\phi\:}_{1}\left(\frac{\partial\:\theta\:}{\partial\:y}\frac{\partial\:w}{\partial\:y}+\theta\:\frac{{\partial\:}^{2}w}{\partial\:{y}^{2}}\right)+{a}_{5}\theta\:+{a}_{6}\xi\:+{a}_{7}+{a}_{8}\text{cosh}\left(\kappa\:y\right)=0$$

44 $$\:\frac{{\partial\:}^{2}\theta\:}{\partial\:{y}^{2}}+{\phi\:}_{2}\left({\left(\frac{\partial\:\theta\:}{\partial\:y}\right)}^{2}+\theta\:\frac{{\partial\:}^{2}\theta\:}{\partial\:{y}^{2}}\right)+{a}_{15}\left(1-{\phi\:}_{1}\theta\:\right){\left(\frac{\partial\:w}{\partial\:y}\right)}^{2}+{N}_{b}\frac{\partial\:\xi\:}{\partial\:y}\frac{\partial\:\theta\:}{\partial\:y}+{N}_{t}{\left(\frac{\partial\:\theta\:}{\partial\:y}\right)}^{2}=0$$

45 $$\:\frac{{\partial\:}^{2}\xi\:}{\partial\:{y}^{2}}+\:{a}_{20}\:\frac{{\partial\:}^{2}\theta\:}{\partial\:{y}^{2}}=0\:.$$

Where, $\:{a}_{5}$, $\:{a}_{6},\:{a}_{7},\:{a}_{8},\:{a}_{15},\:{a}_{20}$ are provided in the Appendix.

Consider the general expression of the nonlinear differential equation as:

46 $$\:A\left(u\right)=0$$

where $\:A$ is a differential operator that can be divided into:

47 $$\:A\left(u\right)=L\left(u\right)+N\left(u\right)$$

with $\:L$ denoting the linear component and $\:N$ the nonlinear component of the operator.

A homotopy $\:H(u,q)$ is then constructed as:

48 $$\:H\left(u,q\right)=\left(1-q\right)\left[L\left(u\right)-L\left({u}_{0}\right)\right]+q\left[A\left(u\right)\right]=0$$

where, $\:q\in\:\left[\text{0,1}\right]$ represents an embedding parameter, $\:{u}_{0}$ is an initial approximation that satisfies the boundary conditions.

The homotopy relationships for Eqs. (43–45) are outlined below for this aim:

49 $$\:H\left(w,q\right)=L\left(w\right)-L\left({w}_{0}\right)+qL\left({w}_{0}\right)+q\left[-{\phi\:}_{1}\left(\frac{\partial\:\theta\:}{\partial\:y}\frac{\partial\:w}{\partial\:y}+\theta\:\frac{{\partial\:}^{2}w}{\partial\:{y}^{2}}\right)+{a}_{5}\theta\:+{a}_{6}\xi\:+{a}_{7}+{a}_{8}\text{cosh}\left(\kappa\:y\right)\right]=0$$

50 $$H\left( {\theta ,q} \right) = L\left( \theta \right) - L\left( {\theta _{0} } \right) + qL\left( {\theta _{0} } \right) + q\left[ {\phi _{2} \left( {\left( {\frac{{\partial \theta }}{{\partial y}}} \right)^{2} + \theta \frac{{\partial ^{2} \theta }}{{\partial y^{2} }}} \right) + a_{{15}} \left( {1 - \phi _{1} \theta } \right)\left( {\frac{{\partial w}}{{\partial y}}} \right)^{2} + N_{b} \frac{{\partial \xi }}{{\partial y}}\frac{{\partial \theta }}{{\partial y}} + N_{t} \left( {\frac{{\partial \theta }}{{\partial y}}} \right)^{2} } \right] = 0$$

51 $$\:H\left(\xi\:,q\right)=L\left(\xi\:\right)-L\left({\xi\:}_{0}\right)+qL\left({\xi\:}_{0}\right)+q\left[{a}_{20}\:\frac{{\partial\:}^{2}\theta\:}{\partial\:{y}^{2}}\right]=0$$

Where, $$\:L=\frac{{\partial\:}^{2}}{\partial\:{y}^{2}}\:.$$

In this perturbing methodology, the infinite series for $\:w$, $\:\theta\:$, and $\:\xi\:$ are supposed:

52 $$\begin{gathered} w\left( {x,y} \right) = w_{0} + qw_{1} + q^{2} w_{2} + \ldots \hfill \\ \theta \left( {x,y} \right) = \theta _{0} + q\theta _{1} + q^{2} \theta _{2} + \ldots \hfill \\ \xi \left( {x,y} \right) = \xi _{0} + q\xi _{1} + q^{2} \xi _{2} + \ldots \hfill \\ \end{gathered}$$

By substituting Eq. (52) into (49–51) and aligning the coefficients of like powers of $\:q$, we can establish a system of linear correlations. The velocity, temperature, and concentration fields can be determined by solving these coupled linear equations and substituting the solutions (^$\:{w}_{0},\:{w}_{1},\:{w}_{2},\dots\:$, $\:{\theta\:}_{0},\:{\theta\:}_{1},{\theta\:}_{2},\dots\:$, $\:{\xi\:}_{0},\:{\xi\:}_{1},\:{\xi\:}_{2},\dots\:$) into Eq. (52) by setting $\:q=1$. The velocity, temperature, and concentration fields are expressed as follows:

53 $$\begin{aligned} ~w & = a_{{11}} + a_{{13}} + a_{2} + a_{{27}} + a_{9} - \frac{{y^{2} }}{2} + a_{{12}} y^{2} + a_{{25}} y^{2} \\ & + a_{{10}} y^{4} + a_{{24}} y^{4} + a_{{23}} y^{6} + a_{{22}} y^{8} - a_{{14}} \cosh \left( {y\kappa } \right) \\ & + a_{{28}} \cosh \left( {y\kappa } \right) + a_{{26}} y^{2} \cosh \left( {y\kappa } \right) + a_{{29}} y\sinh \left( {y\kappa } \right) \\ \end{aligned}$$

54 $$\begin{aligned} \theta & = ~a_{{19}} + a_{{36}} + a_{{18}} y^{2} + a_{{34}} y^{2} + a_{{17}} y^{4} + a_{{33}} y^{4} \\ & + a_{{16}} y^{6} + a_{{32}} y^{6} + a_{{31}} y^{8} + a_{{30}} y^{{10}} + \frac{1}{2}\left( {2 - h^{2} + y^{2} } \right) \\ & + a_{{37}} \cosh \left( {y\kappa } \right) + a_{{35}} y^{2} \cosh \left( {y\kappa } \right) \\ & + a_{{39}} y\sinh \left( {y\kappa } \right) + a_{{38}} y^{3} \sinh \left( {y\kappa } \right) \\ \end{aligned}$$

55 $$\:\xi\:\:=\:{a}_{21}+{a}_{4}+{a}_{40}-\frac{{y}^{2}}{2}-{a}_{18}{a}_{20}{y}^{2}-{a}_{21}{y}^{2}-{a}_{17}{a}_{20}{y}^{4}-{a}_{16}{a}_{20}{y}^{6}\:.$$

By integrating the axial velocity over the channel width, the volumetric flow rate is calculated and found to be^42^

56 $$\:Q=\underset{0}{\overset{h}{\int\:}}wdy$$

Using Eq. (53) in Eq. (56), we arrive at

57 $$\begin{aligned} Q & = a_{{13}} h + a_{2} h + a_{9} h - \frac{{h^{3} }}{2} + \frac{1}{3}\left( {a_{{21}} + a_{4} } \right)a_{6} h^{3} + \frac{{a_{{10}} h^{5} }}{5} \\ & + \frac{1}{{315}}h^{5} ( - 21a_{{21}} a_{6} + 45a_{{23}} h^{2} + 35a_{{22}} h^{4} ) - \frac{4}{5}a_{{10}} h^{5} \phi _{1} \\ & + \frac{1}{5}a_{{18}} h^{5} \phi _{1} + \frac{1}{{30}}\left( {2a_{{10}} + 5a_{{17}} } \right)h^{7} \phi _{1} + \frac{1}{8}a_{{16}} h^{9} \phi _{1} \\ & - \frac{1}{{15}}a_{4} a_{6} h^{3} \left( { - 5 + h^{2} } \right)\phi _{1} + \frac{{a_{{19}} h^{3} \phi _{1} }}{{3\left( {1 + \beta } \right)}} + \frac{{h^{5} \phi _{1} }}{{40\left( {1 + \beta } \right)}} \\ & + \frac{{a_{{19}} h^{3} \beta \phi _{1} }}{{3\left( {1 + \beta } \right)}} + \frac{{h^{5} \beta \phi _{1} }}{{40\left( {1 + \beta } \right)}} + \frac{1}{{30}}h^{3} \left( { - 5 + h^{2} } \right)( - 2 + h^{2} )\phi _{1} ^{2} \\ & + \frac{{\partial p}}{{\partial x}}\left( { - \frac{{h^{3} \beta }}{{3\left( {1 + \beta } \right)}} - \frac{{h^{3} \beta \phi _{1} }}{{3\left( {1 + \beta } \right)}} + \frac{{h^{5} \beta \phi _{1} }}{{15\left( {1 + \beta } \right)}}} \right) \\ & + \frac{1}{{840}}a_{5} h^{3} (280a_{{19}} + 15a_{{16}} h^{6} + 14h^{2} (5 + 4a_{{18}} - 14\phi _{1} ) \\ & + 28h^{4} \left( {a_{{17}} + \phi _{1} } \right) + 140\left( { - 1 + 2\phi _{1} } \right)) \\ & + \frac{{h\left( { - 2a_{{26}} + a_{{29}} \kappa + a_{{14}} \left( {1 + \kappa ^{2} } \right)\phi _{1} } \right)\cosh \left[ {h\kappa } \right]}}{{\kappa ^{2} }} \\ & + \frac{{h^{3} \beta \left( {5 - \left( { - 5 + h^{2} } \right)\phi _{1} } \right)\sin \left[ \alpha \right]}}{{15F\left( {1 + \beta } \right)}} \\ & + \frac{{\left( {a_{{26}} \left( {2 + h^{2} \kappa ^{2} } \right) - \kappa \left( {a_{{29}} + \kappa \left( {a_{{14}} - a_{{28}} + a_{{14}} h^{2} \phi _{1} } \right)} \right)} \right)\sinh \left[ {h\kappa } \right]}}{{\kappa ^{3} }} \\ \end{aligned}$$

Rearranging the terms of Eq. (57), the axial pressure gradient is obtained as

58 $$\begin{aligned} \frac{\partial\:p}{\partial\:x}& =\frac{15(1+\beta)}{{h}^{3}\beta\:\left(-5+\left(-5+{h}^{2}\right){\varphi\:}{1}\right)}\left(Q+\frac{h} {2520}\left(-2520\left({a}{13}+{a}{2}+{a}{9}\right) \right.\right.\\ &\quad \left.\left. +420\left(3+{a}{5}-2{a}{19}{a}{5}-2\left({a}{21}+{a}{4}\right){a}{6}\right){h}^{2}-42\left(12{a}{10}+\left(5+4{a}{18}\right){a}{5}-4{a}{21}{a}{6}\right){h}^{4} \right. \right. \\ &\quad \left. \left. -12\left(30{a}{23}+7{a}{17}{a}{5}\right){h}^{6}-5\left(56{a}{22}+9{a}{16}{a}{5}\right){h}^{8}\right. \right. \\ &\quad \left. \left. -21{h}^{2}\left(40\left({a}{19}+{a}{5}+{a}{4}{a}{6}\right)+\left(3-96{a}{10}+24{a}{18}-28{a}{5}-8{a}{4}{a}{6}\right){h}^{2} \right.\right. \right. \\ &\quad \left.\left.\left. +4\left(2{a}{10}+5{a}{17}+{a}{5}\right){h}^{4}+15{a}{16}{h}^{6}\right){\varphi}{1} \right.\right. \\ &\quad \left. \left. -84{h}^{2}\left(10-7{h}^{2}+{h}^{4}\right){{\varphi\:}{1}}^{2}\right)\right. \\ &\quad\left. +\frac{{h}^{3}\beta\:\left(-5+\left(-5+{h}^{2}\right){\varphi\:}{1}\right)\text{sin}\left[\alpha\:\right]}{15F\left(1+\beta\:\right)} \right. \\ &\quad\left. +\frac{-h\kappa\:\left(-2{a}{26}+{a}{29}\kappa\:+{a}{14}\left(1+{\kappa\:}^{2}\right){\varphi\:}{1}\right)\text{cosh}\left[h\kappa\:\right]+\left(-{a}{26}\left(2+{h}^{2}{\kappa\:}^{2}\right)+\kappa\:\left({a}{29}+\kappa\:\left({a}{14}-{a}{28}+{a}{14}{h}^{2}{\varphi\:}_{1}\right)\right)\right)\text{sinh}\left[h\kappa\:\right]}{{\kappa\:}^{3}}\right)\end{aligned}$$

The pressure difference across one wavelength is defined as

59 $$\:\varDelta\:P=\underset{0}{\overset{1}{\int\:}}\frac{\partial\:p}{\partial\:x}dx$$

Using the D’Alembert mass conservation Eq. (31), the stream function (obeying the Cauchy-Riemann equations, $\:\left(w=\frac{\partial\:\psi\:}{\partial\:y}\:and\:v=-\frac{\partial\:\psi\:}{\partial\:x}\right)$ is also readily derived as

60 $$\begin{aligned} \psi & = a_{{11}} y + a_{{13}} y + a_{2} y + a_{{27}} y + a_{9} y - \frac{{y^{3} }}{6} + \frac{{a_{{12}} y^{3} }}{3} \\ & + \frac{{a_{{25}} y^{3} }}{3} + \frac{{a_{{10}} y^{5} }}{5} + \frac{{a_{{24}} y^{5} }}{5} + \frac{{a_{{23}} y^{7} }}{7} + \frac{{a_{{22}} y^{9} }}{9} \\ & - \frac{{2a_{{26}} ycosh\left[ {y\kappa } \right]}}{{\kappa ^{2} }} + \frac{{a_{{29}} ycosh\left[ {y\kappa } \right]}}{\kappa } - \frac{{a_{{29}} sinh\left[ {y\kappa } \right]}}{{\kappa ^{2} }} \\ & - \frac{{a_{{14}} sinh\left[ {y\kappa } \right]}}{\kappa } + \frac{{a_{{28}} sinh\left[ {y\kappa } \right]}}{\kappa } + \frac{{a_{{26}} \left( {2 + y^{2} \kappa ^{2} } \right)sinh\left[ {y\kappa } \right]}}{{\kappa ^{3} }} \\ \end{aligned}$$

The wall shear stress (skin-friction coefficient), heat transfer coefficient (Nusselt number), and mass transfer coefficient (Sherwood number) in non-dimensional form are given by

61 $$\:{C}_{f}={h}^{{\prime\:}}{\left.\frac{\partial\:w}{\partial\:y}\right|}_{y\to\:h}$$

62 $$\:Nu={h}^{{\prime\:}}{\left.\frac{\partial\:\theta\:}{\partial\:y}\right|}_{y\to\:h}$$

63 $$\:Sh={h}^{{\prime\:}}{\left.\frac{\partial\:\xi\:}{\partial\:y}\right|}_{y\to\:h}$$

## Validation of results

**Fig. 3:**
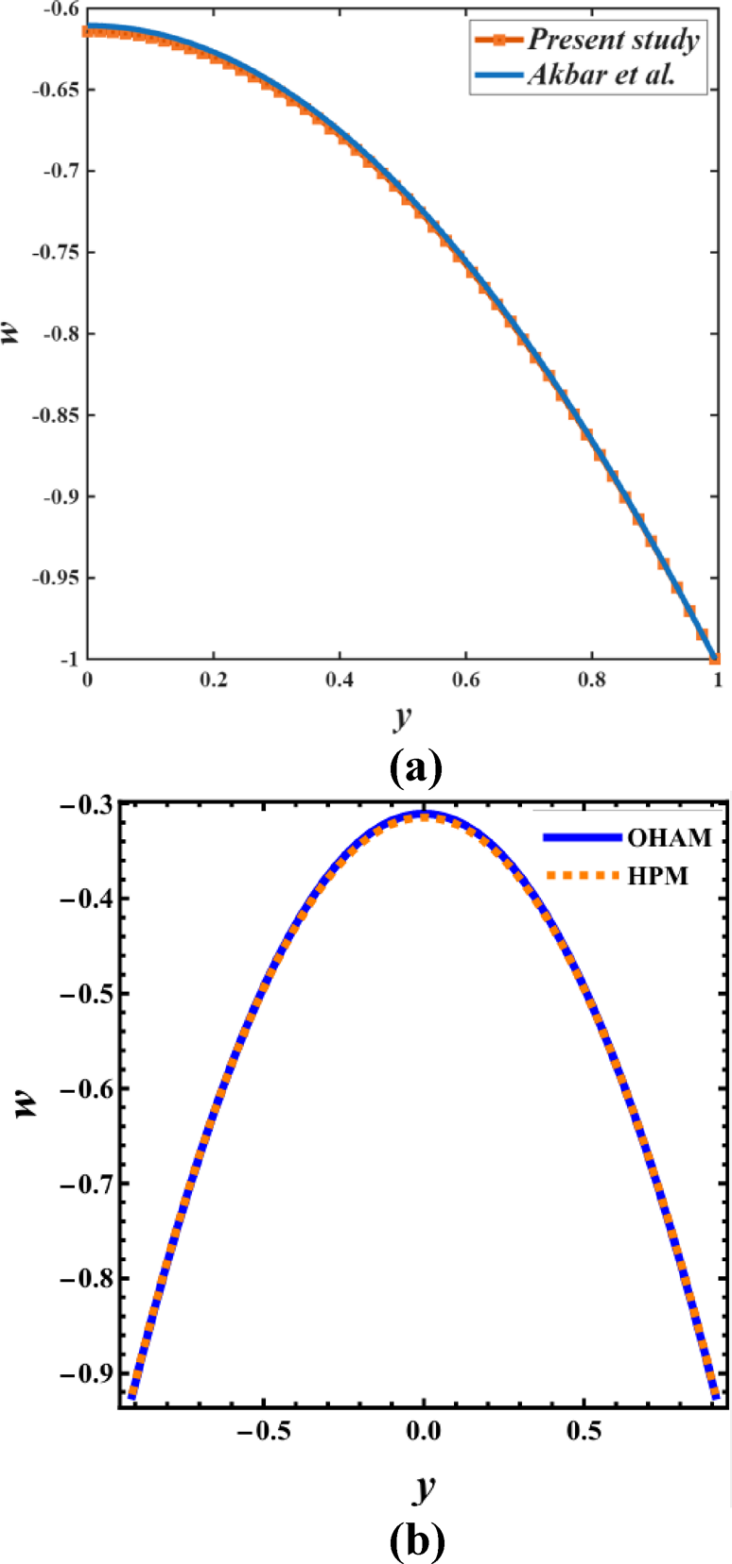
(**a**) Comparison of the velocity profile from the present study with Akbar et al.^43^. (**b**) Comparison of the present HPM results with the OHAM solution.

For validation, the current results are compared with both the analytical findings presented by Akbar et al.^43^ and the outcomes derived from other semi-analytical methods. To maintain a consistent comparison, all additional effects included in the current model, specifically the non-uniformity parameter, variable viscosity and thermal conductivity, buoyancy forces, and electric field, are set to zero. Additionally, the magnetic parameter is also considered to be zero in this comparison. Under these limiting conditions, the velocity profiles illustrated in Fig. 3a exhibit excellent agreement with the analytical results of Akbar et al.^43^, thereby confirming the accuracy and reliability of the current formulation. Similarly, Fig. 3b showcases the validation of the current HPM (Homotopy Perturbation Method) results against the OHAM (Optimal Homotopy Analysis Method). The close overlap between the two profiles indicates remarkable consistency throughout the domain, further validating the accuracy and robustness of the current analytical approach.

## Analysis of results

In this section, we present graphical results accompanied by physical explanations to illustrate how various flow parameters influence the velocity profile, temperature distribution, concentration profile, pressure gradient, and pressure rise. Streamlines are also included to clearly visualize the flow patterns and better understand the fluid motion under changing conditions. To analyze these graphical results, the numerical values of the relevant emerging parameters are required. The possible range of numerical values for the physically reliable parameters involved in the present work is taken from published resources and provided in Table 2 as follows.

**Table 2:** Numerical values of physical parameters.

Physical parameters	Range of the parameters^32,41,44^	Significance
$\:\kappa\:\:$	$\:1\:\text{t}\text{o}\:3$	Ensures moderate electroosmotic effects typically observed in microchannels.
$\:{U}_{hs}$	$\:-3\:\text{t}\text{o}\:3$	Captures both forward and reverse electroosmotic slip, maintaining realistic potential gradients across the EDL.
$\:{\varphi\:}_{1},\:{\varphi\:}_{2}$	$\:0.01\:\text{t}\text{o}\:1$	Stronger temperature dependence, enhancing flow and heat transfer.
$\:\alpha\:$	$\:0\:\text{t}\text{o}\:\frac{\pi\:}{2}$	Extent of gravity along the channel.
$\:Br$	$\:0.01\:\text{t}\text{o}\:0.6$	Measures heat generation due to viscous dissipation.
$\:\gamma\:$	$\:0.1\:\text{t}\text{o}\:0.7$	Stronger cilia action results in greater resistance to flow.
$\:Gr,\:Gm$	$\:0.1\:\text{t}\text{o}\:2$	Moderate buoyancy-driven convection.
$\:Nt$, $\:Nb$	$\:0.05\:\text{t}\text{o}\:1.5$	Particle migration due to temperature gradient, Nanoparticle diffusion effect.
$\:{K}_{nu}$	$\:<$0	Convergent shape of the channel.

### Velocity profile

The axial velocity $\:w$ within the inclined microchannel, typically displays a symmetric distribution around the centerline of the channel, peaking in the core area and gradually decreasing towards the walls due to viscous resistance. Variations in governing parameters, such as inclination angle, Casson parameter, mass Grashof number, thermal Grashof number, variable liquid properties, and electroosmotic effect, modify both the peak values and the near-wall gradients. To assess the impact of wall-anchored cilia, three specific scenarios are examined: $\:\gamma\:=0$ (without cilia), $\:\gamma\:=0.1$(short cilia), and $\:\gamma\:=0.5$ (long cilia).

In the case $\:\gamma\:=0$, the channel is operated in the absence of ciliary propulsion, and the imposed electroosmotic and heat-induced forces sustain the resultant fluid motion. At $\:\gamma\:>0$, the coordinated beating of the cilia causes a metachronal wave that drives the fluid. The motion of the ciliary tips drags the fluid, and this reduces the maximum velocity at the centerline of the channel. As the length of the cilia increases, the effect of drag increases, and the velocity distribution across the channel changes more significantly. This ordered scheme -between smooth walls ($\:\gamma\:=0$) to somewhat obstructed flow ($\:\gamma\:=0.1$) and highly obstructed flow ($\:\gamma\:=0.5$) offers an initial framework within which the influence of several governing parameters on the axial velocity profiles can be observed when subjected to varying levels of mechanical resistance by the walls.

**Fig. 4:**
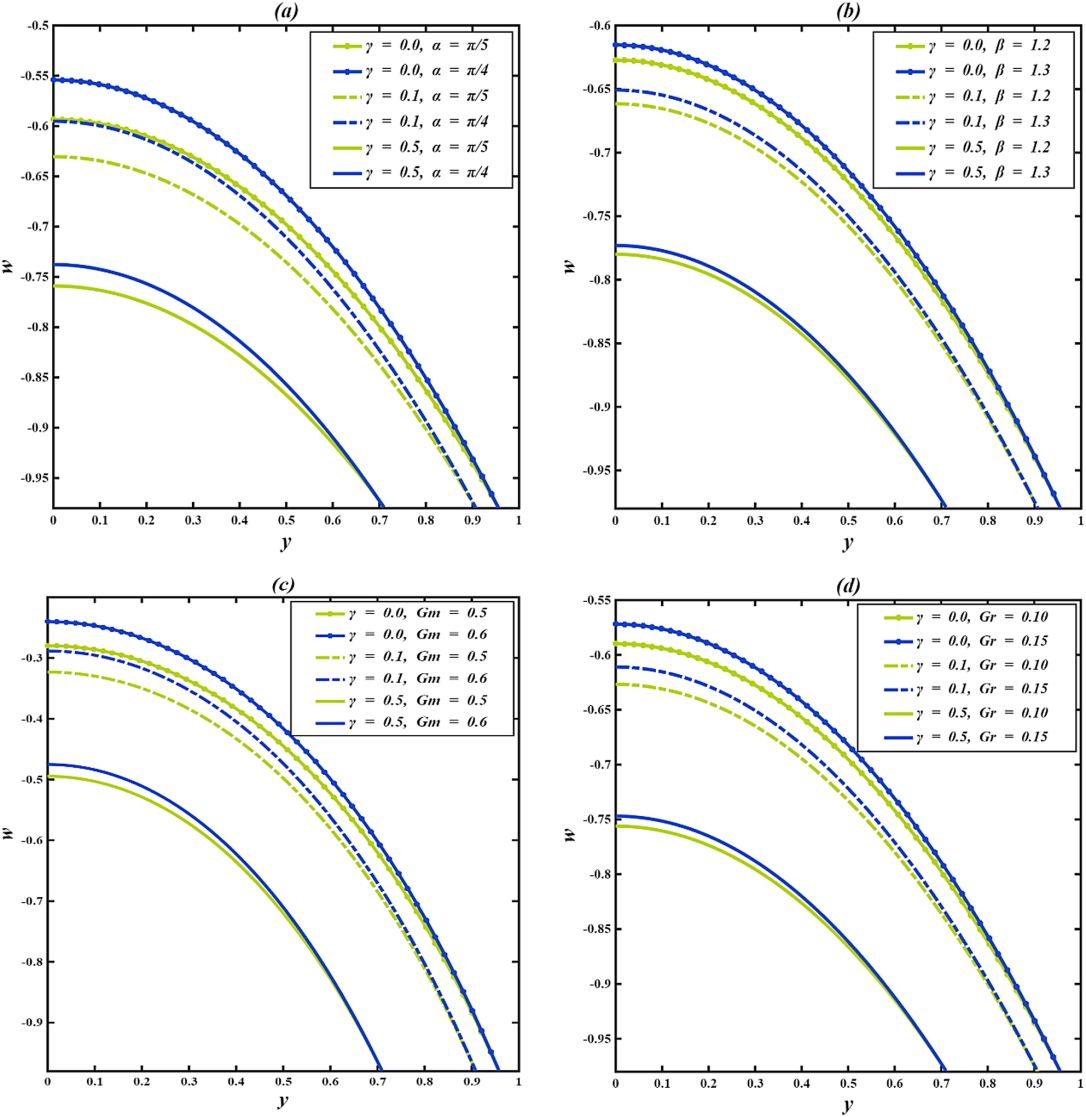

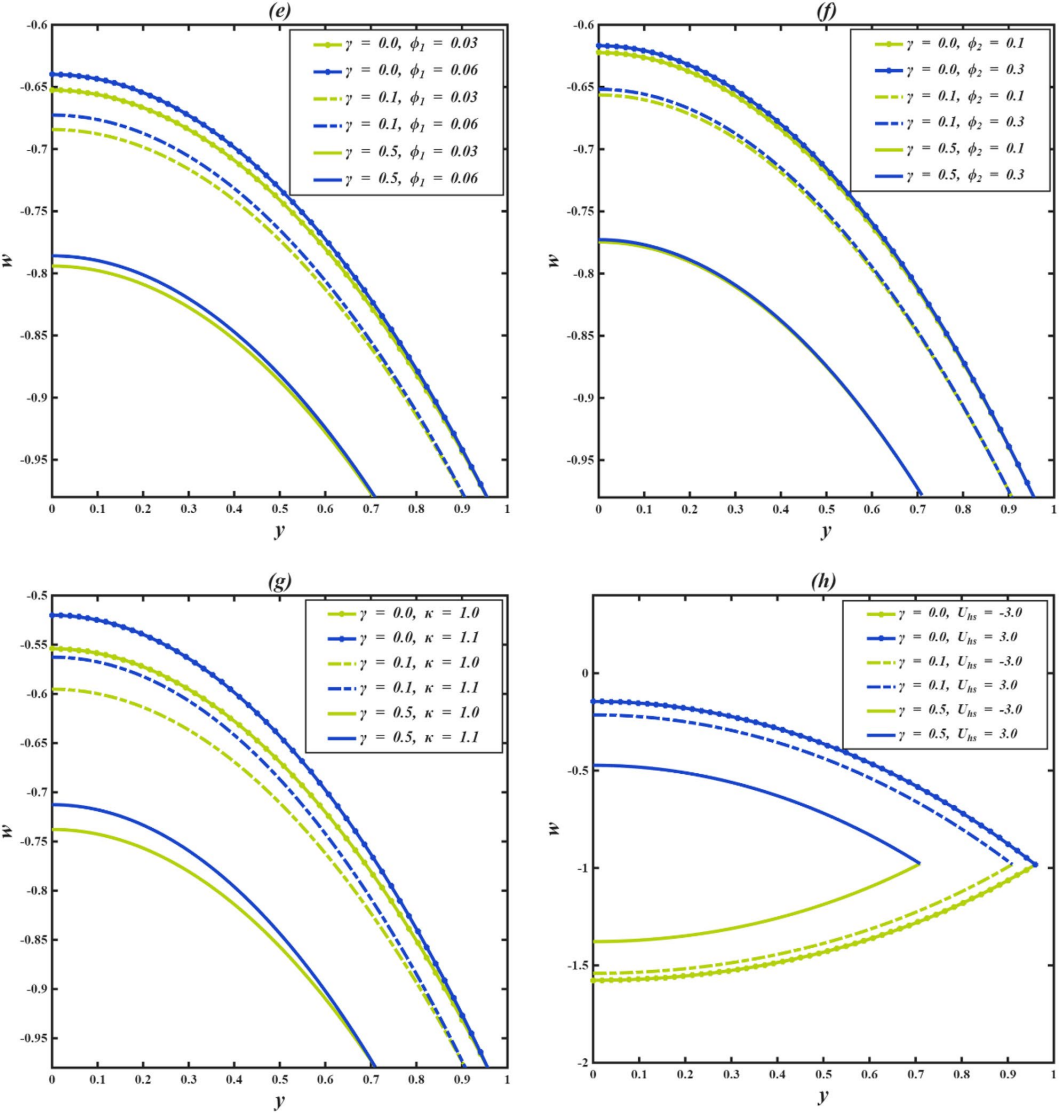
Effect of cilia lengths ($\:\gamma\:$) on axial velocity profiles $\:w\left(y\right)$ under varying physical parameters: (**a**) inclination angle ($\:\alpha\:$), (**b**) Casson fluid parameter ($\:\beta\:$), (**c**) mass Grashof number ($\:Gm$), (**d**) thermal Grashof number ($\:Gr$), (**e**) viscosity variation parameter ($\:{\phi\:}_{1}$), (**f**) thermal conductivity variation parameter ($\:{\phi\:}_{2}$), (**g**) electroosmotic parameter ($\:\kappa\:$), and (**h**) Helmholtz-Smoluchowski velocity ($\:{U}_{hs}$).

Figure 4a illustrates the axial velocity profiles of $\:w$ for different cilia lengths and inclination angles $\:\alpha\:$. With an increase in inclination angle, the part of the gravitational acceleration in the flow direction increases with each cilia length. The axial component of gravity assists fluid motion by acting in the same direction as the cilia-induced forces, thereby reducing flow resistance. However, as $\:\gamma\:$ increases, the dominance of viscous and form drag from cilia overcomes this body force, leading to a reduced acceleration effect. Figure 4b illustrates how the axial velocity $\:w$ varies with changes in the Casson fluid parameter $\:\beta\:$ for different cilia lengths $\:\gamma\:$. For each fixed value of $\:\gamma\:$, increasing $\:\beta\:$ leads to a slight increase in the velocity profile. This increase can be explained physically by the fact that a higher Casson fluid parameter decreases the yield stress and effective viscosity of the fluid, which results in a reduction in resistance to deformation. Consequently, this promotes greater fluid mobility and higher axial velocity. Even though increasing $\:\beta\:$ encourages velocity, the influence of longer cilia reduces this advantage due to increased interaction with the walls and drag. Figure 4c illustrates how axial velocity $\:w\:$changes for different mass Grashof number $\:Gm\:$across various cilia lengths $\:\gamma\:$. For each length of cilia, higher $\:Gm$ amplifies the solutal density difference, producing a stronger buoyant force that supplements the axial pressure gradient. This additional driving force enhances fluid acceleration and elevates the centerline velocity, particularly in regions with minimal ciliary resistance. This enhancement gradually weakens as cilia-induced drag increases, though the positive trend with $\:Gm$ persists in all cases. Figure 4d illustrates the axial velocity profiles $\:w\:$corresponding to various thermal Grashof numbers $\:Gr$ and cilia lengths $\:\gamma\:$. For all values of $\:\gamma\:$, an increase in $\:Gr$ results in enhanced fluid velocity, attributed to the augmented thermal buoyancy force that propels upward flow within the channel. Because when the temperature near the wall increases, density decreases, and gravity acting on this density difference produces an upward buoyancy force. This thermal buoyancy propels the fluid upward, and as $\:Gr$ increases, this upward driving force strengthens, hence the observed enhancement in fluid velocity. When mechanical resistance is low, the impact is still notable; however, as γ rises, the acceleration driven by buoyancy is increasingly opposed by vortex formation around the cilia. Figure 4e demonstrates how the temperature dependence of the viscosity parameter affects the axial velocity $\:w\:$for different cilia lengths $\:\gamma\:$. An increase in $\:{\phi\:}_{1}$ results in elevated velocities throughout the channel for all values of $\:\gamma\:$. This phenomenon occurs because a higher $\:{\phi\:}_{1}$ indicates a quicker decrease in fluid viscosity with temperature, thus reducing resistance to shear and improving flow capability. The reduction occurs because increasing thermal energy weakens the cohesive intermolecular forces within the fluid, allowing layers to slide past each other more easily. This enhancement in flow is progressively reduced with greater cilia lengths due to increased wall interactions and momentum loss. Figure 4f depicts the variation in axial velocity $\:w$ in relation to thermal conductivity $\:{\phi\:}_{2}$ parameter at various cilia lengths $\:\gamma\:.$ As $\:{\phi\:}_{2}$ increases, the axial velocity rises for all $\:\gamma\:$ values. The rise in thermal conductivity with temperature occurs because the increased molecular agitation at higher temperatures enhances energy transport between particles through more frequent collisions. This microscopic increase in energy exchange efficiency enables heat to diffuse more rapidly across the fluid, thereby strengthening the temperature-driven flow and electroosmotic coupling. With longer cilia, wall drag again limits this gain, and at $\:\gamma\:=0.5$, the velocity curves for different $\:{\phi\:}_{2}$ nearly converge, indicating that mechanical resistance dominates over the parameter’s driving effect. Figure 4g illustrates that the fluid velocity $\:w\:$inside the channel is affected by both the electroosmotic parameter κ and the length of the cilia $\:\gamma\:$. Since κ is the inverse of the Debye length, increasing corresponds to a higher ionic concentration in the electrolyte, which compresses the electric double layer and intensifies the local electric field near the charged surface. The greater field strength enhances ion-fluid momentum transfer through electrostatic attraction between the mobile counter-ions and the wall potential. This microscopic increase in electrostatic interaction results in stronger electroosmotic slip and, consequently, a higher axial velocity in the channel. The increase is most effective when cilia are absent, diminished when cilia are moderate, and suppressed when cilia are long. Figure 4h demonstrates how the Helmholtz-Smoluchowski velocity parameter $\:{U}_{hs}$ influences $\:w\:$for various$\:\:\gamma\:$. A positive value of $\:{U}_{hs}$ indicates that the electric field drives mobile counter-ions along the flow direction, transferring momentum to the adjacent fluid layers and accelerating forward motion. Conversely, a negative $\:{U}_{hs}$ reverses the direction of this electrostatic force, pulling the near-wall fluid backward. For $\:\gamma\:=0$, the influence of $\:{U}_{hs}$ is at its peak, with $\:{U}_{hs}=+3$ leading to the highest forward velocity and $\:{U}_{hs}=-3$ yielding the minimal reverse velocity. As the introduction of longer cilia creates increased hydrodynamic drag, which reduces the forward velocity when $\:{U}_{hs}>0$. On the other hand, when $\:{U}_{hs}<0$, longer cilia amplify the strength of reverse flow, as the combined effects of electroosmotic resistance and ciliary drag accelerate the fluid in the opposite direction.

The resistance created by cilia affects the axial velocity distributions in the inclined microchannel, as fluid properties determine the effect of thermal and electrokinetic forces within the channel. There are also critical roles about the length of the cilia: short cilia or cilia which are nonexistent assist in the improvement of the flow but long cilia make the flow resistant and weaker. The findings underscore the need to consider the selection of microchannel parameters and cilia in order to achieve a high level of efficiency in biomedical and microfluidics.

### Temperature profile

The temperature field $\:\theta\:$, in the inclined microchannel tends to increase towards the centerline and decrease towards the walls, with heat conductivity being taken out towards the channel surfaces, which are cooled. A longer cilia $\:\gamma\:$ length is more likely to decrease the fluid temperature as longer cilia limit fluid movement, decrease convective heat transfer, and enhance heat transfer beside the walls. Conversely, in the absence of cilia ($\:\gamma\:=0$), there is low resistance to the flow in the channel, which means that more heat is concentrated around the central point in the channel. As $\:\gamma\:$ grows to moderate or long lengths, mechanical obstruction improves wall interaction and reduces bulk heating, resulting in a lower temperature profile.

**Fig. 5:**
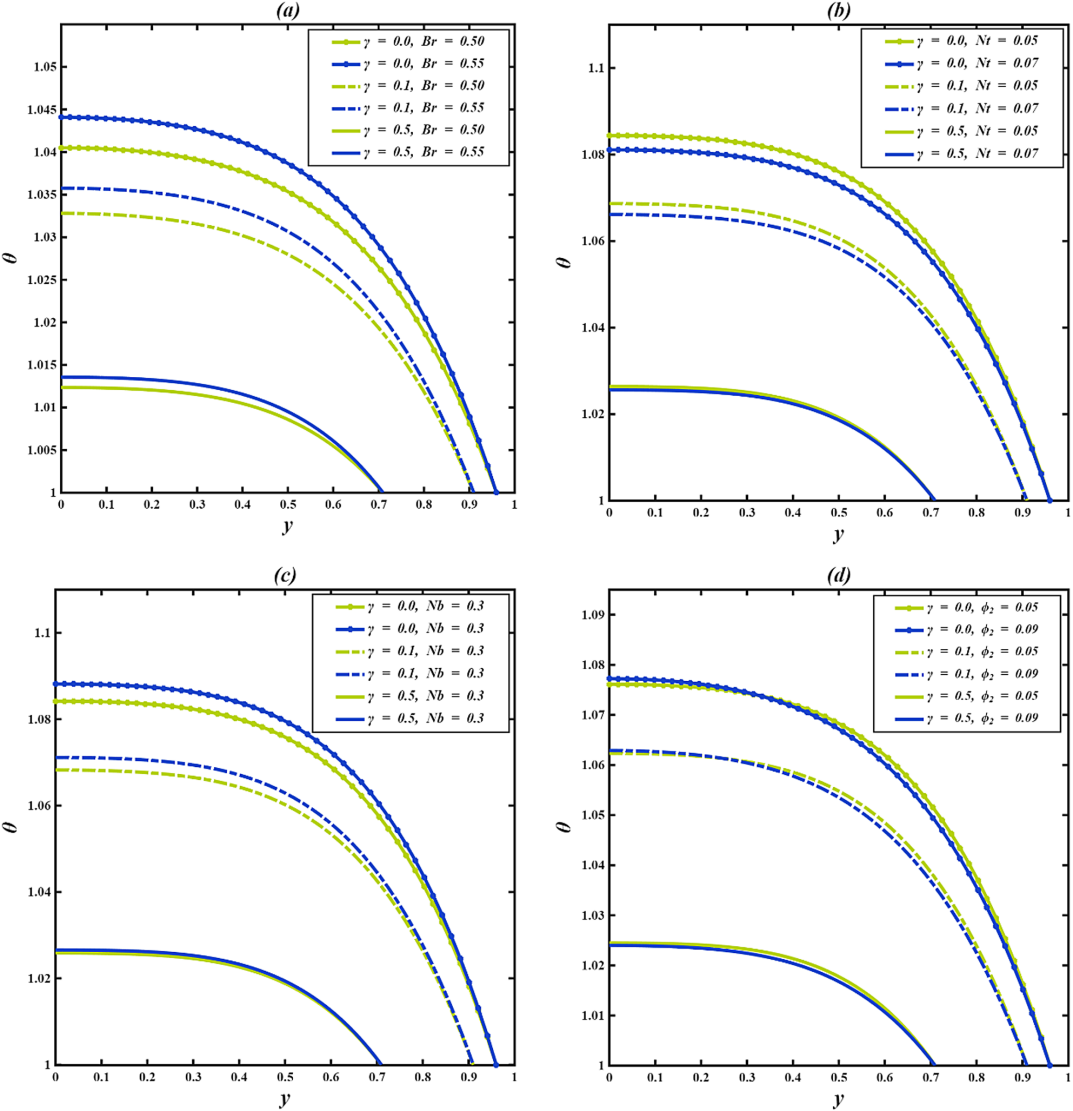

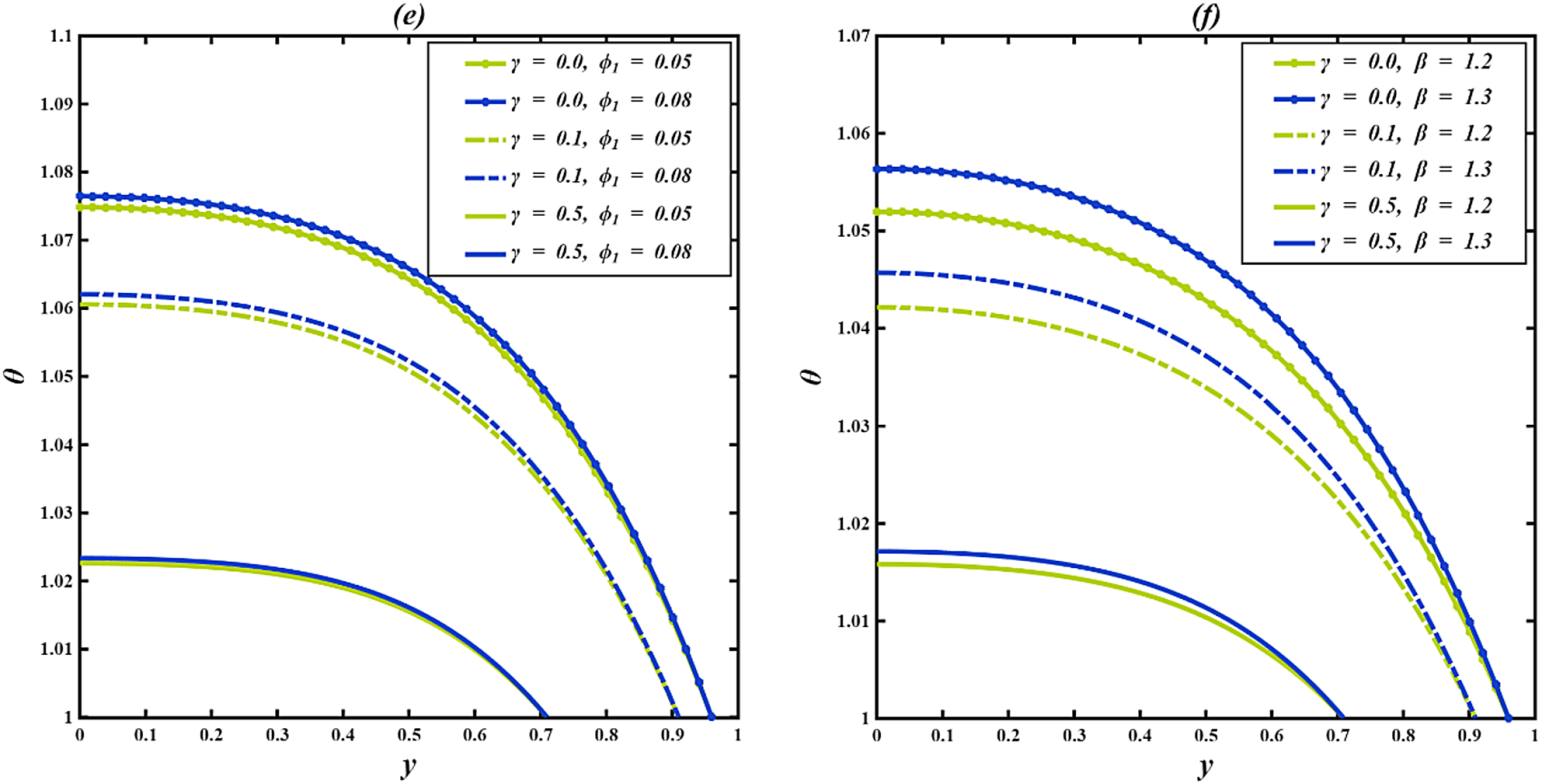
Effect of cilia lengths ($\:\gamma\:$) on temperature profiles $\:\theta\:\left(y\right)$ under varying physical parameters: (**a**) Brinkman number ($\:Br$), (**b**) thermophoresis parameter ($\:Nt$), (**c**) Brownian motion parameter ($\:Nb$), (**d**) thermal conductivity variation parameter ($\:{\phi\:}_{2}$), (**e**) viscosity variation parameter ($\:{\phi\:}_{1}$), and (**f**) Casson fluid parameter ($\:\beta\:$).

Figure 5a illustrates that the temperature profile $\:\theta\:$ increases with a higher Brinkman number $\:Br$, while it decreases as the cilia length $\:\gamma\:$ increases. In physical terms, a greater $\:Br$ indicates that a larger portion of the mechanical work done by viscous forces is converted into internal energy through frictional heating. As fluid layers slide past each other, increased shear at elevated $\:Br$ leads to stronger molecular agitation and energy loss as heat, thereby elevating the temperature. The increase is most significant when $\:\gamma\:=0$, as there is minimal resistance, whereas at $\:\gamma\:=0.1$ and 0.5, the heating continues but is gradually mitigated by ciliary drag. Figure 5b illustrates the effect of the thermophoresis parameter $\:Nt$ on $\:\theta\:$ for different cilia lengths. It is noted that as $\:Nt$ rises, the temperature within the fluid decreases across all values of $\:\gamma\:$. This is a result of thermophoresis, or the Soret effect, where particles move from hotter regions to cooler ones in response to temperature gradients. Particles in a hotter region have more kinetic energy, and consequently, they move towards cooler regions, redistributing the thermal energy out of that higher-temperature area. This thermally induced particle migration is, however, additionally enhanced by an increase in $\:Nt$, leading to a more efficient cooling and, subsequently, to a lower overall temperature. Temperature profiles are lower for $\:\gamma\:=0.5$, indicating that long cilia induce more cooling, which increases the hydrodynamic drag. Hence, the convective heat transfer diminishes, enabling thermophoresis to have a more significant impact. Conversely, when $\:\gamma\:=0$, particle movement is greater, but minimal flow obstruction results in increased convective mixing, causing slightly elevated overall temperatures even with thermophoresis at play. Figure 5c demonstrates the influence of the Brownian motion parameter $\:Nb$ on the temperature distribution $\:\theta\:\:$across different cilia lengths $\:\gamma\:$. An increase in $\:Nb$ results in a rise in fluid temperature for all $\:\gamma\:$ values. This phenomenon occurs because Brownian motion signifies the random movement of nanoparticles, which enhances energy transfer between particles and the base fluid. As $\:Nb$ escalates, a greater amount of thermal energy is retained in the fluid due to intensified microscopic agitation, consequently elevating the macroscopic temperature. The heating effect is most pronounced when $\:\gamma\:=0$ and decreases with longer cilia because of limited particle motion and greater heat loss to the walls. Figure 5d displays the temperature profile $\:\theta\:\:$for different values of the thermal conductivity variation parameter $\:{\phi\:}_{2}$ and cilia lengths $\:\gamma\:$. For $\:\gamma\:=0\:and\:0.1,$ a mixed pattern is noted: the temperature experiences a slight increase near the center, with the profiles converging mid-channel before decreasing near the wall as $\:{\phi\:}_{2}$ increases. A higher $\:{\phi\:}_{2}$ increases the fluid’s temperature-dependent thermal conductivity, allowing heat to be transferred more efficiently from the hotter core toward the cooler wall. This enhanced conduction strengthens the temperature gradient and promotes faster heat loss, resulting in a lower fluid temperature near the wall. At $\:\gamma\:=0.5$, the temperature profiles stay relatively close but exhibit a distinct drop with an increase in $\:{\phi\:}_{2}$, demonstrating that while the mechanical resistance from longer cilia restricts fluid motion, the improved heat conduction still induces a significant cooling effect. Figure 5e illustrates the temperature distribution $\:\theta\:\:$as a function of the viscosity variation parameter $\:{\phi\:}_{1}$ for varying cilia lengths $\:\gamma\:$. Across all values of $\:\gamma\:$, an increase in $\:{\phi\:}_{1}$ results in a noticeable increase in fluid temperature. This is due to the fact that a higher $\:{\phi\:}_{1}$ signifies a more rapid decrease in viscosity with temperature, consequently reducing internal friction and permitting the fluid to flow more easily. The subsequent rise in molecular mobility facilitates the retention of thermal energy, leading to a higher overall temperature. This pattern resembles behaviors observed in thermal processing scenarios, such as crude oil refinement, where elevated temperatures decrease viscosity, speed up molecular movement, and result in increased thermal activity. The impact is most pronounced at $\:\gamma\:=0$ and gradually less pronounced at $\:\gamma\:=0.1,\:and\:\gamma\:=0.5$. Figure 5f depicts how the Casson parameter $\:\beta\:$ affects the temperature distribution $\:\theta\:\:$for different cilia lengths $\:\gamma\:$. For every value of $\:\gamma\:$, an increase in $\:\beta\:$ leads to a rise in the fluid temperature because the higher deformation rate amplifies viscous heating and energy dissipation within the fluid. The effect is stronger when $\:\gamma\:=0$, where flow obstruction is minimal, and diminishes for larger $\:\gamma\:$ as cilia-induced resistance limits the conversion of mechanical energy into heat.

The temperature distribution in an inclined, ciliated results from a balance between energy enhancing factors (viscous dissipation, electro-osmotic, Brownian motion, and thermal buoyancy), and energy dissipating effects from ciliary resistance. Parameters including $\:Br$, $\:\beta\:$, $\:Nb$ typically increase fluid temperature by facilitating internal heat generation or transport. Conversely, factors like the $\:Nt$ and $\:{\phi\:}_{2}$ generally lead to a decrease in temperature by improving heat diffusion from hotter areas. Notably, $\:\gamma\:$ acts as an essential moderating variable. This interplay is key for controlling thermal behavior in microscale devices.

### Concentration profile

The concentration profile of nanoparticles, denoted as $\:\xi\:$, is minimal at the center of the channel (y = 0) and rises toward the walls (y = 1), illustrating that particles are inclined to move toward the edges. Cilia that are longer elevate the concentration profile, suggesting that there are greater particle densities along the channel. This increase occurs because longer cilia affect the flow structure near the walls, which reduces the escape of particles and promotes accumulation throughout the entire domain.

**Fig. 6:**
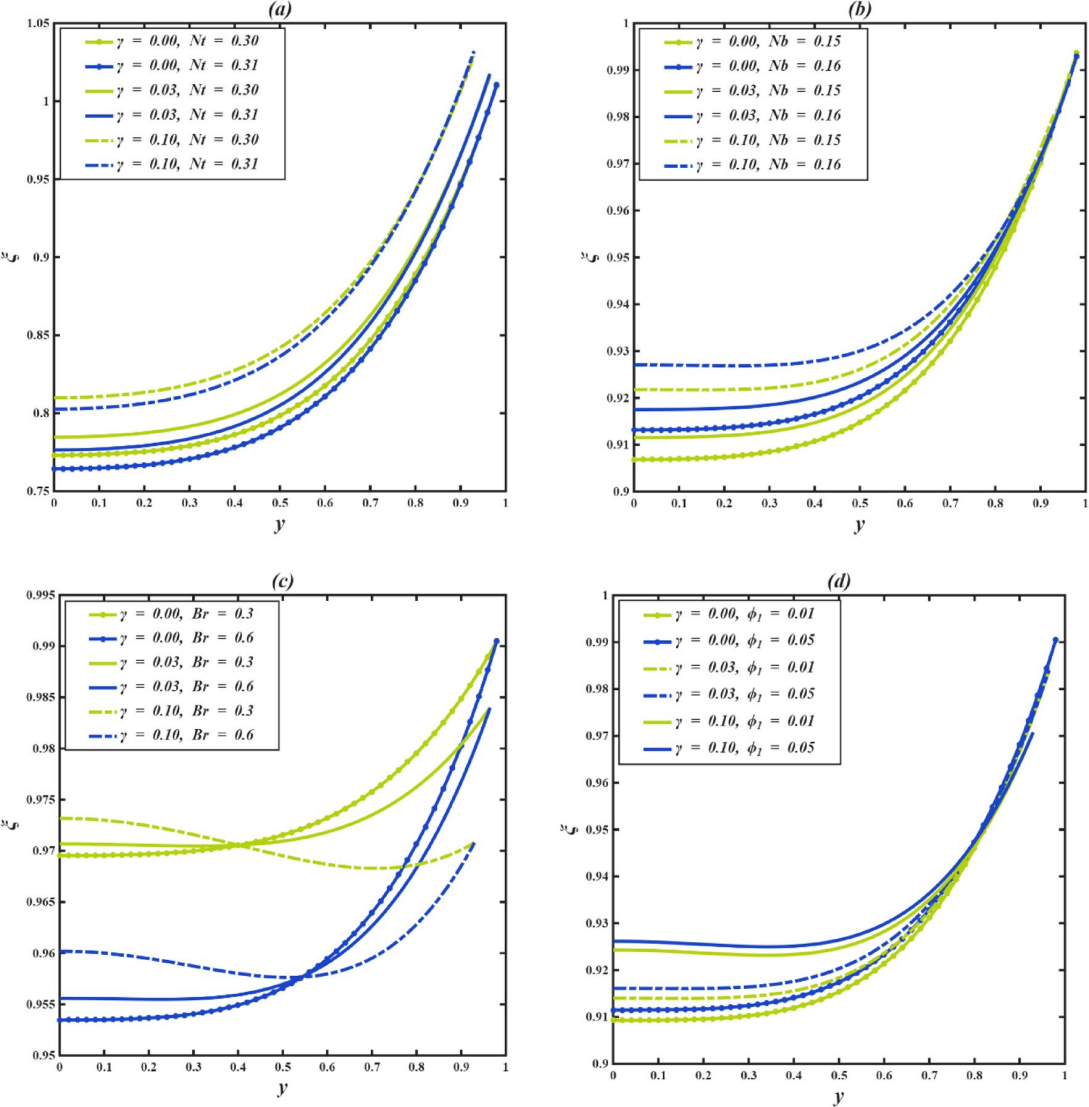
Effect of cilia lengths ($\:\gamma\:$) on temperature profiles $\:\theta\:\left(y\right)$ under varying physical parameters: (**a**) Brinkman number ($\:Br$), (**b**) thermophoresis parameter ($\:Nt$), (**c**) Brownian motion parameter ($\:Nb$), (**d**) thermal conductivity variation parameter ($\:{\phi\:}_{2}$), (**e**) viscosity variation parameter ($\:{\phi\:}_{1}$), and (**f**) Casson fluid parameter ($\:\beta\:$).

Figure 6a illustrates how the thermophoresis parameter $\:Nt$ affects the nanoparticle concentration profile $\:\xi\:\:$for various cilia lengths $\:\gamma\:$. It is noted that an increase in $\:Nt$ results in a decrease in concentration throughout the channel for all $\:\gamma\:$ values. This results from stronger thermophoretic forces push nanoparticles away from the heated central area towards the cooler walls, thereby diminishing the overall concentration. The reduction is particularly prominent near the middle of the channel. In the region close to the wall (y→1), the curves for various *Nt* values are almost indistinguishable, suggesting that thermophoretic effects are minimal in that area. Although this outward migration happens for all $\:\gamma\:$, the decrease is somewhat mitigated with longer cilia. Figure 6b demonstrates how the Brownian motion parameter $\:Nb$ influences the nanoparticle concentration profile $\:\xi\:\:$across different cilia lengths $\:\gamma\:$. It is evident that for every $\:\gamma\:$ value, an increase in $\:Nb$ results in a slight elevation in concentration, attributed to enhanced random movement and interactions between particles and fluid. As $\:\gamma\:$ increases, the distinction between the profiles for various $\:Nb$ values diminishes, signifying that the hydrodynamic resistance caused by cilia increasingly influences particle transport, thereby lowering the relative effect of Brownian motion. Figure 6c illustrates the effect of the Brinkman number $\:Br$ on the nanoparticle concentration profile $\:\xi\:\:$for various cilia lengths $\:\gamma\:$. Increasing $\:Br$ results in a steady decline in concentration, which can be explained by the heightened viscous dissipation that elevates fluid temperature, thereby enhancing nanoparticle diffusion via Brownian motion and thermophoresis. Increased ciliary length, on the other hand, elevates the overall concentration by hindering convective transport and restricting particle release. As $\:\gamma\:$ rises, the separation between profiles for varying $\:Br$ values diminishes, suggesting that the resistance from cilia increasingly counteracts the dispersive influence of viscous heating. Figure 6d shows that a higher $\:{\phi\:}_{1}$ would greatly increase the concentration of nanoparticles in the channel. The larger $\:{\phi\:}_{1}$ is, the faster the viscosity will reduce with temperature, resulting in greater fluid velocity and, thus, particles will be suspended more effectively as convective mixing increases. This leads to an even distribution, and a significant loss of particles is avoided. As the distance between the $\:{\phi\:}_{1}$ curves does not change much with $\:\gamma\:$, the increase in concentration is clear throughout all the $\:\gamma\:$ values. Even though the effect of longer cilia is supplementary and does not alter the influence of $\:{\phi\:}_{1}$, the longer cilia help to retain the flow, but at a moderate height.

Heat-induced movement, Brownian movement, viscous heating, and viscous variations all affect the concentration of nanoparticles, with longer cilia enhancing retention. $\:Nt$ reduces the central concentrations, $\:Nb$ causes a slight increment, and $\:Br$ focuses the particles onto the walls, and $\:{\phi\:}_{1}$ increases the concentrations equally. Long cilia minimize the loss of particles and also tune these effects. The application of this regulation is critical in targeted drug delivery and lab-on-chip technologies.

### Pressure gradient profiles

**Fig. 7:**
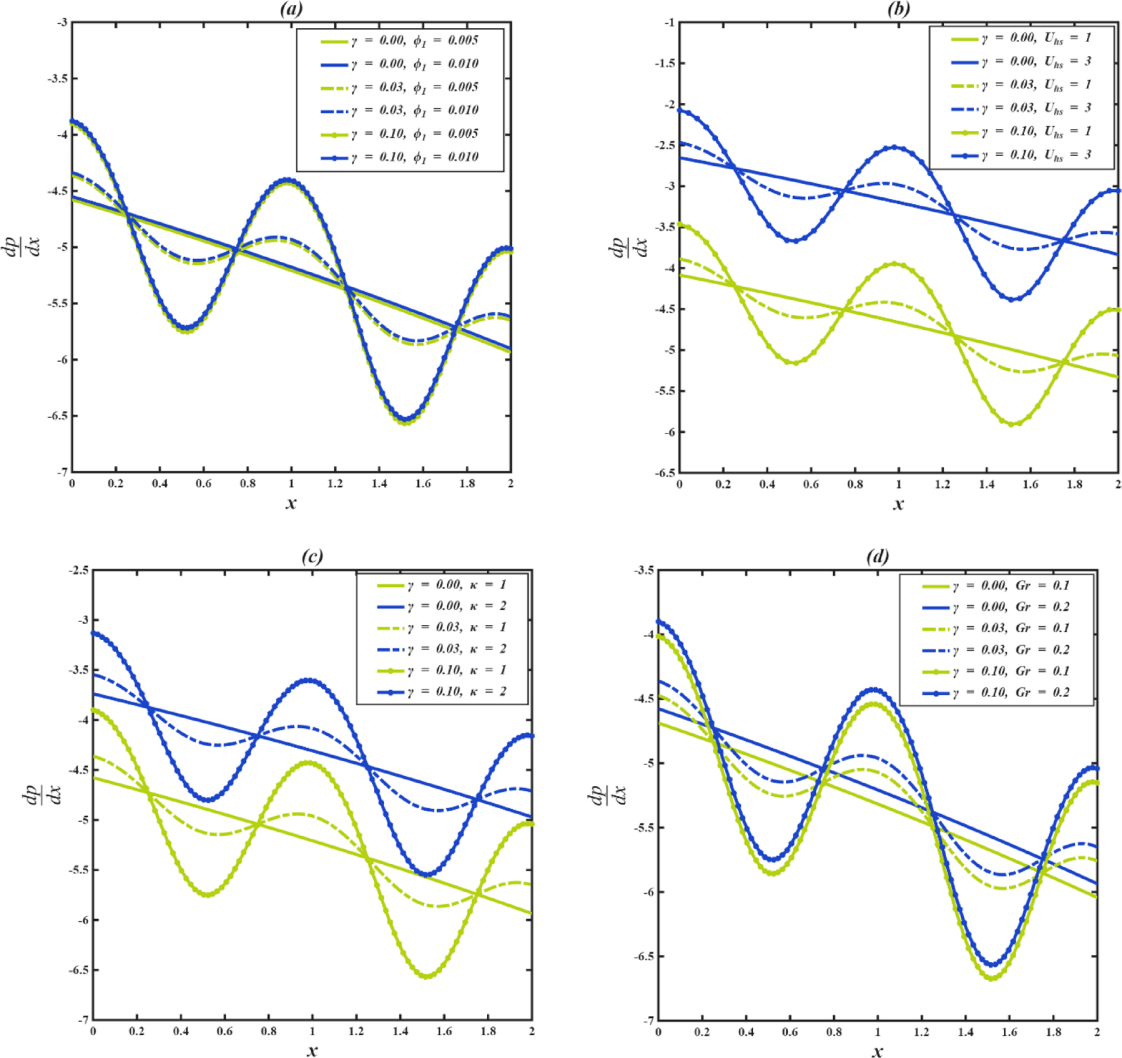
Effect of cilia lengths ($\:\gamma\:$) on axial pressure gradient $\:\frac{dp}{dx}$ under varying physical parameters: (**a**) viscosity variation parameter ($\:{\phi\:}_{1}$), (**b**) Helmholtz-Smoluchowski velocity ($\:{U}_{hs}$), (**c**) electroosmotic parameter ($\:\kappa\:$), and (**d**) thermal Grashof number ($\:Gr$).

Figure 7a–d represents how the level of dependence of the pressure gradient is on the appropriate parameters of flow. In this case, periodic constriction in the channel is created by the metachronal wave that is caused by the synchronized movement of cilia. When the wave crest passes onwards, it becomes narrower, making the passage in which the fluid flow occurs momentarily narrower and leading to a greater gradient of pressure needed to maintain the flow. The channel is wider on the troughs, which lie between two consecutive wave crests and present less opposition; therefore, less pressure is needed to cause the fluid to move. Although the physical channel structure remains constant, the differences in the internal flow space resulting from the action of cilia lead to a rhythmic variation in the pressure requirements along the channel’s length. This process of closure and dilation, propelled by the movement of the cilia, is vital in ensuring the effective flow of fluid through pressure flow and cilia action. In the case of $\:\gamma\:$ = 0, the cilia do not exist, and thus the movement of the fluid is purely electroosmotic. In such a case, the pressure gradient is linear and regular, with values greater at the center of the channel and lower at the walls, as no waves are formed to disrupt the flow. When cilia are introduced ($\:\gamma\:$ = 0.03) metachronal waves are produced, which cause periodic constrictions and dilation of the channel, as well as give rise to an oscillatory profile of pressure gradient. These waves are stronger when the cilia length is longer (e.g., $\:\gamma\:$ = 0.10) and the resultant curves have higher peaks and deeper troughs in the pressure gradient. This has a higher amplitude since the cilia are longer, resulting in increased resistance to flow and, consequently, reduced axial velocity. A stiffer pressure gradient is needed to maintain the flow rate constant, which causes the profile to be shifted upwards.

In every case ($\:{\phi\:}_{1}$, $\:{U}_{hs}$, $\:\kappa\:$, $\:Gr$), the greater one of the parameters is, the stronger the driving force in the channel. For instance, by altering viscosity ($\:{\phi\:}_{1}$), boosting the Helmholtz-Smoluchowski electroosmotic effect ($\:{U}_{hs}$), strengthening the electric field ($\:\kappa\:$), or enhancing buoyancy ($\:Gr$). These changes not only speed up the fluid but also enhance internal shear, viscous dissipation, and ciliary wave and geometrical restriction resistance. As a result, the higher flow requires a higher pressure gradient in order to sustain it. This effect is amplified by longer cilia because they further obstruct the axial flow and reduce the velocity and the system has to generate still more pressure to overcome the joint viscous and geometric resistance. With the increasing intensity of the driving parameters, the relevant growth of the internal resistance lowers the magnitude of velocity, and the pressure gradient must also increase correspondingly.

### Pressure difference analysis

**Fig. 8:**
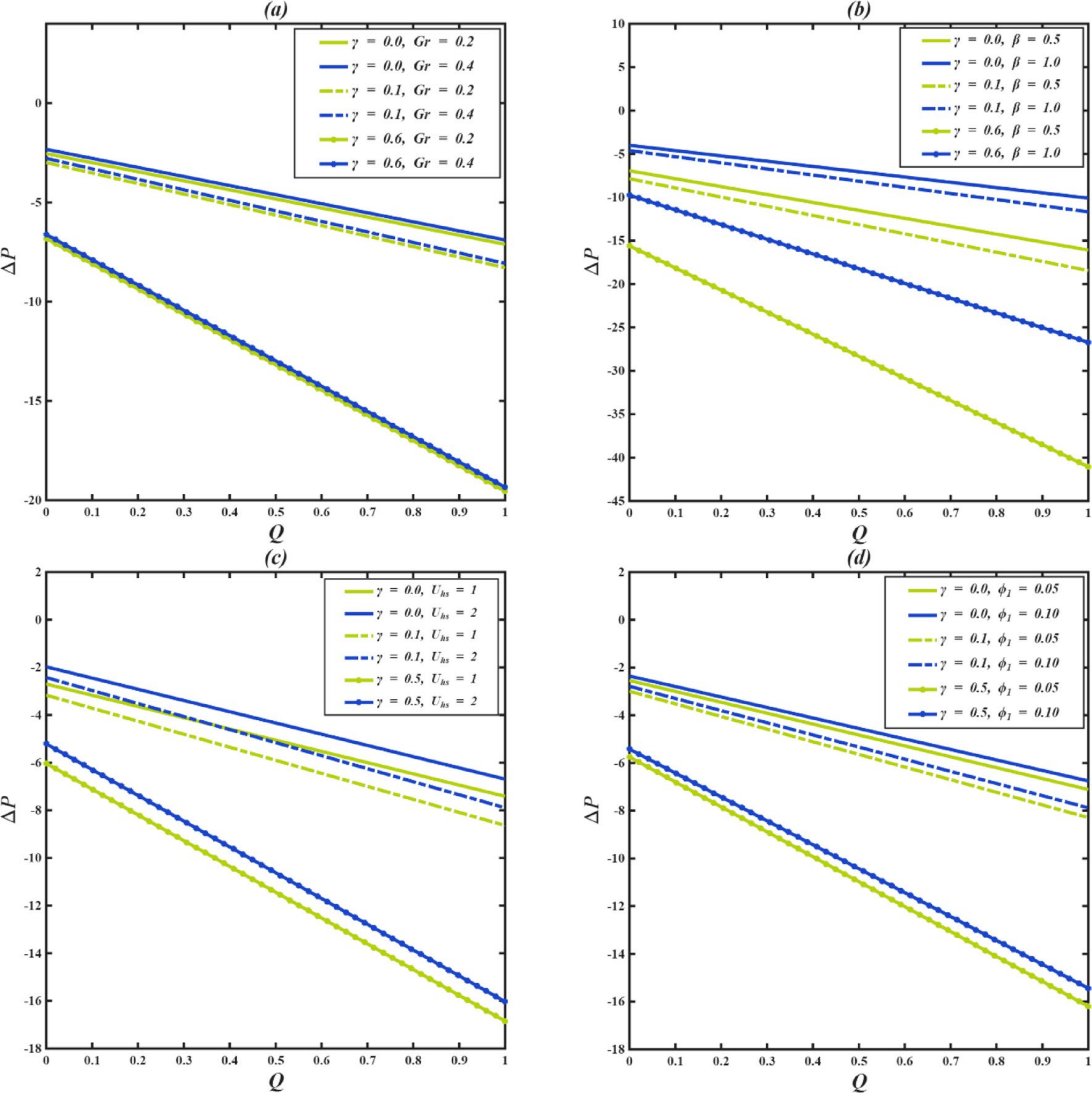
Effect of cilia lengths ($\:\gamma\:$) on the pressure difference $\:\varDelta\:P$ under varying physical parameters: (**a**) thermal Grashof number ($\:Gr$), (**b**) Casson fluid parameter ($\:\beta\:$), (**c**) Helmholtz-Smoluchowski velocity ($\:{U}_{hs}$), and (**d**) viscosity variation parameter ($\:{\phi\:}_{1}$).

Figure 8a–d demonstrates how the pressure difference ($\:\varDelta\:P$) correlates with the volumetric flow rate ($\:Q$) for different values of the cilia length parameter ($\:\gamma\:$), along with other key parameters. $\:\varDelta\:P$ represents the total pressure necessary to maintain a specified flow rate, factoring in both external forces and cilia-facilitated transport. In all scenarios, a linear inverse correlation is noted between $\:\varDelta\:P$ and $\:Q$, which aligns with the behavior expected from pressure-driven flow in microchannels. The relationship between pressure and flow can be divided into three separate regions based on the value of $\:\varDelta\:P$. When the difference in pressure is positive, the difference in pressure acts against the movement that the cilia are stimulating, meaning that the external pressure counteracts the movement caused by the cilia. Depending on the strength of the opposing pressure, this situation can result in the decrease or even inversion of the fluid movement. In the case of $\:\varDelta\:P=0$, the only contributions to the flow have to be made by cilia, which implies that when there is no external pressure, unhindered cilia-driven pumping can be achieved. Finally, at $\:\varDelta\:P$ that is lower than zero, the external pressure assists the ciliary movement, and the flow increases; the two forces derive from each other to enhance fluid pumping.

Figure 8a,c demonstrates that as $\:Gr\:and\:{U}_{hs}$ increase, the $\:\varDelta\:P-Q$ curves shift uniformly upward, and hence the pressure increases steadily across the channel at all values of $\:\gamma\:$. On the other hand, in the case of $\:\beta\:$ and $\:{\varphi\:}_{1}$ (Fig. 8b,d), the $\:\varDelta\:P-Q$ curves are mostly linear with certain peculiarities: they seem to decrease in magnitude at certain intervals, but the eventual effect of increasing these parameters is to increase the difference in pressure. Furthermore, the variation among parameter values at a constant $\:\gamma\:$ is minimal at the center of the channel, but it becomes more significant near the walls, indicating a greater influence of the parameters in areas with higher shear.

The higher the $\:\gamma\:$, the lower the $\:\varDelta\:P-Q$ curves will be, indicating that it takes a lesser pressure difference to maintain the same flow rate $\:Q$. Although the local magnitudes of the fluid velocities near the cilia decrease as $\:\gamma\:$ increases, the coordinated action of the longer cilia produces a metachronal pattern of waves that increases the total efficiency of the transport. This is due to the fact that longer cilia generate more effective metachronal waves, which increase the level of fluid propulsion as a result of the coordination of the movement of the flows along the channel surface. The synchronized wave motion reduces viscous drag and eliminates flow recirculation, which typically results in energy loss. Therefore, even though the instantaneous velocity is lower in the areas around the tips of the cilia, the transport of the fluid in the channel is smoother and more efficient. The increased transport decreases the viscous resistance in the channel, thus it takes a smaller external pressure to attain the same volumetric flow rate.

### Skin friction

**Fig. 9:**
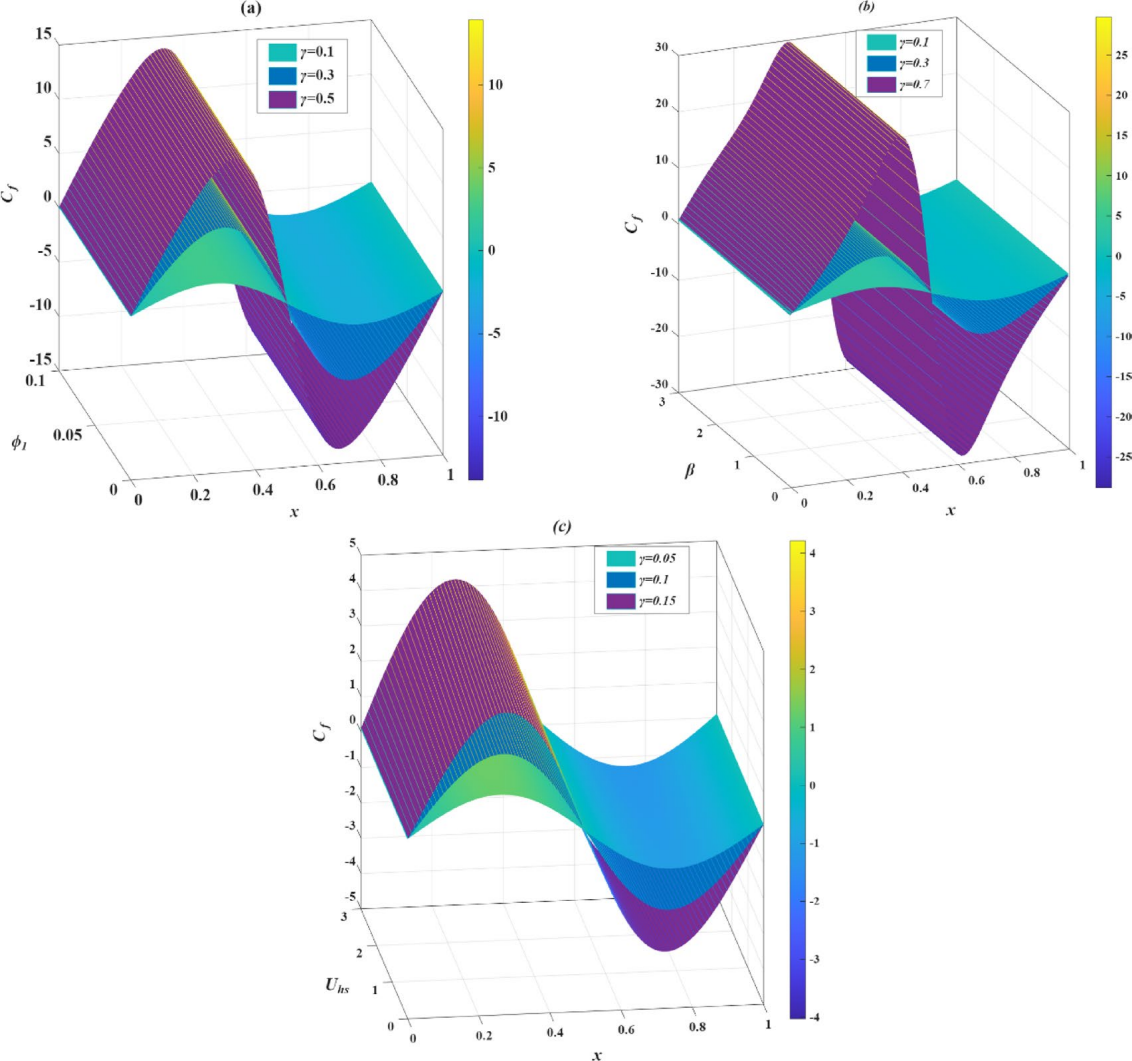
Variation of skin friction coefficient ($\:{C}_{f}$) with cilia lengths ($\:\gamma\:$) across different flow parameters: (**a**) viscosity variation parameter ($\:{\phi\:}_{1}$), (**b**) Casson fluid parameter ($\:\beta\:$), and (**c**) Helmholtz-Smoluchowski velocity ($\:{U}_{hs}$).

The skin friction coefficient, defined in terms of wall shear stress, is analyzed through 3D surface plots as depicted in Fig. 9a–c, illustrating its variation with respect to the parameters. The analysis reveals a distinct periodic behavior in the skin friction distribution, with the profiles exhibiting a wave-like structure along the channel length. It is observed that the skin friction coefficient becomes zero at the extreme points of contraction and expansion, corresponding to locations where the wall shear stress vanishes due to the reversal of the cilia tip motion near the wall.

In Fig. 9a–c, the skin friction coefficient $\:{C}_{f}$ is shown in relation to the temperature-dependent viscosity parameter $\:{(\phi\:}_{1})$, Casson fluid parameter $\:\left(\beta\:\right)$, Helmholtz–Smoluchowski velocity ($\:{U}_{hs}$), and axial position ($\:x$), demonstrating a nonlinear relationship along the length of the channel. At lower values of these parameters, $\:{C}_{f}$ rises along the channel, particularly near the inlet, due to increased wall shear stress that results from higher fluid viscosity, reduced yield stress, or amplified electroosmotic flow, respectively. As these parameters increase further, $\:{C}_{f}\:$begins to drop downstream. Additionally, longer cilia exhibit a more significant variation in skin friction overall, with the graphs displaying both higher maxima and lower minima compared to those of shorter cilia.

### Nusselt number

**Fig. 10:**
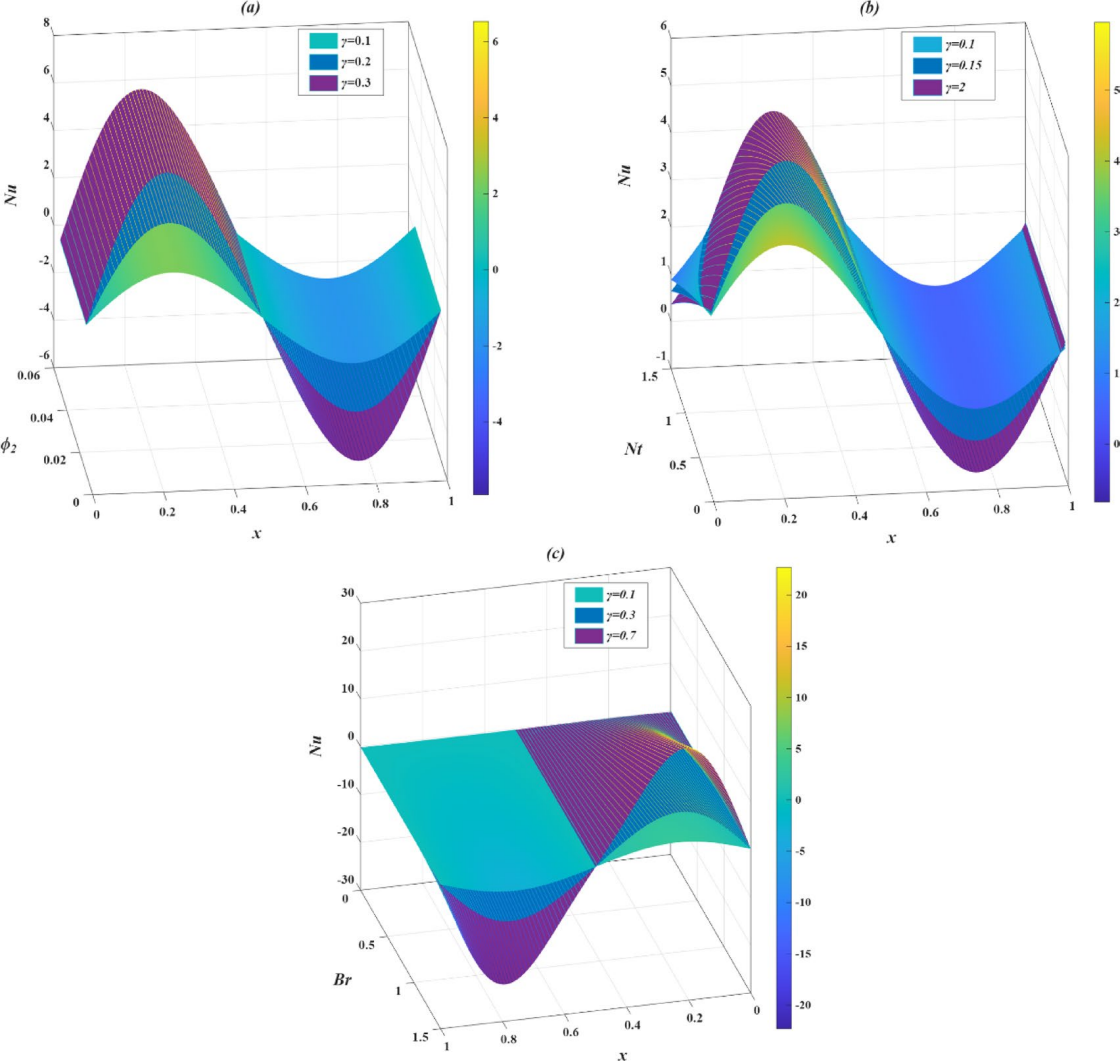
Variation of Nusselt number (*Nu*) with cilia lengths ($\:\gamma\:$) across different parameters: (**a**) thermal conductivity variation parameter ($\:{\phi\:}_{2}$), (**b**) thermophoresis parameter ($\:Nt$), and (**c**) Brinkman number ($\:Br$).

This section provides an in-depth examination of the local Nusselt number $\:Nu$, which indicates the ratio of convective to conductive heat transfer at the channel boundary, thus acting as a crucial metric for assessing thermal transport efficiency within the microchannel. By altering $\:\gamma\:$, the analysis captures the influence of cilia-induced flow and surface interactions on thermal gradients. Figure 10a–c illustrates various thermophysical parameters, all of which significantly affect thermal behavior in nanofluidic and electroosmotic systems.

In Fig. 10a,b, the parameters for thermal conductivity $\:{\phi\:}_{2}$ and thermophoresis $\:Nt$ display analogous behavior concerning the Nusselt number $\:Nu$. As these parameters increase, $\:Nu$ initially grows in the upstream section due to improved conduction and thermophoretic actions that narrow the thermal boundary layer, thereby enhancing heat transfer close to the wall. However, downstream, $\:Nu$ experiences a decline as the thermal boundary layer becomes thicker. This effect becomes more pronounced with longer cilia, which intensifies both the peaks and troughs of $\:Nu$, emphasizing the contribution of extended cilia in boosting convective heat transfer and thermal dispersion. On the other hand, Fig. 10c demonstrates the impact of the Brinkman number $\:Br$, which shows a different pattern. An increase in $\:Br$ leads to a notable reduction in $\:Nu$, independent of cilia length, because viscous dissipation acts as an internal heat source that diminishes the thermal gradient near the wall and obstructs heat transfer. In this scenario, a rise in $\:\gamma\:$ does not mitigate the decline, as the effects of dissipation prevail and lessen the advantages of cilia.

### Sherwood number

**Fig. 11:**
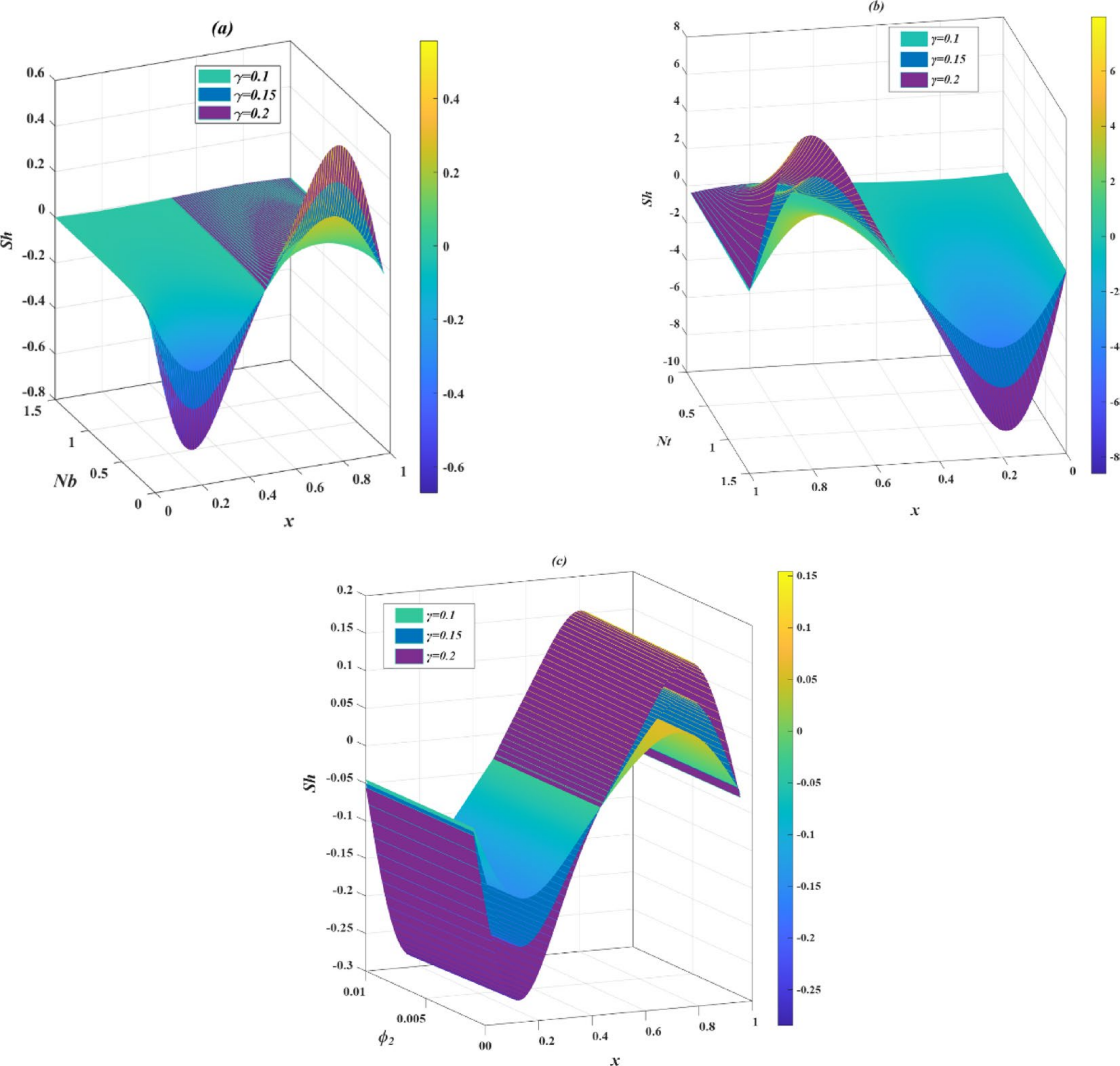
Variation of Sherwood number (*Sh*) with cilia lengths ($\:\gamma\:$) across different parameters: (**a**) Brownian motion parameter ($\:Nb$), (**b**) thermophoresis parameter ($\:Nt$), and (**c**) thermal conductivity variation parameter ($\:{\phi\:}_{2}$).

The Sherwood number $\:\left(Sh\right)$ is a dimensionless metric that indicates the rate of mass transfer at the wall of a channel, represented as the ratio of solute transport by convection to that by diffusion. A higher $\:Sh$ indicates stronger concentration gradients and more effective exchange of solutes between the wall and the fluid. Figure 11a–f investigates the axial variation of $\:Sh$ under the influence of several key parameters. Each graph analyzes how the parameters affect $\:Sh$ for three distinct values of the cilia length parameter $\:\gamma\:$, which influences the flow intensity induced by the cilia on the microchannel surfaces. The behavior of $\:Sh$ is illustrated using 3D surface plots, which demonstrate the progression of solute transport along the axial coordinate $\:x$ and in relation to each parameter. These surfaces feature contour lines that link points of equal $\:Sh$ value and act as visual aids for identifying gradients and peaks throughout the domain.

In Fig. 11a, the Sherwood number $\:Sh$ displays a non-linear relationship with the Brownian motion parameter $\:Nb$, initially declining close to the inlet as enhanced nanoparticle diffusion diminishes concentration gradients at the wall, followed by an increase downstream as other transport processes start to dominate. Extended cilia amplify this phenomenon by further disrupting the concentration boundary layer. Likewise, Fig. 11b illustrates that an increase in the thermophoresis parameter $\:Nt$ initially results in a rise in $\:Sh$. As particles migrate away from the heated surface, which increases the mass flux, before decreasing downstream due to a lower solute concentration near the wall, this trend becomes more pronounced with intensified ciliary activity. In Fig. 11c, variations in the thermal conductivity parameter $\:{\phi\:}_{2}$ produce an alternating pattern in $\:Sh$, which first decreases and then rises along the axial direction, reflecting the indirect influence of thermal variations on mass transport. These changes become more evident with longer cilia lengths, highlighting the combined effects of thermal conductivity and cilia movement.

### Trapping phenomena

**Fig. 12:**
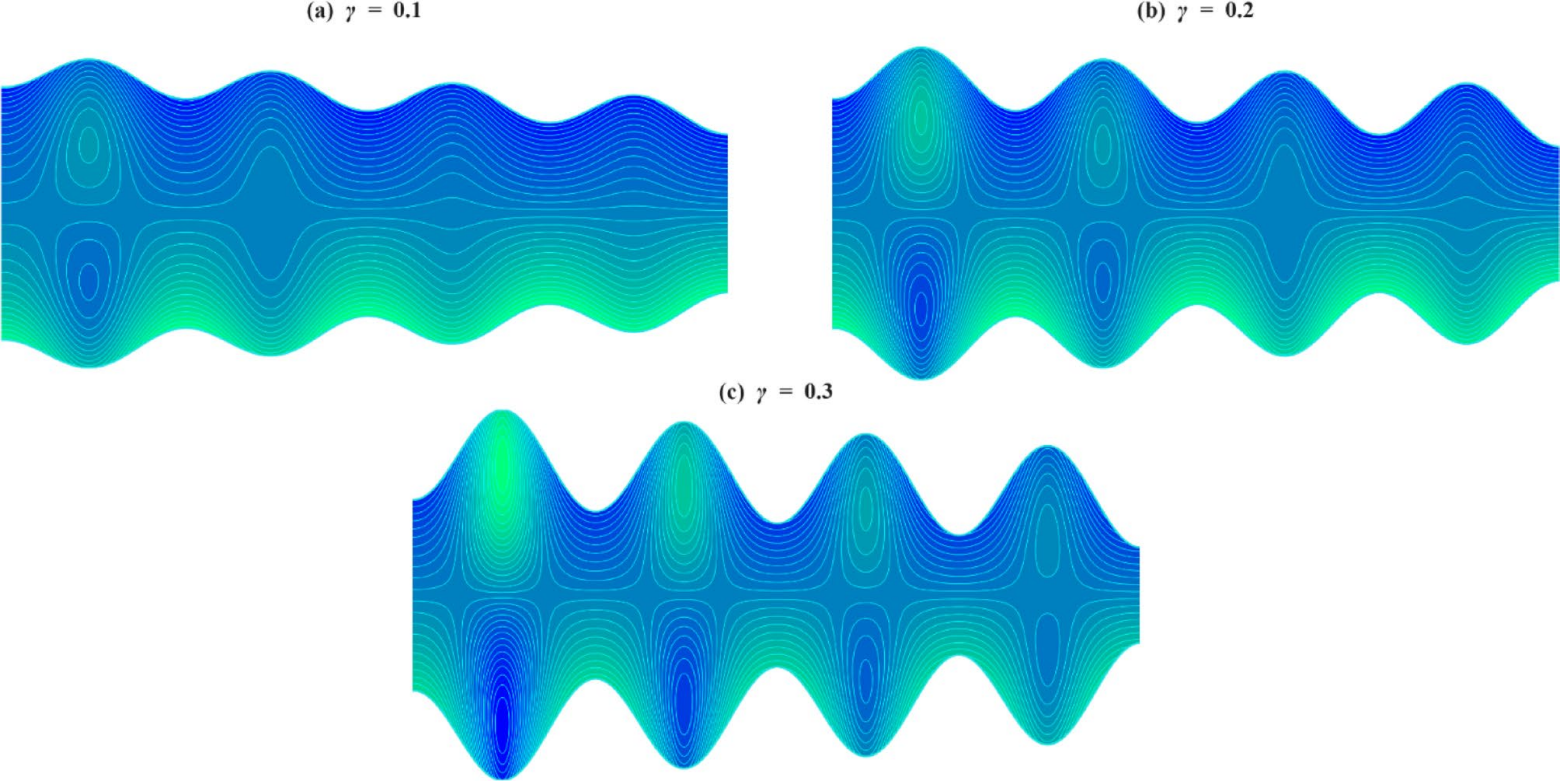
Contour plots of the stream function for varying cilia length.

**Fig. 13:**
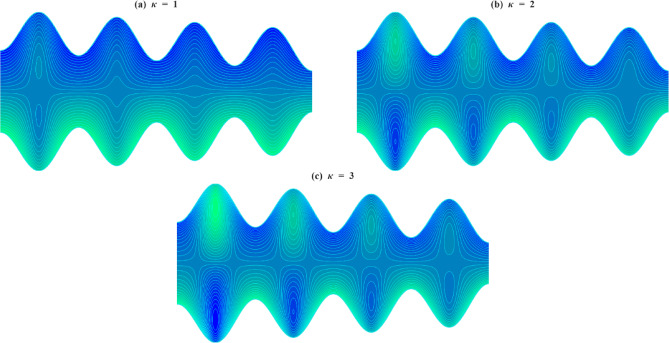
Contour plots of the stream function for varying electro-osmotic parameter.

**Fig. 14:**
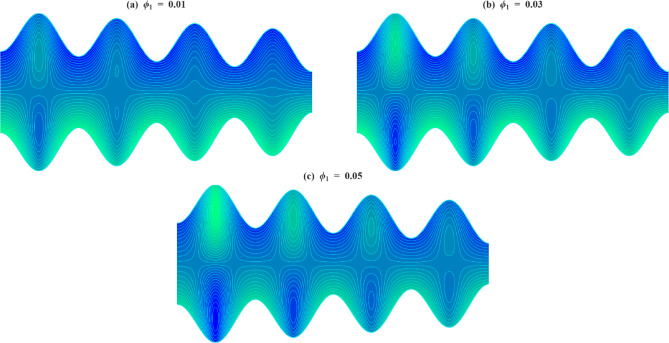
Contour plots of the stream function for a variable viscosity parameter.

Figure 12a–c shows how the flow pattern changes considerably with the increase of the cilia length. When the cilia are smaller ($\:\gamma\:$ = 0.1), the streamlines follow a straight direction along the channel, signalling a smooth forward flow. Small boluses start to form around the walls as the cilia attain medium length ($\:\gamma\:$ = 0.2) and become bigger and further spread into the channel as the cilia become longer ($\:\gamma\:$ = 0.3). These boluses are regions through which the fluid rotates relative to each other; hence, less fluid moves in a straight line. Therefore, the average velocity of the fluid moving in the channel reduces. However, the longer cilia work better to create a synchronized metachronal wave, a type of moving wave along the wall that actively causes the fluid to move. Hence, although a portion of energy is spent on such vortex movements along the walls, the pumping effect of the cilia is used to create a smoother fluid flow along the channel. Therefore, longer cilia make the flow around the walls more complicated; yet, they also behave as a more efficient internal pump, which requires less external pressure to ensure the movement of fluids. Figure 13a–c shows the effect of the electroosmotic parameter $\:\kappa\:$ on the trapping action in the ciliated microchannel. With an increase in $\:\kappa\:$, the closeness boluses and sharpness are enhanced, implying a stronger propulsion of electroosmotic near the walls. These magnified vortex formations imply increased trapping and flow circulation that is powered by the electric field. The confined zones also become larger and thicker, especially in the middle of the section, which highlights the importance of $\:\kappa\:$ as a modulator of intra-channel flow dynamics. Figure 14a–c illustrates the influence of the parameter of viscosity (temperature-dependent) $\:{\phi\:}_{1}$ on the structure of the flow field in a converging ciliated microchannel. At $\:{\phi\:}_{1}=0.01$, the streamlines are slightly distorted and are symmetrically displayed as closed bolus-like circulations along the upper and lower walls. At $\:{\phi\:}_{1}=0.03$, the recirculating regions are further highlighted and marginally displaced downstream, indicating an increase in regional viscous resistance and an altered influence on wall shear stress. At $\:{\phi\:}_{1}=0.05$, the bolus formations are sharper and more compressed, and the number and intensity of the bolus are slightly higher. This effect means that with increasing viscosity, the localization of flow at the boundaries increases, and the curvature of streams is altered. However, the converging geometry imposes a natural limit on how much the streams can be expanded in the center.

.

## Conclusion

### Summary and key findings

This study presents a mathematical model of non-Newtonian Buongiorno nanofluid flow in an inclined, ciliated microchannel. The formulation accounts for cilia length, electroosmotic effects, Casson fluid rheology, viscous heating, and variable thermal properties. The governing equations were solved using the Homotopy Perturbation Method (HPM) up to the second order. The main findings are summarized below:

- The variation in cilia length ($\:\gamma\:$) plays a crucial role in controlling the flow behavior within the microchannel. Increasing cilia length reduces the axial velocity and flow rate due to enhanced wall resistance, while also lowering fluid temperature by restricting convective transport. At the same time, longer cilia promote nanoparticle trapping near the walls, which improves pumping performance and enables precise regulation of microscale flow.
- The electroosmotic parameter ($\:\kappa\:$) enhances flow velocity and reduces the required pressure gradient by counteracting the drag induced by cilia motion. This coupling of electroosmotic forces and ciliary propulsion significantly improves fluid transport efficiency in low Reynolds number conditions.
- Variable viscosity ($\:{\phi\:}_{1}$) and thermal conductivity ($\:{\phi\:}_{2}$) capture realistic microscale effects, showing that higher temperature dependence increases velocity and heat transfer while reducing flow resistance. These variations better represent biological and microfluidic environments where fluid properties change with temperature.
- The Brownian motion parameter ($\:Nb$) increases both temperature and nanoparticle concentration due to stronger particle diffusion, while the thermophoresis parameter ($\:Nt$) drives nanoparticles away from heated regions, lowering their concentration and temperature near the walls. These combined effects influence the nature of heat and mass transfer in confined microscale geometries.

### Limitations and assumptions

In this study, the Debye–Hückel approximation is employed to linearize the Poisson-Boltzmann equation, which is valid for low zeta potentials. However, this limits its applicability to systems with weak electrokinetic interactions, as higher potentials would require solving the full nonlinear equation. The lubrication approximation is adopted to simplify the governing equations by neglecting inertial effects, which is appropriate for low Reynolds number microflows but restricts the analysis to steady, laminar regimes. The Casson model is used to represent the yield-stress behavior of non-Newtonian biological fluids, such as blood and mucus; however, it does not account for viscoelastic or time-dependent effects that may occur in real physiological conditions.

### Future scope

Future research could expand on this framework by integrating more complexities related to physical and physiological aspects. One potential avenue is to incorporate electro-magneto-hydrodynamic effects by adding Lorentz force terms, which would enhance our understanding of transport behavior in the presence of simultaneous electric and magnetic fields. Moreover, investigating curved, branching, or irregular microchannel configurations can more closely mimic the structure of biological microvessel networks. Another potential extension is to simulate nonlinear electrochemical surface reactions, which are important for bio-electrochemical sensing and precision therapeutic systems. To more accurately represent actual biological flow dynamics, the model could also integrate transient and pulsatile flow to reflect cardiovascular behaviors under varying time-dependent conditions. Lastly, connecting the system with fluid–structure interaction (FSI) in COMSOL would enable the examination of how endothelial walls respond to electroosmotic flow and ciliary activity. These improvements would enhance our comprehension of microcirculatory processes and aid in the development of advanced electro-therapeutic, diagnostic, and nanomedicine delivery systems.

## Supplementary Information

Below is the link to the electronic supplementary material.

Supplementary Material 1

## Data Availability

The datasets used and/or analysed during the current study available from the corresponding author on reasonable request.

## Appendix

$$\begin{gathered} a_{1} = - \frac{1}{{1 - K_{{nu}} \delta - \delta \gamma b_{1} \alpha _{0} 2\pi \cos \left( {2\pi x} \right)}},\,a_{2} = \frac{1}{2}\left( {2a_{1} + h^{2} } \right), \hfill \\ a_{3} = \frac{1}{2}\left( {2 - h^{2} } \right),\,a_{4} = \frac{1}{2}\left( {2 + h^{2} } \right),\,a_{5} = \frac{{Gr}}{{\left( {1 + \frac{1}{\beta }} \right)}}, \hfill \\ \end{gathered}$$

$$\begin{gathered} a_{6} = \frac{{Gm}}{{\left( {1 + \frac{1}{\beta }} \right)}},\,a7 = \frac{{\frac{{\text{Sin} \left[ \alpha \right]}}{F}}}{{\left( {1 + \frac{1}{\beta }} \right)}} - \frac{P}{{\left( {1 + \frac{1}{\beta }} \right)}}, \hfill \\ a_{8} = \frac{{\left( {\kappa ^{2} U_{{hs}} } \right)}}{{\left( {1 + \frac{1}{\beta }} \right)\cosh \left[ {\kappa h} \right]}},\,a_{9} = \frac{{a_{5} h^{2} }}{2} - \frac{{5a_{5} h^{4} }}{{24}} - \frac{{a_{6} h^{4} }}{{24}}, \hfill \\ \end{gathered}$$

$$\begin{gathered} a_{{10}} = \left( { - \frac{{a_{5} }}{{24}} + \frac{{a_{6} }}{{24}} - \frac{{\phi _{1} }}{8}} \right),\,a_{{11}} = - \frac{{h^{4} \phi _{1} }}{8} + \frac{1}{2}h^{2} \left( { - 1 + a_{4} a_{6} + a_{7} + \phi _{1} } \right), \hfill \\ a_{{12}} = \left( { - \frac{{a_{5} }}{2} + \frac{{a_{5} h^{2} }}{4} + \frac{1}{2}\left( {1 - a_{4} a_{6} - a_{7} - \phi _{1} } \right) + \frac{{h^{2} \phi _{1} }}{4}} \right),\,a_{{13}} = \frac{{a_{8} \cosh \left[ {h\kappa } \right]}}{{\kappa ^{2} }}, \hfill \\ \end{gathered}$$

$$\begin{gathered} a_{{14}} = \frac{{a_{8} }}{{\kappa ^{2} }},\,a_{{15}} = Br\left( {1 + \frac{1}{\beta }} \right),\,a_{{16}} = \frac{1}{{60}}a_{{15}} \phi _{1} , \hfill \\ a_{{17}} = \frac{1}{{120}}\left( { - 10a_{{15}} + 10Nb - 10Nt + 10a_{{15}} \phi _{1} - 5a_{{15}} h^{2} \phi _{1} - 15\phi _{2} } \right), \hfill \\ a_{{18}} = \frac{1}{{120}}\left( { - 60 - 60\phi _{2} + 30h^{2} \phi _{2} } \right), \hfill \\ \end{gathered}$$

$$\begin{gathered} a_{{19}} = \frac{1}{{120}}(60h^{2} + 10a_{{15}} h^{4} - 10h^{4} Nb + 10h^{4} Nt - 10a_{{15}} h^{4} \phi _{1} \hfill \\ + 3a_{{15}} h^{6} \phi _{1} + 60h^{2} \phi _{2} - 15h^{4} \phi _{2} ),\,a_{{20}} = \frac{{Nt}}{{Nb}},\,a_{{21}} = \frac{1}{2}\left( { - 1 + a_{{20}} } \right)h^{2} , \hfill \\ a_{{22}} = \frac{{\left( { - 15a_{{16}} a_{5} \kappa ^{2} - 105a_{{16}} \kappa ^{2} \phi _{1} } \right)}}{{840\kappa ^{2} }}, \hfill \\ \end{gathered}$$

$$\begin{gathered} a_{{23}} = \frac{{\left( { - 28a_{{17}} a_{5} \kappa ^{2} + 280a_{{10}} \kappa ^{2} \phi _{1} - 140a_{{17}} \kappa ^{2} \phi _{1} } \right)}}{{840\kappa ^{2} }},\,a_{{24}} = \hfill \\ \frac{{\left( { - 70a_{{18}} a_{5} \kappa ^{2} + 70a_{{21}} a_{6} \kappa ^{2} + 840a_{{10}} \kappa ^{2} \phi _{1} + 210a_{{12}} \kappa ^{2} \phi _{1} - 210a_{{18}} \kappa ^{2} \phi _{1} - 420a_{{10}} h^{2} \kappa ^{2} \phi _{1} } \right)}}{{840\kappa ^{2} }}, \hfill \\ \end{gathered}$$

$$\begin{gathered} a_{{25}} = \frac{{\left( { - 420a_{{19}} a_{5} \kappa ^{2} - 420a_{{21}} a_{6} \kappa ^{2} + 840a_{{12}} \kappa ^{2} \phi _{1} - 420a_{{19}} \kappa ^{2} \phi _{1} - 420a_{{12}} h^{2} \kappa ^{2} \phi _{1} } \right)}}{{840\kappa ^{2} }}, \hfill \\ a_{{26}} = \frac{{\left( { - 420a_{{14}} \kappa ^{2} \phi _{1} } \right)}}{{840\kappa ^{2} }},\,a_{{27}} = \frac{1}{{840\kappa ^{2} }}(420a_{{19}} a_{5} h^{2} \kappa ^{2} + 420a_{{21}} a_{6} h^{2} \kappa ^{2} + 70a_{{18}} a_{5} h^{4} \kappa ^{2} \hfill \\ - 70a_{{21}} a_{6} h^{4} \kappa ^{2} + 28a_{{17}} a_{5} h^{6} \kappa ^{2} + 15a_{{16}} a_{5} h^{8} \kappa ^{2} - 840a_{{12}} h^{2} \kappa ^{2} \phi _{1} + 420a_{{19}} h^{2} \kappa ^{2} \phi _{1} \hfill \\ - 840a_{{10}} h^{4} \kappa ^{2} \phi _{1} + 210a_{{12}} h^{4} \kappa ^{2} \phi _{1} + 210a_{{18}} h^{4} \kappa ^{2} \phi _{1} + 140a_{{10}} h^{6} \kappa ^{2} \phi _{1} \hfill \\ + 140a_{{17}} h^{6} \kappa ^{2} \phi _{1} + 105a_{{16}} h^{8} \kappa ^{2} \phi _{1} + 840a_{{14}} \phi _{1} \cosh \left[ {h\kappa } \right] \hfill \\ + 840a_{{14}} \kappa ^{2} \phi _{1} \cosh \left[ {h\kappa } \right] - 840a_{{14}} h\kappa \phi _{1} \sinh \left[ {h\kappa } \right]), \hfill \\ \end{gathered}$$

$$\begin{gathered} a_{{28}} = \frac{1}{{840\kappa ^{2} }}*\left( { - 840a_{{14}} \phi _{1} - 840a_{{14}} \kappa ^{2} \phi _{1} + 420a_{{14}} h^{2} \kappa ^{2} \phi _{1} } \right), \hfill \\ a_{{29}} = \frac{{a_{{14}} \phi _{1} }}{\kappa },\,a_{{30}} = \frac{1}{{90}}a_{{15}} a_{{16}} \phi _{1} ,\,a_{{31}} = \hfill \\ \frac{{\left( {270a_{{16}} Nb\kappa ^{4} - 540a_{{16}} Nt\kappa ^{4} - 180a_{{10}} a_{{15}} \kappa ^{4} \phi _{1} + 45a_{{15}} a_{{17}} \kappa ^{4} \phi _{1} - 1260a_{{16}} \kappa ^{4} \phi _{2} } \right)}}{{2520\kappa ^{4} }}, \hfill \\ \end{gathered}$$

$$\begin{gathered} a_{{32}} = \frac{1}{{2520\kappa ^{4} }}(672a_{{10}} a_{{15}} \kappa ^{4} + 336a_{{17}} Nb\kappa ^{4} - 672a_{{17}} Nt\kappa ^{4} \hfill \\ - 672a_{{10}} a_{{15}} \kappa ^{4} \phi _{1} - 168a_{{12}} a_{{15}} \kappa ^{4} \phi _{1} + 84a_{{15}} a_{{18}} \kappa ^{4} \phi _{1} \hfill \\ + 336a_{{10}} a_{{15}} h^{2} \kappa ^{4} \phi _{1} - 2520a_{{16}} \kappa ^{4} \phi _{2} - 1260a_{{17}} \kappa ^{4} \phi _{2} \hfill \\ + 1260a_{{16}} h^{2} \kappa ^{4} \phi _{2} ),\,a_{{33}} = \frac{1}{{2520\kappa ^{4} }}(840a_{{12}} a_{{15}} \kappa ^{4} \hfill \\ + 420a_{{18}} Nb\kappa ^{4} + 420a_{{21}} Nb\kappa ^{4} - 840a_{{18}} Nt\kappa ^{4} - 840a_{{12}} a_{{15}} \kappa ^{4} \phi _{1} \hfill \\ + 210a_{{15}} a_{{19}} \kappa ^{4} \phi _{1} + 420a_{{12}} a_{{15}} h^{2} \kappa ^{4} \phi _{1} - 2520a_{{17}} \kappa ^{4} \phi _{2} \hfill \\ - 1260a_{{18}} \kappa ^{4} \phi _{2} + 1260a_{{17}} h^{2} \kappa ^{4} \phi _{2} ), \hfill \\ \end{gathered}$$

$$\begin{gathered} a_{{34}} = \frac{{\left( { - 2520a_{{18}} \kappa ^{4} \phi _{2} - 1260a_{{19}} \kappa ^{4} \phi _{2} + 1260a_{{18}} h^{2} \kappa ^{4} \phi _{2} } \right)}}{{2520\kappa ^{4} }}, \hfill \\ a_{{35}} = \frac{{\left( { - 15120a_{{14}} a_{{15}} \kappa ^{2} \phi _{1} } \right)}}{{2520\kappa ^{4} }},\,a_{{36}} = \frac{1}{{2520\kappa ^{4} }}( - 840a_{{12}} a_{{15}} h^{4} \kappa ^{4} \hfill \\ - 672a_{{10}} a_{{15}} h^{6} \kappa ^{4} - 420a_{{18}} h^{4} Nb\kappa ^{4} - 420a_{{21}} h^{4} Nb\kappa ^{4} - \hfill \\ 336a_{{17}} h^{6} Nb\kappa ^{4} - 270a_{{16}} h^{8} Nb\kappa ^{4} + 840a_{{18}} h^{4} Nt\kappa ^{4} \hfill \\ + 672a_{{17}} h^{6} Nt\kappa ^{4} + 540a_{{16}} h^{8} Nt\kappa ^{4} + 840a_{{12}} a_{{15}} h^{4} \kappa ^{4} \phi _{1} - \hfill \\ 210a_{{15}} a_{{19}} h^{4} \kappa ^{4} \phi _{1} + 672a_{{10}} a_{{15}} h^{6} \kappa ^{4} \phi _{1} - 252a_{{12}} a_{{15}} h^{6} \kappa ^{4} \phi _{1} - \hfill \\ 84a_{{15}} a_{{18}} h^{6} \kappa ^{4} \phi _{1} - 156a_{{10}} a_{{15}} h^{8} \kappa ^{4} \phi _{1} - 45a_{{15}} a_{{17}} h^{8} \kappa ^{4} \phi _{1} \hfill \\ - 28a_{{15}} a_{{16}} h^{{10}} \kappa ^{4} \phi _{1} + 2520a_{{18}} h^{2} \kappa ^{4} \phi _{2} + 1260a_{{19}} h^{2} \kappa ^{4} \phi _{2} \hfill \\ + 2520a_{{17}} h^{4} \kappa ^{4} \phi _{2} + 2520a_{{16}} h^{6} \kappa ^{4} \phi _{2} - 10080a_{{14}} a_{{15}} \kappa ^{2} \cosh \left[ {h\kappa } \right] \hfill \\ + 60480a_{{14}} a_{{15}} \phi _{2} \cosh \left[ {h\kappa } \right] + 10080a_{{14}} a_{{15}} \kappa ^{2} \phi _{1} \cosh \left[ {h\kappa } \right] + \hfill \\ 10080a_{{14}} a_{{15}} h^{2} \kappa ^{2} \phi _{1} \cosh \left[ {h\kappa } \right] + 5040a_{{14}} a_{{15}} h\kappa ^{3} \sinh \left[ {h\kappa } \right] \hfill \\ - 45360a_{{14}} a_{{15}} h\kappa \phi _{1} \sinh \left[ {h\kappa } \right] - 5040a_{{14}} a_{{15}} h\kappa ^{3} \phi _{1} \sinh \left[ {h\kappa } \right]), \hfill \\ \end{gathered}$$

$$\begin{gathered} a_{{37}} = \frac{1}{{2520\kappa ^{4} }}*( + 10080a_{{14}} a_{{15}} \kappa ^{2} - 60480a_{{14}} a_{{15}} \phi _{1} - \hfill \\ 10080a_{{14}} a_{{15}} \kappa ^{2} \phi _{1} + 5040a_{{14}} a_{{15}} h^{2} \kappa ^{2} \phi _{1} ),\,a_{{38}} = \frac{{a_{{14}} a_{{15}} \phi _{1} }}{\kappa },\,a_{{39}} = \hfill \\ \frac{{\left( { - 5040a_{{14}} a_{{15}} \kappa ^{3} + 45360a_{{14}} a_{{15}} \kappa \phi _{1} + 5040a_{{14}} a_{{15}} \kappa ^{3} \phi _{1} - 2520a_{{14}} a_{{15}} h^{2} \kappa ^{3} \phi _{1} } \right)}}{{2520\kappa ^{4} }}, \hfill \\ a_{{40}} = a_{{18}} a_{{20}} h^{2} + a_{{17}} a_{{20}} h^{4} + a_{{16}} a_{{20}} h^{6}. \hfill \\ \end{gathered}$$

## Abbreviations

${y}{\prime}$, $y$: Dimensional and dimensionless transverse coordinate
${H}{\prime}$, $h$: Dimensional and dimensionless ciliary microtube wall
${t}{\prime}$, $t$: Dimensional and dimensionless time
${a}_{1}$: Average ciliated channel radius
${\beta }_{0}$, ${K}_{nu}$: Dimensional and dimensionless non uniformity factor
${x}{\prime}$, $x$: Dimensional and dimensionless axial coordinate
$$c$$: Wave speed
$$\gamma$$: Cilia length
$${a}^{*}$$: Wave amplitude
$$\lambda$$: Wavelength
${{x}_{0}}{\prime}$: Position of reference particle
${\alpha }_{0}$: Eccentricity of elliptical orbit
${W}{\prime}, {V}{\prime}$: Cilia tip velocity components
${w}{\prime}$, $w$: Dimensional and dimensionless velocity component along axial direction
${v}{\prime}$, $v$: Dimensional and dimensionless velocity component along transverse direction
${p}{\prime}$, $P$: Dimensional and dimensionless pressure
$${{\tau }{\prime}}_{ij}$$: Stress tensor
${C}_{0}, {C}_{1}$: Nanoparticle volume fraction at reference (cold) and hot boundary
$${T}_{0}$$: Wall temperature
${\rho }_{c}$: Density of the nanoparticle material
${\beta }_{C}$: Concentration expansion coefficient
${C}{\prime}$, $\xi$: Dimensional and dimensionless concentration
$\rho$: Density of the fluid
$$\alpha$$: Inclination angle
${c}_{p}$: Specific heat capacity at constant pressure
${E}_{x}$: Axial electric body field
${E}_{y}$: Transverse electric body field
${U}_{hs}$: Helmholtz-Smoluchowski parameter
$$Fr$$: Froude number
$$Br$$: Brinkman number
$$\kappa$$: Electroosmosis factor
${b}_{0}$: Width of the channel
$${\lambda }_{D}$$: Debye length
$${b}_{1}$$: Amplitude ratio
$Nb$: Brownian motion parameter
$\overline{K }\left({T}{\prime}\right)$,$K\left(\theta \right)$: Dimensional and dimensionless variable thermal conductivity
$$Nt$$: Thermophoresis parameter
${c}_{f}$: Specific heat capacity of the base fluid at constant pressure
$Gr$: Thermal Grashof number
$Gm$: Mass Grashof number
${\phi }_{1}$: Variable viscosity parameter
${\phi }_{2}$: Variable thermal conductivity parameter
${\rho }_{f}$: Density of the base fluid
$g$: Acceleration due to gravity
${\beta }_{T}$: Volumetric thermal expansion coefficient
${T}{\prime}$, $\theta$: Dimensional and dimensionless temperature
${\rho }_{f}$: Density of the base fluid
${D}_{B}$: Brownian diffusion coefficient
${D}_{T}$: Themophoretic diffusion coefficient
${T}_{b}$: Reference (bulk) temperature
$\overline{\mu }\left({T}{\prime}\right)$, $\mu \left(\theta \right)$: Dimensional and dimensionless variable viscosity with respect to temperature
$\beta$: Casson fluid parameter
$$\delta$$: Wave number
$$Re$$: Reynolds number
$F$: Body force parameter
${\rho }_{e}$: Denotes the total charge density per unit volume
