# Supplementary material for: Impact of cilia length and variable fluid properties on electroosmotic nanofluid flow in an inclined converging microchannel
Source: Sci Rep. 2025 Dec 13;16:1113. doi: 10.1038/s41598-025-30669-z (PMC12789520; doi:10.1038/s41598-025-30669-z)
Supplement: Supplementary file 1 — Supplementary Material 1 [file 41598_2025_30669_MOESM1_ESM.docx]

**Impact of Cilia Length and Variable Fluid Properties on Electroosmotic Nanofluid Flow in an Inclined Converging Microchannel**

In this supplementary text, we provide a detailed explanation of electroosmotic flow in micro- and nanoscale systems, focusing on the influence of temperature-dependent viscosity and thermal conductivity. The discussion also covers Nanofluids and the Buongiorno Model, along with additional results and graphical interpretations for enhanced clarity.

1. **Extended Background:**

**Electroosmotic Flow in Micro and Nanoscale Systems:**

Electro-osmosis is a key process of fluid motion in micro- and nanoscale systems, caused by the application of an external electric field across an electrolyte-filled conduit with charged walls. When a polar surface interacts with an electrolyte, counter-ions aggregate in the vicinity of the surface, resulting in the formation of an electric double layer (EDL). This layer is composed of a compact Stern layer and a mobile diffuse layer. The application of an external electric field causes the mobile ions within the diffuse layer to move, consequently pulling along the adjacent fluid and generating bulk flow through viscous effects. This process is referred to as electroosmotic flow (EOF) [1]. Yao et al. adjusted the electroosmotic flow (EOF) in capillaries coated with surfactants and polymers to regulate flow characteristics, whereas Yang et al. noted the presence of separation bubbles at 90° junctions in microchannels operating at high Reynolds numbers, demonstrating the dependence of EOF on geometric configurations [2]. Ghosh and Chakraborty [3] took a deeper look at the electroosmotic flow of viscoelastic fluids by applying the thin EDL approximation. In the realm of biological and lab-on-a-chip systems, Li and Harrison [4] illustrated the application of electrokinetic effects for the transportation, trapping, and reaction of biological cells on-chip. Subsequently, Gillespie and Pennathur [5] demonstrated that electroosmosis can improve ionic separation when it is applied in opposition to pressure-driven flow. Chakraborty [6] theoretically shown that electroosmotic actuation may greatly increase the volume flow rate in a peristaltic transport arrangement with an applied axial electric field using a long-wave approximation. The integration of electroosmosis and peristalsis is especially beneficial for replicating physiological flow conditions, which is crucial in applications like tissue engineering and targeted drug delivery. Additionally, electroosmotic pumping has been successfully utilized in biomicrofluidic devices, including silicon-based micropumps and systems that utilize electrostatic or thermopneumatic actuation for flow regulation [7]. These findings are in line with the respiratory tract's natural mechanism of metachronal ciliary beating, which uses coordinated shear-induced motion to propel mucus. This process, like electroosmosis, depends on near-wall shear stresses and functions at low Reynolds numbers, indicating that electroosmosis may be a biomimetic substitute or an improvement upon ciliary transport [1], [7], [8]. Realistic modelling must take into consideration the fact that such microscale flows are also susceptible to temperature gradients, which cause modifications in viscosity and thermal conductivity.

**Influence of Temperature-Dependent Viscosity and Thermal Conductivity:**

The examination of variable fluid properties, specifically viscosity and thermal conductivity as functions of temperature, is of paramount significance in both biological and engineering contexts. Under actual physiological conditions, particularly in biofluids such as blood and mucus, these properties are not static. Rather, they fluctuate considerably with changes in temperature, concentration, and shear rate, which in turn affects the transport characteristics of fluids within biological channels. For example, viscosity plays a crucial role in the propulsion ability during peristaltic or cilia-driven motion, where a rise in local temperature resulting from internal heat production or metabolic processes can greatly modify fluid resistance and flow dynamics [9] Thermal conductivity is a determinant of the efficiency with which heat is transferred within a fluid medium, playing a vital role in hyperthermia-based therapies and nutrient transport processes [10]. Ignoring the temperature dependency of these properties may result in inaccurate predictions, as illustrated by Elogail and Elshekipy [10], who contrasted constant and variable viscosity models in peristaltic flow, demonstrating that models utilizing constant parameters produce unrealistic results. Likewise, biological research has recognized that bodily fluids like blood or mucus experience changes in viscosity due to temperature shifts caused by hydration levels, health issues, or external heating [11], [12]. Moreover, Jawali et al. [13] emphasized that heat transfer and fluid velocity are quite responsive to these variations, which affects how effectively nutrients are diffused and waste is eliminated across membranes. Since temperature variations across the airway lining can alter mucus rheology, which in turn affects mucociliary clearance and general respiratory health, these changeable qualities become crucial in the context of cilia-driven transport, particularly in the respiratory tract. Recent studies that have modelled blood and nanofluid flow under the effect of temperature-dependent viscosity in biomathematical frameworks, such those by Bhatti et al. [14] have reinforced the necessity of taking these fluctuations into account in realistic models. Consequently, in order to accurately depict physiological behavior and derive more realistic and biologically meaningful insights, variable temperature and viscosity parameters must be included in analytical modeling of cilia-assisted biofluid flows. In order to improve flow control and thermal management, researchers have explored suspensions of nanoparticles, which resulted in the development of nanofluid models.

**Buongiorno Nanofluid Model:**

Nanofluids, which are suspensions of nanoparticles generally measuring between 1 and 100 nm, have shown remarkable potential in improving thermal conductivity and convective heat transfer. As a result, they are now widely used in medicinal devices, improved cooling systems, and energy applications including microreactors and solar collectors [15], [16]. Initial models regarded nanofluids as uniform, single-phase entities with consistent characteristics; however, these simplifications frequently did not align with experimental findings, highlighting the necessity for frameworks that are more aligned with physical principles. Buongiorno's model (2006), which presented a two-component mixture theory that regards the base fluid and nanoparticles as separate phases, represented a major breakthrough. The model enforces no-slip velocity constraints at solid borders for both phases while capturing the relative slip between nanoparticles and the surrounding fluid. By inspecting various slip mechanisms of nanoparticles, Buongiorno concluded that only Brownian motion and thermophoresis are important to the diffusion of nanoparticles. This method accurately simulates how nanoparticles move under changes in concentration and temperature without needing expensive multiphase tracking. This makes it both strong in terms of physics and efficient. Consequently, the model has established itself as a fundamental component in contemporary nanofluid research and has been effectively utilized in various complex flow scenarios, encompassing enhanced convective transfer, flow instability, and magnetohydrodynamic systems [17], [18]. The Buongiorno model, when used for cilia-induced transport, allows us to realistically simulate how nanoparticles behave in mucus or biological fluids by considering both diffusion and thermal effects. This combination is particularly useful for modeling mucociliary clearance, targeted drug delivery, and the dynamics of nanoparticles in the respiratory tract.

1. **Electric Double Layer Formation:**

In electroosmotic flow, the electric double layer (EDL) is formed as a result of interactions between the surface and the electrolyte, particularly at the walls of dielectric channels. For example, in microfluidic systems constructed with silica or glass substrates, the walls develop a net negative surface charge due to the pH-dependent dissociation of silanol groups $\left( \equiv Si-OH \right)$. When these surfaces come into contact with an aqueous electrolyte, some of the silanol groups undergo ionization, resulting in the formation of $\equiv SiO^{-}$ ions and the release of protons into the solution [19]. This ionization phenomenon is particularly pronounced at neutral to basic pH levels, which contributes to a notable negative surface potential.

This surface charge affects the nearby ionic distribution by drawing cations (counter-ions) towards the wall while repelling anions (co-ions), leading to the creation of an electric double layer. The electric double layer consists of a compact layer of adsorbed counter-ions situated near the surface and a diffuse layer where the ionic imbalance gradually decreases. The range of this diffuse region is defined by the Debye length $\lambda_{D}$, which indicates the distance at which the electrostatic potential energy is equal to the thermal energy of the ions.

1. **Boundary Conditions:**

In this microchannel model featuring wall-anchored cilia, we apply the traditional no-slip boundary condition at the fluid–cilia interface. This indicates that the fluid velocity corresponds to the surface velocity of the cilia, resulting in no relative motion between the fluid and the cilia surface. From a physical standpoint, the viscous nanofluid adheres to each cilium: if a cilium remains stationary (for instance, when it is affixed to a fixed wall segment), the fluid adjacent to that surface exhibits zero velocity; conversely, if a cilium is in motion or beats, the fluid at its surface moves in synchrony with that action. This premise is based on the continuum hypothesis and is commonly utilized in microfluidic models that incorporate ciliated geometries, where viscous forces prevail and slippage is minimal on hydrophilic, smooth surfaces [20], [21].

1. **Solution of the Problem Using the Homotopy Perturbation Method (HPM):**

Starting from the governing non-dimensional equations (32–35) presented in the main manuscript, substitution of Eq. (39) into Eq. (32) yields the following reformulated expressions.

$\frac{\partial p}{\partial x}=\left( 1+\frac{1}{\beta} \right)\left( \frac{\partial}{\partial y}\left( 1-\phi_{1}\theta\right)\frac{\partial w}{\partial y} \right)+Gr\theta+Gm\xi+\frac{\sin\alpha}{F}+\kappa^{2}\frac{\cosh(\kappa y)}{\cosh(\kappa h)}U_{hs}$,

$\left( \frac{\partial}{\partial y}\left( 1+\phi_{2}\theta\right)\frac{\partial\theta}{\partial y} \right)+Br\left( 1-\phi_{1}\theta\right)\left( 1+\frac{1}{\beta} \right)\left( \frac{\partial w}{\partial y} \right)^{2}+N_{b}\frac{\partial\xi}{\partial y}\frac{\partial\theta}{\partial y}+N_{t}\left( \frac{\partial\theta}{\partial y} \right)^{2}=0$ ,

$\frac{\partial^{2}\xi}{\partial y^{2}}$+ $\frac{N_{t}}{N_{b}}$ $\frac{\partial^{2}\theta}{\partial y^{2}}=0$ .

By simplifying the above equations we get

$\left. \begin{matrix} \frac{\partial^{2}w}{\partial y^{2}}-\phi_{1}\left( \frac{\partial\theta}{\partial y}\frac{\partial w}{\partial y}+\theta\frac{\partial^{2}w}{\partial y^{2}} \right)+a_{5}\theta+a_{6}\xi+a_{7}+a_{8}\cosh\left( \kappa y \right)=0, \\ \frac{\partial^{2}\theta}{\partial y^{2}}+\phi_{2}\left( \left( \frac{\partial\theta}{\partial y} \right)^{2}+\theta\frac{\partial^{2}\theta}{\partial y^{2}} \right)+a_{15}\left( 1-\phi_{1}\theta\right)\left( \frac{\partial w}{\partial y} \right)^{2}+N_{b}\frac{\partial\xi}{\partial y}\frac{\partial\theta}{\partial y}+N_{t}\left( \frac{\partial\theta}{\partial y} \right)^{2}=0, \\ \frac{\partial^{2}\xi}{\partial y^{2}}+ a_{20} \frac{\partial^{2}\theta}{\partial y^{2}}=0 . \end{matrix} \right\}$ (i)

Where, $a_{5}$, $a_{6}, a_{7}, a_{8}, a_{15}, a_{20}$ are provided in Appendix.

Consider the general expression of the nonlinear differential equation as:

$A\left( u \right)=0$,

where $A$ is a differential operator that can be divided into:

$A\left( u \right)=L\left( u \right)+N(u)$,

with $L$ denoting the linear component and $N$ the nonlinear component of the operator.

A homotopy $H(u,q)$ is then constructed as:

$H\left( u,q \right)=\left( 1-q \right)\left[ L\left( u \right)-L\left( u_{0} \right) \right]+q\left[ A\left( u \right) \right]=0$,

where, $q\in[0,1]$ represents an embedding parameter, $u_{0}$ is an initial approximation that satisfies the boundary conditions.

The homotopy relationships for equations (i) are outlined below for this aim:

$\left. \begin{matrix} H\left( w,q \right)=L\left( w \right)-L\left( w_{0} \right)+qL\left( w_{0} \right)+q\left[ -\phi_{1}\left( \frac{\partial\theta}{\partial y}\frac{\partial w}{\partial y}+\theta\frac{\partial^{2}w}{\partial y^{2}} \right)+a_{5}\theta+a_{6}\xi+a_{7}+a_{8}\cosh\left( \kappa y \right) \right]=0, \\ H\left( \theta,q \right)=L\left( \theta\right)-L\left( \theta_{0} \right)+qL\left( \theta_{0} \right)+q\left[ \phi_{2}\left( \left( \frac{\partial\theta}{\partial y} \right)^{2}+\theta\frac{\partial^{2}\theta}{\partial y^{2}} \right)+a_{15}\left( 1-\phi_{1}\theta\right)\left( \frac{\partial w}{\partial y} \right)^{2}+N_{b}\frac{\partial\xi}{\partial y}\frac{\partial\theta}{\partial y}+N_{t}\left( \frac{\partial\theta}{\partial y} \right)^{2} \right]=0, \\ H\left( \xi,q \right)=L\left( \xi\right)-L\left( \xi_{0} \right)+qL\left( \xi_{0} \right)+q\left[ a_{20} \frac{\partial^{2}\theta}{\partial y^{2}} \right]=0. \end{matrix} \right\}$ (ii)

Where, $L=\frac{\partial^{2}}{\partial y^{2}} .$

In this perturbing methodology, the infinite-series for $w$, $\theta$, and $\xi$ are supposed:

$\left. \begin{matrix} w\left( x,y \right)=w_{0}+qw_{1}+q^{2}w_{2}+\ldots\\ \theta\left( x,y \right)=\theta_{0}+q\theta_{1}+q^{2}\theta_{2}+\ldots\\ \xi\left( x,y \right)=\xi_{0}+q\xi_{1}+q^{2}\xi_{2}+\ldots\end{matrix} \right\}$ (iii)

On use of homotopy perturbing strategy, substituting $q$ =1, we obtain

$\left. \begin{matrix} w\left( x,y \right)=w_{0}+w_{1}+w_{2}+\ldots\\ \theta\left( x,y \right)=\theta_{0}+\theta_{1}+\theta_{2}+\ldots\\ \xi\left( x,y \right)=\xi_{0}+\xi_{1}+\xi_{2}+\ldots\end{matrix} \right\}$ (iv)

By substituting equations (iii) into (ii) and aligning the coefficients of like powers of $q$, we can establish a system of linear correlations. The velocity, temperature, and concentration fields can be determined by solving these coupled linear equations and substituting the solutions (̂$w_{0}, w_{1}, w_{2},\ldots$, $\theta_{0}, \theta_{1},\theta_{2},\ldots$, $\xi_{0}, \xi_{1}, \xi_{2},\ldots$) into equations (iv). With slight adjustments, the velocity, temperature, and concentration fields distributions are expressed as follows:

$w$=$a_{11}+a_{13}+a_{2}+a_{27}+a_{9}-\frac{y^{2}}{2}+a_{12}y^{2}+a_{25}y^{2}+a_{10}y^{4}+a_{24}y^{4}+a_{23}y^{6}+a_{22}y^{8}-a_{14}\cosh(y\kappa)+a_{28}\cosh(y\kappa)+a_{26}y^{2}\cosh(y\kappa)+a_{29}y\sinh(y\kappa)$ ,

$\theta$=$a_{19}+a_{36}+a_{18}y^{2}+a_{34}y^{2}+a_{17}y^{4}+a_{33}y^{4}+a_{16}y^{6}+a_{32}y^{6}+a_{31}y^{8}+a_{30}y^{10}+\frac{1}{2}(2-h^{2}+y^{2})+a_{37}\cosh(y\kappa)+a_{35}y^{2}\cosh(y\kappa)+a_{39}y\sinh(y\kappa)+a_{38}y^{3}\sinh(y\kappa)$,

$\xi$ = $a_{21}+a_{4}+a_{40}-\frac{y^{2}}{2}-a_{18}a_{20}y^{2}-a_{21}y^{2}-a_{17}a_{20}y^{4}-a_{16}a_{20}y^{6}$ .

where $a_{2}, a_{4}, a_{9}-a_{14}, a_{16}-a_{40}$are provided in Appendix.

1. **Additional Results and Graphical Interpretations:**

Fig. 1 illustrates how axial velocity $w(y)$ changes for different Brinkman numbers $Br=0.1 and 0.6$ across various cilia lengths $\gamma$. For each length of cilia, an increase in $Br$ results in an improved velocity profile. This phenomenon is anticipated because a larger Brinkman number signifies a higher level of viscous dissipation compared to thermal conduction, which injects thermal energy into the system and enhances fluid movement. In physical terms, an elevated $Br$ decreases viscous resistance by invigorating the fluid, leading to higher velocities, particularly near the center of the channel. However, similar to earlier observations, longer cilia lengths diminish velocity due to increased surface drag and obstructions. Importantly, the difference in velocity between $Br=0.1 and 0.6$ is more pronounced when γ = 0.0, indicating that the impact of thermal dissipation is most pronounced in the absence of ciliary resistance. At γ = 0.5, the velocity profiles nearly coincide, suggesting that longer cilia mitigate the advantages of increased Brinkman numbers, as mechanical interference supersedes thermal influences.

Fig. 2(a) illustrates how the electro-osmotic parameter $\kappa$ affects the temperature profile $\theta(y)$ for varying cilia lengths $\gamma$. For every value of $\gamma$, increasing $\kappa$ leads to a significant rise in fluid temperature. This phenomenon can be understood as a consequence of a higher $\kappa$, which indicates a thinner electric double layer that strengthens the electric field within the fluid. The increased strength of the electric field boosts the electro-osmotic parameter, resulting in enhanced fluid movement and internal shear. The rise in shear rate produces greater viscous dissipation, transforming mechanical energy into thermal energy and elevating the temperature. This effect is most significant when $\gamma=0$, where fluid flow faces minimal obstruction. As $\gamma$ approaches 0.5, the longer cilia hinder fluid motion, thereby decreasing energy generation and reducing the temperature. Consequently, temperature rises with an increase in $\kappa$ due to intensified electric forcing and internal fluid dynamics, while the presence of cilia counteracts this effect by dampening the flow. Fig. 2(b) depicts how the thermal Grashof number $Gr$ influences the temperature distribution $\theta(y)$ for various ciliary lengths $\gamma$. In all scenarios, raising $Gr$ leads to a higher fluid temperature along the channel. This phenomenon occurs because an increased $Gr$ intensifies thermal buoyancy forces, facilitating upward fluid movement and improving convective heat transfer. Consequently, a greater amount of thermal energy is spread throughout the channel, resulting in an elevated temperature profile. This effect is most pronounced at $\gamma=0$, where there is no obstruction from cilia. For $\gamma=0.1$, the rise in temperature is still noticeable but slightly diminished due to the additional resistance. At $\gamma=0.5$, the temperature profiles are the lowest, and the difference between the two $Gr$ values is reduced, indicating that longer cilia considerably impede buoyancy-driven flow and constrain thermal enhancement. Thus, as ciliary length increases, the impact of $Gr$ on temperature becomes less significant.

Fig. 3(a) illustrates how the Casson parameter $\beta$ affects the concentration distribution $\xi(y)$ for different cilia lengths $\gamma$. An increment in $\beta$ results in a significant rise in nanoparticle concentration throughout the domain for all cilia lengths. This occurs because a higher $\beta$ indicates a lower yield stress in the Casson fluid, which allows for smoother and more uniform fluid deformation. Consequently, these improved flow conditions enhance the suspension of nanoparticles and diminish their likelihood of settling or escaping. Moreover, as $\gamma$ increases, the concentration profile rises further, suggesting that longer cilia create mechanical resistance that retains more particles within the channel. In summary, the combination of reduced yield stress and ciliary resistance contributes to a higher nanoparticle concentration by enhancing flow accessibility and limiting convective losses. Fig. 3(b) illustrates how the variable thermal conductivity parameter $\phi_{2}$ affects the nanoparticle concentration profile $\xi(y)$ for different cilia lengths $\gamma$. It is noted that an increase in $\phi_{2}$ consistently results in a decrease in concentration throughout the entire domain, independent of cilia length. This happens because a higher $\phi_{2}$ enhances thermal conductivity, enabling heat to distribute more efficiently and diminishing the local temperature gradients that facilitate thermophoretic particle accumulation. With reduced thermophoretic forces, nanoparticles tend to diffuse outward, leading to a decrease in their concentration within the flow. Although longer cilia still contribute to retention by limiting fluid movement, the overriding influence of increased thermal conductivity at elevated $\phi_{2}$ values results in an overall reduction in concentration. This demonstrates that raising $\phi_{2}$ encourages thermal diffusion at the expense of nanoparticle retention.

In Fig. 4(a), the Brinkman number 𝐵𝑟 presents a similar two-stage behavior: $C_{f}$ initially shows a slight increase at low 𝐵𝑟, indicating that minimal viscous dissipation enhances shear near the wall, but it drops sharply at higher 𝐵𝑟, where internal heating prevails and significantly diminishes wall friction. The surfaces are raised vertically with increasing 𝛾, illustrating that longer cilia consistently elevate $C_{f}$, although their effect diminishes under strong dissipation conditions. In Fig. 4(b), the response of $C_{f}$ to the mass Grashof number (𝐺𝑚) indicates the effect of solutal buoyancy. At moderate values of 𝐺𝑚, $C_{f}$ increases, particularly in the upstream region, driven by increased upward convective motion that augments near-wall gradients. However, at elevated 𝐺𝑚, the flow begins to become more uniformly distributed due to strong concentration-driven buoyancy, flattening the surface and resulting in a decrease in $C_{f}$. Even in this case, longer cilia maintain a generally higher wall friction profile.

Fig. 5(a) depicts the impact of the inclination angle ($\alpha$), indicating that $Nu$ sharply rises with higher $\alpha$ close to the inlet, followed by a slight dip and eventual recovery downstream. This pattern is a result of buoyancy-enhanced convection, and longer cilia ($\gamma$) strengthen this convective mechanism via enhanced fluid-wall interactions. In Fig. 5(b), the Brownian motion parameter $(Nb)$ boosts $Nu$, particularly in the entrance region, due to enhanced nanoparticle diffusion. Longer cilia contribute to stronger convective mixing, which combines with Brownian motion to heighten wall heat transfer. Conversely, Lastly, Fig. 5(c) shows that the thermophoresis parameter $(Nt)$ boosts $Nu$, especially in the upstream region, as thermophoretic forces thin out the thermal boundary layer. Again, higher $\gamma$ leads to a more significant enhancement in convection. Throughout most figures, it is clear that an increase in the cilia length parameter $(\gamma)$ improves convective heat transfer by facilitating fluid mixing and thermal dispersion. However, the findings also reveal that in certain dissipation-dominated conditions-such as those characterized by a high Brinkman number-the positive impact of cilia may be diminished or even reversed. Consequently, while $(\gamma)$ typically serves as a crucial factor in optimizing heat transfer performance, its effectiveness significantly relies on the prevailing energy transport mechanisms within the flow.

Fig. 6(a) indicates that an increase in the viscosity parameter $(\phi_{1})$ slightly decreases $Sh$, especially in the central channel region, due to the thickening of the velocity boundary layer that reduces solute flux; longer cilia introduce spatial asymmetry, further modifying the concentration distribution. In Fig. 6(b), the Brinkman number $(Br)$, associated with viscous dissipation, initially boosts $Sh$ but ultimately leads to a decline downstream as internal heating diminishes the concentration gradient at the wall. In this scenario, the influence of cilia becomes less significant, as the energy contributed from dissipation outweighs the convective enhancements. Lastly, Fig. 6(c) demonstrates that an increase in the Casson fluid parameter $(\beta)$, which lowers yield stress, tends to decrease $Sh$ as the flow becomes more uniform and less driven by shear; nonetheless, longer cilia promote localized solute transport through intensified stirring, which is evident in the enhanced surface contours. Together, the figures illustrate that while ciliary motion generally enhances mass transfer by disrupting solute layers, its efficiency depends on the relative strengths of competing physical mechanisms, such as thermophoresis, viscous heating, and rheological resistance.


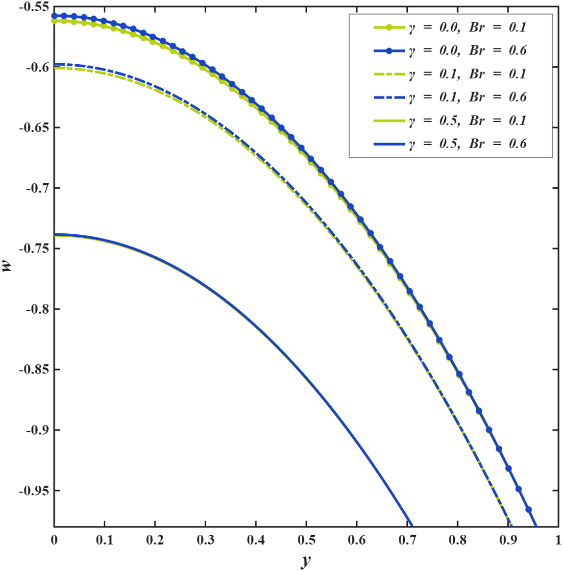


**Figure 1:** Effect of cilia lengths ($\gamma$) on axial velocity profiles $w(y)$ for Brinkman number ($Br$).

| 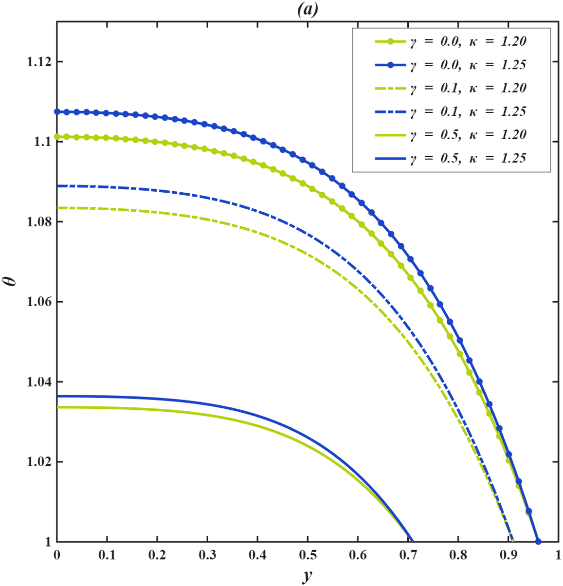 | 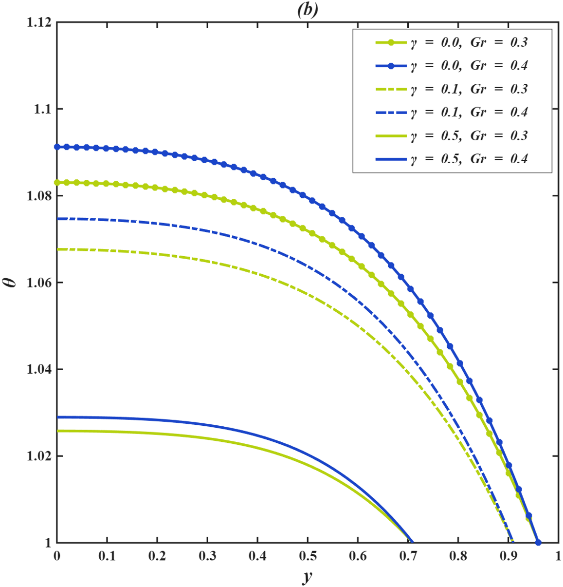 |
| --- | --- |

**Figure 2:** Effect of cilia lengths ($\gamma$) on temperature profiles $\theta(y)$ under varying physical parameters: (a) electroosmotic parameter ($\kappa$), (b) thermal Grashof number ($Gr$).

| 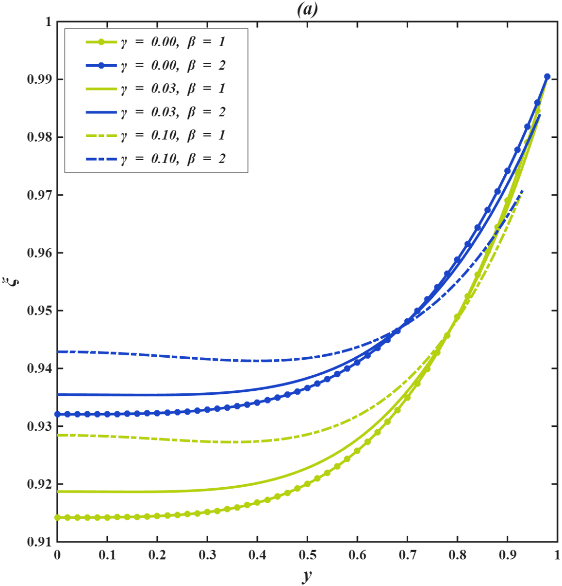 | 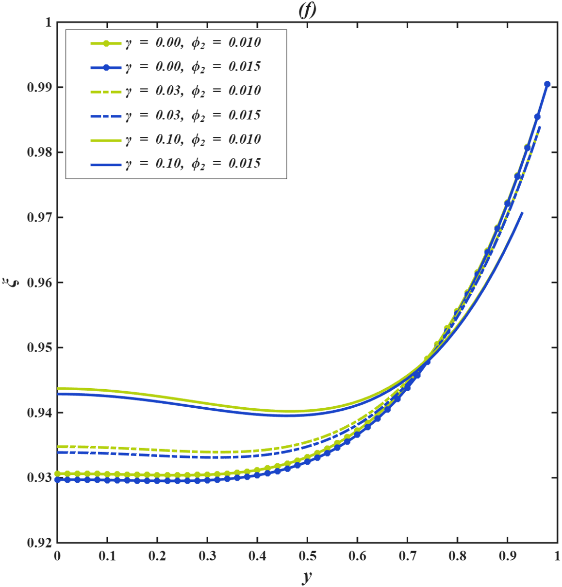 |
| --- | --- |

**Figure 3:** Effect of cilia lengths ($\gamma$) on concentration profiles $\xi(y)$ under varying physical parameters: (a) Casson fluid parameter ($\beta$), (b) thermal conductivity variation parameter ($\phi_{2}$).

| 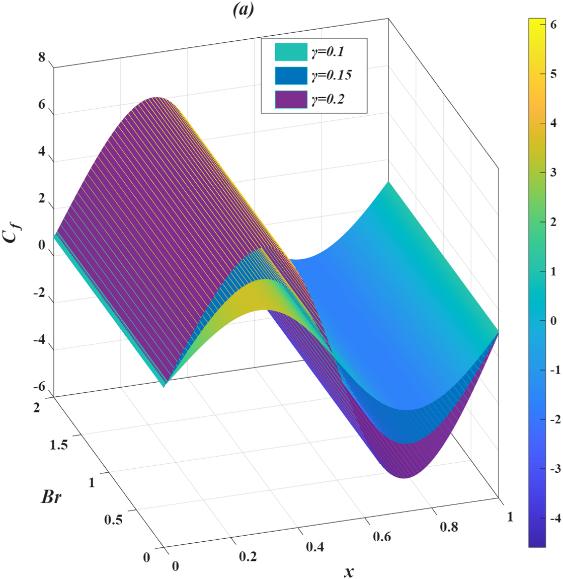 | 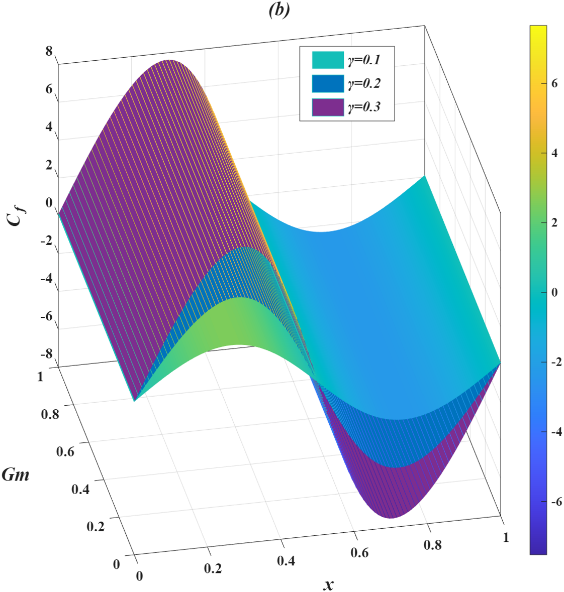 |
| --- | --- |

**Figure 4:** Variation of skin friction coefficient ($C_{f}$) with cilia lengths ($\gamma$) across different flow parameters: (a) Brinkman number ($Br),$(b) mass Grashof number ($Gm$).

| 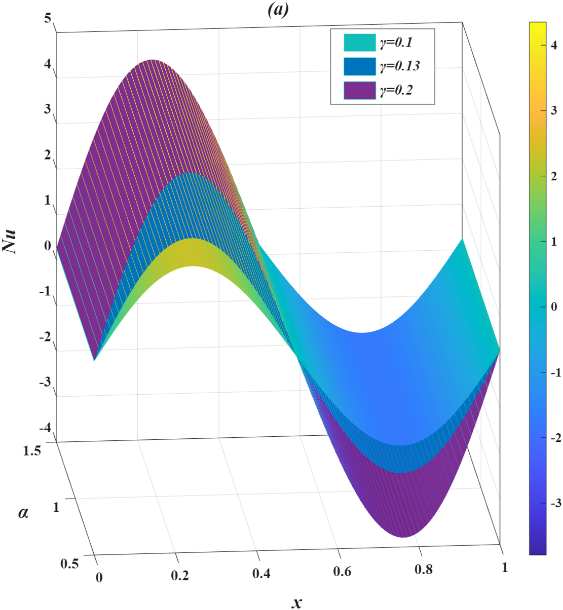 | 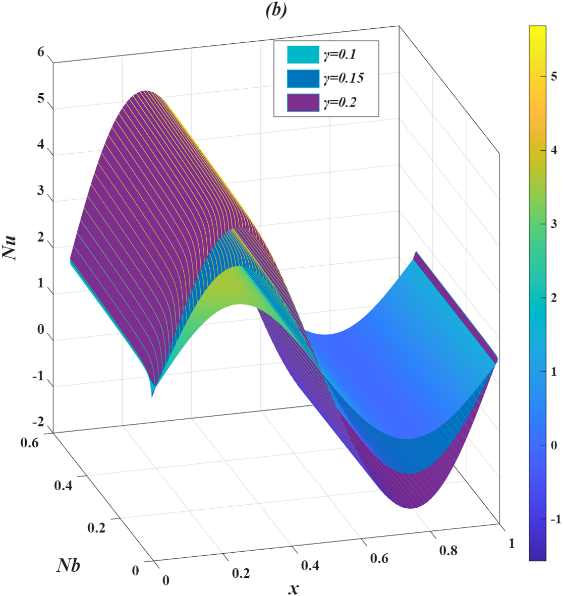 |
| --- | --- |


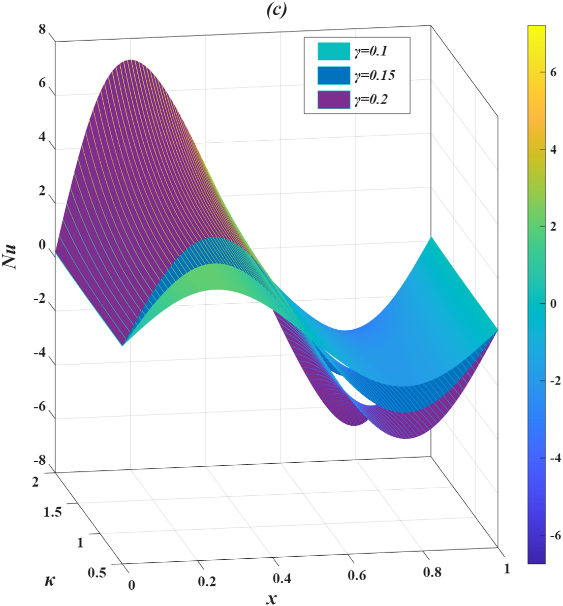


**Figure 5:** Variation of Nusselt number (*Nu*) with cilia lengths ($\gamma$) across different parameters: (a) inclination angle ($\alpha$), (b) Brownian motion parameter ($Nb$), (c) electroosmotic parameter ($\kappa$).

| 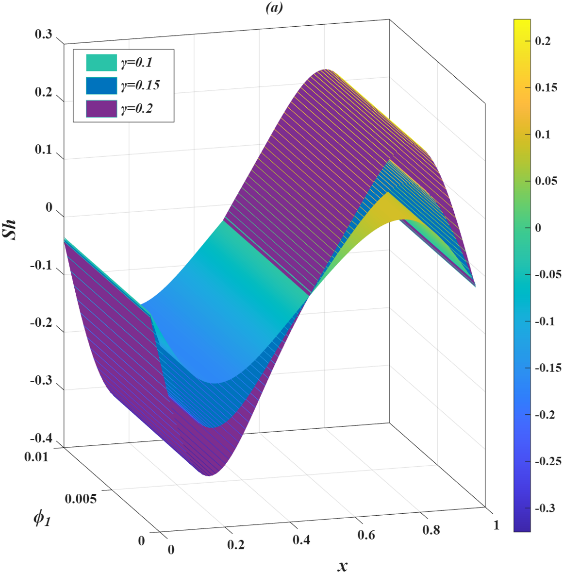 | 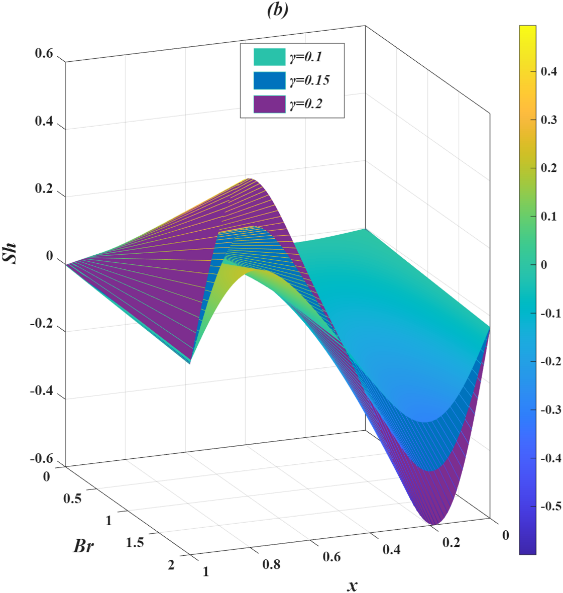 |
| --- | --- |


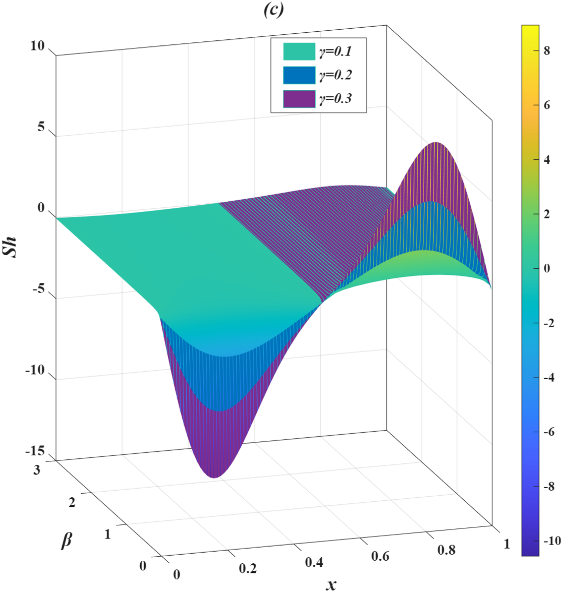


**Figure 6:** Variation of Sherwood number (*Sh*) with cilia lengths ($\gamma$) across different parameters: (a) viscosity variation parameter ($\phi_{1}$), (b) Brinkman number ($Br$), and (c) Casson fluid parameter ($\beta$).

**References:**

[1] J. Prakash, A. Sharma, and D. Tripathi, “Thermal radiation effects on electroosmosis modulated peristaltic transport of ionic nanoliquids in biomicrofluidics channel,” *J Mol Liq*, vol. 249, pp. 843–855, Jan. 2018, doi: 10.1016/j.molliq.2017.11.064.

[2] R.-J. Yang, L.-M. Fu, and Y.-C. Lin, “Electroosmotic Flow in Microchannels,” *J Colloid Interface Sci*, vol. 239, no. 1, pp. 98–105, Jul. 2001, doi: 10.1006/jcis.2001.7551.

[3] U. Ghosh and S. Chakraborty, “Electroosmosis of viscoelastic fluids over charge modulated surfaces in narrow confinements,” *Physics of Fluids*, vol. 27, no. 6, Jun. 2015, doi: 10.1063/1.4922585.

[4] P. C. H. Li and D. J. Harrison, “Transport, Manipulation, and Reaction of Biological Cells On-Chip Using Electrokinetic Effects,” *Anal Chem*, vol. 69, no. 8, pp. 1564–1568, Apr. 1997, doi: 10.1021/ac9606564.

[5] D. Gillespie and S. Pennathur, “Separation of Ions in Nanofluidic Channels with Combined Pressure-Driven and Electro-Osmotic Flow,” *Anal Chem*, vol. 85, no. 5, pp. 2991–2998, Mar. 2013, doi: 10.1021/ac400081p.

[6] S. Chakraborty, “Augmentation of peristaltic microflows through electro-osmotic mechanisms,” *J Phys D Appl Phys*, vol. 39, no. 24, pp. 5356–5363, Dec. 2006, doi: 10.1088/0022-3727/39/24/037.

[7] A. Bandopadhyay, D. Tripathi, and S. Chakraborty, “Electroosmosis-modulated peristaltic transport in microfluidic channels,” *Physics of Fluids*, vol. 28, no. 5, May 2016, doi: 10.1063/1.4947115.

[8] H. A. Hosham, E. N. Thabet, A. M. Abd-Alla, and S. M. M. El-Kabeir, “Dynamic patterns of electroosmosis peristaltic flow of a Bingham fluid model in a complex wavy microchannel,” *Sci Rep*, vol. 13, no. 1, p. 8686, May 2023, doi: 10.1038/s41598-023-35410-2.

[9] T. Hayat, S. Nazir, S. Farooq, A. Alsaedi, and S. Momani, “Impacts of entropy generation in radiative peristaltic flow of variable viscosity nanomaterial,” *Comput Biol Med*, vol. 155, p. 106699, Mar. 2023, doi: 10.1016/j.compbiomed.2023.106699.

[10] M. A. Elogail, “Peristaltic flow of a hyperbolic tangent fluid with variable parameters,” *Results in Engineering*, vol. 17, p. 100955, Mar. 2023, doi: 10.1016/j.rineng.2023.100955.

[11] S. A. Hussein, S. E. Ahmed, and A. A. M. Arafa, “Electrokinetic peristaltic bioconvective Jeffrey nanofluid flow with activation energy for binary chemical reaction, radiation and variable fluid properties,” *ZAMM - Journal of Applied Mathematics and Mechanics / Zeitschrift für Angewandte Mathematik und Mechanik*, vol. 103, no. 1, Jan. 2023, doi: 10.1002/zamm.202200284.

[12] Y. Akbar and F. M. Abbasi, “Impact of variable viscosity on peristaltic motion with entropy generation,” *International Communications in Heat and Mass Transfer*, vol. 118, p. 104826, Nov. 2020, doi: 10.1016/j.icheatmasstransfer.2020.104826.

[13] J. C. Umavathi, A. J. Chamkha, and S. Mohiuddin, “Combined effect of variable viscosity and thermal conductivity on free convection flow of a viscous fluid in a vertical channel,” *Int J Numer Methods Heat Fluid Flow*, vol. 26, no. 1, pp. 18–39, Jan. 2016, doi: 10.1108/HFF-12-2014-0385.

[14] M. M. Bhatti, A. Zeeshan, and R. Ellahi, “Heat transfer analysis on peristaltically induced motion of particle-fluid suspension with variable viscosity: Clot blood model,” *Comput Methods Programs Biomed*, vol. 137, pp. 115–124, Dec. 2016, doi: 10.1016/j.cmpb.2016.09.010.

[15] R. D. Alsemiry, R. E. Abo-Elkhair, M. R. Eid, and E. M. Elsaid, “Enhancement efficiency of flow and irreversibility system for MHD Buongiorno’s nanofluid in complex peristaltic tapered channel with electroosmosis forces,” *J Comput Des Eng*, vol. 11, no. 6, pp. 244–259, Nov. 2024, doi: 10.1093/jcde/qwae101.

[16] J. Prakash, A. Sharma, and D. Tripathi, “Thermal radiation effects on electroosmosis modulated peristaltic transport of ionic nanoliquids in biomicrofluidics channel,” *J Mol Liq*, vol. 249, pp. 843–855, Jan. 2018, doi: 10.1016/j.molliq.2017.11.064.

[17] M.-H. Chang and A.-C. Ruo, “Rayleigh–Bénard instability in nanofluids: effect of gravity settling,” *J Fluid Mech*, vol. 950, p. A37, Nov. 2022, doi: 10.1017/jfm.2022.837.

[18] D. Tripathi and O. A. Bég, “A study on peristaltic flow of nanofluids: Application in drug delivery systems,” *Int J Heat Mass Transf*, vol. 70, pp. 61–70, Mar. 2014, doi: 10.1016/j.ijheatmasstransfer.2013.10.044.

[19] P. Dutta, A. Beskok, and T. C. Warburton, “Electroosmotic flow control in complex microgeometries,” *Journal of Microelectromechanical Systems*, vol. 11, no. 1, pp. 36–44, 2002, doi: 10.1109/84.982861.

[20] S. N. Khaderi and P. R. Onck, “Fluid–structure interaction of three-dimensional magnetic artificial cilia,” *J Fluid Mech*, vol. 708, pp. 303–328, Oct. 2012, doi: 10.1017/jfm.2012.306.

[21] E. Lauga, M. Brenner, and H. Stone, “Microfluidics: The No-Slip Boundary Condition,” in *Springer Handbook of Experimental Fluid Mechanics*, Berlin, Heidelberg: Springer Berlin Heidelberg, 2007, pp. 1219–1240. doi: 10.1007/978-3-540-30299-5_19.
